# Divergence Time Determines the Performance of the Fungal Internal Transcribed Spacer (ITS) DNA Barcode in the Lichen-Forming Ascomycete Genus *Parmelina*

**DOI:** 10.3390/jof12100720

**Published:** 2026-09-24

**Authors:** Ruth Del-Prado, David Pizarro, David Alors, Guillermo Amo de Paz, Zahira Rebollo, Marta Sánchez, H. Thorsten Lumbsch, Pradeep K. Divakar

**Affiliations:** 1 Departamento de Farmacología, Farmacognosia y Botánica, Facultad de Farmacia, Universidad Complutense de Madrid, 28040 Madrid, Spain; dapizarr@ucm.es (D.P.); gamopaz@ucm.es (G.A.d.P.); zahirare@ucm.es (Z.R.); martas15@ucm.es (M.S.); 2 Laboratorio de Biología de líquenes, Facultad de Recursos Naturales, Universidad Católica de Temuco, Temuco 478 0694, Chile; dalors@uct.cl; 3 Departamento de Ciencias Biológicas y Químicas, Universidad Católica de Temuco, Temuco 478 0694, Chile; 4 Negaunee Integrative Research Center, Field Museum of Natural History, Chicago, IL 60605, USA; tlumbsch@fieldmuseum.org

**Keywords:** DNA barcoding, ITS, lichen-forming fungi, cryptic diversity, species delimitation, barcode gap, Parmeliaceae, *Parmelina*

## Abstract

Molecular studies have repeatedly demonstrated that morphology-based species circumscriptions often underestimate the diversity of lichenized fungi by masking distinct evolutionary lineages under a single species name. In *Parmelina*, a lichen-forming ascomycete genus whose extant diversity originated during the Miocene and Pliocene, several cryptic species have recently been recognized. Here, we evaluated the performance of the fungal DNA barcode marker, the internal transcribed spacer (ITS) region, in discriminating species that diverged at different evolutionary times. ITS reliably discriminated all the species that originated during the Miocene, for which intra- and inter-lineage distances form a clean, non-overlapping barcode gap (0.0137–0.0162 substitutions/site), but it failed to resolve the two species complexes that radiated during the Pliocene. Barcode-gap analyses based on genetic-distance thresholds previously established for parmelioid lichens produced congruent results, confirming species discrimination among Miocene lineages while highlighting inconsistencies in the current circumscription of two Pliocene species complexes. We also provide a curated ITS DNA barcode reference library comprising 749 accurately identified specimens sampled across the geographic range of *Parmelina*. Expanded taxon sampling further revealed new country records for *P. clandestina* in Greece, Hungary, India, Norway, Slovakia, and Switzerland, and for *P. mediterranea* in France, Greece, Morocco, Turkey, and Switzerland. Our results demonstrate that the discriminatory power of the fungal ITS barcode depends on the evolutionary age of species divergence, successfully identifying older lineages while revealing limitations for more recently diverged taxa. Automated, distance-based delimitation with ABGD and ASAP independently recovered every Miocene lineage, split *P. atricha* into two entities and assigned the whole *P. tiliacea* aggregate to a single partition, corroborating this conclusion.

## 1. Introduction

DNA barcoding (i.e., application of short, standardized DNA sequences to identify species) has noticeably changed how we measure, evaluate, and monitor biodiversity in all ecosystems [1,2]. The basis of species identification by DNA barcoding is a curated barcode reference library, allowing comparisons of DNA sequences from unidentified organisms to sequences from previously identified taxa. DNA barcoding has significantly contributed to our knowledge of species boundaries, accurate biodiversity assessment, cryptic species evaluation, and the composition of biological communities across the world [3,4,5,6]. At present, accurate assessment of species-level diversity is essential not only to systematic research but also for biodiversity biomonitoring and conservation risk assessment of species before they become extinct, especially given current climate change scenarios [7]. However, most organisms, including symbiotic fungi, largely lack DNA barcode references for these purposes.

Lichenized fungi account for approximately one-third of described ascomycete diversity and are important components of terrestrial ecosystems. They play key ecological roles by initiating substrate weathering, contributing to carbon and nitrogen cycling, and serving as valuable bioindicators of environmental pollution in applied ecology [8,9]. But they also synthesize unique and diverse secondary metabolites that are being investigated for their pharmacological interest [10,11,12]. However, the species delimitation in lichen-forming fungi is in a state of flux. Phenotypic criteria have traditionally been used to identify species, but this may be misleading when diagnostic characters are subject to phenotypic plasticity or mask the presence of distinct species within the same or very similar morphology. A growing body of evidence has demonstrated that the morphology-based species circumscription underestimates diversity in lichenized fungi, hiding distinct phylogenetic lineages under a single species name. In some species complexes, re-examination of characters has revealed previously overlooked morphological or chemical characters that support the recognition of these species-level lineages (see, e.g., [13,14,15,16,17,18,19,20,21]). However, there are also cases of cryptic species in which no phenotypical characters could be identified to distinguish distinct lineages, although there have been cases in these lineages that are correlated with distinct biogeographical patterns [14,22,23,24,25,26]. In fact, in the last 20 years many cryptic species have been detected in almost all major lineages of lichen-forming fungi (reviewed by [23,27,28]; see also, for example, [21,29,30,31,32,33,34,35,36]).

For over twenty years, the use of genetic distances using short, standardized gene regions (DNA barcodes) has provided an easy and affordable complementary support for species delimitation, especially useful for detecting overlooked taxa in species complexes and cryptic lineages [1,3,31,33,37,38,39,40,41,42]. Particularly, a threshold between intraspecific and interspecific genetic distances (barcode gap) was established in parmelioid lichens for internal transcribed spacer (ITS) sequences [3], which is the locus universally accepted as the DNA barcode marker for fungal groups including lichenized fungi [43].

The increased availability of genetic data for lichenized fungi has resulted in regional reference databases [44,45] and studies for specific areas [31,42]. However, issues with the ITS barcode marker failing to separate species have been documented in different groups of lichens (e.g., [19,27,46,47]). Leavitt et al. [48] pointed out that without acknowledging the temporal aspect, species delimitation using molecular data will fail across a wide range of scenarios, especially in clades with recent diversification.

In Parmeliaceae, the largest family of lichen-forming fungi, species boundaries have been intensively studied. As a result, species complexes and cryptic species have been reported from different genera [18,24,25,49,50,51,52,53,54,55].

The genus *Parmelina*, which belongs to the parmelioid core of Parmeliaceae, is an example that includes several species that remain taxonomically controversial. It was introduced by Hale [56], segregated from *Parmelia* Acharius, based on morphological and chemical characters. A total of 47 species were included, with *Parmelina tiliacea* designated as the type species. At present, most of the species included by Hale [56,57] have been classified in other genera such as *Canomaculina*, *Myelochroa*, *Parmelinella*, *Parmelinopsis*, *Parmotremopsis* [58] or *Austroparmelina* [23,59]. In its current circumscription, the genus includes eleven accepted species that occur in the Northern Hemisphere, mainly in western Europe, North Africa, the Middle East, India, and western North America [59]. Specifically, seven species are reported from Western Europe: *Parmelina quercina* (Willd.) Hale [57], *P. tiliacea* (Hoffm.) Hale [56], *P. pastillifera* (Harm.) [57], *P. carporrhizans* (Taylor) Poelt & Vezda [60], *P. atricha* (Nyl.) *P. Clerc* [61], *P. cryptotiliacea* A. Crespo & Núñez-Zapata [62], and *P. mediterranea* Barcenas-Peña et al. [63], with three of them extending to Asia (*P. quercina*, *P. tiliacea*, and *P. pastillifera*). *P. clandestina* Barcenas-Peña et al. has been found in Europe and Morocco [63]. Additionally, *P. coleae* Argüello & A. Crespo is the only species restricted to North America [14], *P. gyrophorica* Elix, Sheng L. Wang & J. B. Chen is known only from southern China [64], and *P. yalungana* (Zahlbr.) P.R. Nelson & Kepler from Russia, Alaska, and China [65]. Although multigene phylogenies have repeatedly revealed cryptic species in *Parmelina*, no systematic assessment of the utility of the ITS barcode across the whole genus has been performed.

The species of *Parmelina* are characterized by a foliose thallus with broad, subirregular lobes with rounded tips and marginal cilia, a pored epicortex, cylindrical conidia, and a smooth upper surface that may show whitish maculae. The lower surface is dark coloured with simple rhizines. The upper cortex is a palisade plectenchyma. The apothecia are adnate or sessile, with a maximum but variable diameter of 5 mm. The upper part of the inner exciple is carbonized [59,61,63]. Three species have isidia, whereas the majority of species lack vegetative propagules and usually produce apothecia on the upper surface.

The use of molecular data has repeatedly revealed the presence of distinct phylogenetic lineages under a single species name in *Parmelina*. In 2007, Argüello et al. [14], distinguished four different species in the sexually reproducing *P. quercina* s. lat. based on molecular, morphological, and biogeographical features: *P. quercina*, *P. carporrhizans*, *P. coleae*, and *P. elixia*. The latter has subsequently been shown to belong to a distant clade that is now classified in the Australasian genus *Austroparmelina* [23]. Later, *P*. *atricha* was also segregated from *P. quercina* based on ecological and morphological characters [61]. In subsequent molecular phylogenetic studies, samples of *P. atricha* nested with *P. carporrhizans* in a supported monophyletic clade, in which phylogenetic relationships were not resolved [62,66], suggesting the potential presence of a species complex within *P. carporrhizans* and *P. atricha*, and cryptic lineages in *P. atricha*. For the isidiate *P. tiliacea*, morphologically indistinguishable populations were shown to be genetically distinct, for which the species *P. cryptotiliacea* was described [62]. Additionally, samples of the morphologically distinct *P. pastillifera* are nested within the *P. tiliacea* clade. Hence, subsequent studies addressed the genetic variability within the *P. tiliacea*-*P. pastillifera* group. *P. tiliacea* populations were divided into three genetic clusters without morphological differences among them, and *P. pastillifera* was detected as the fourth cluster [58]. A subsequent study, using genome-wide assessment of single-nucleotide polymorphisms (SNPs) produced by RAD sequencing, separated the samples into four well-supported clades. One clade included the samples of *P. pastillifera*, while the samples of *P. tiliacea* were clustered into three separate clades. These were recognized as *P. tiliacea*, *P. clandestina*, and *P. mediterranea*, respectively [63]. The diversification of the genus happened during the Miocene and Pliocene [66], with the clades undergoing diversification at different times.

Given the aforesaid issue of species delimitation and cryptic species description, the genus is an ideal candidate to test the utility of the DNA barcoding method. We stress that the aim of this study is barcode-based identification, i.e., the assignment of an unknown sequence to an already described species by comparison with a curated reference library, and not formal species delimitation, which requires multiple independent lines of evidence. Genetic distances and automated partitioning methods are used here as diagnostics of the performance of that identification system; where they reveal entities that do not match the current taxonomy, we treat those entities as candidate species requiring further study rather than as delimited species. Specifically, we aim to (1) test the efficiency of the previously established parmelioid ITS barcode gap [3] for the identification of species in *Parmelina*; (2) evaluate whether its performance depends on the evolutionary age of the lineages, given that *Parmelina* diversified during both the Miocene and the Pliocene; and (3) identify candidate species and cryptic lineages within species complexes. Complementarily, we aim to provide a DNA barcode reference library with well-identified specimens.

## 2. Materials and Methods

### 2.1. Taxon Sampling

Sequence data of the ITS region (ITS1, 5.8S and ITS2) were analyzed in 38 specimens of *P. atricha*, 193 of *P. carporrhizans*, 10 of *P. cryptotiliacea*, 59 of *P. pastillifera*, 151 of *P. tiliacea*, 105 of *P. mediterranea*, 154 of *P. clandestina* and 17 of *P. quercina*, collected from distant geographic regions throughout their distributional ranges. Additionally, 18 ITS sequence of *P. coleae* from North America, 3 of *P. yalungana* from Alaska and Russia, and one sample of *Parmelina* sp. from China were included. *Myelochroa irrugans* and *M. metarrevoluta* were selected as outgroups since the genus is closely related [66]. Data from 270 individuals (36.0% of the dataset) were newly generated for this study, while 464 were obtained from our previous studies [3,23,59,62,66] and 15 sequences were retrieved from GenBank. The newly generated sequences originate from Spain (95), Switzerland (64), Portugal (62), Italy (21), Morocco (18) and the USA (10), and are distributed among species as follows: *P. carporrhizans* (179), *P. atricha* (31), *P. tiliacea* (19), *P. clandestina* (12), *P. coleae* (10), *P. pastillifera* (10), *P. mediterranea* (5), *P. quercina* (3) and *P. cryptotiliacea* (1); they markedly improve the representation of *P. carporrhizans* and of the Alpine and Iberian populations of the genus. Newly generated sequences are shown in bold in Table S1 and carry GenBank accession numbers PZ290771–PZ291040.

Specimen identifications were based on diagnostic morphological and chemical characters, although for cryptic species and morphologically ambiguous specimens, final determinations were established using phylogenetic reconstructions of ITS sequence data.

Morphological identification of the samples was carried out by Pradeep K. Divakar, an expert in Parmeliaceae systematics. Voucher specimens have been deposited in the MAF-Lich herbarium, housed in the Faculty of Pharmacy at the Complutense University of Madrid, Spain. MAF (https://www.ucm.es/farmabot/herbario-maf-1, accessed on 21 September 2026) is a public herbarium founded by Blas Lázaro Ibiza in 1892, with its origins in the Herbarium of the Linnean Society of Madrid (Sociedad Linneana Matritensis). Detailed collection information, including voucher numbers and GenBank accession numbers, is provided in Table S1. Specimens were collected in locations outside of national parks or protected areas; therefore, no specific permissions were required. No endangered species were collected during this study.

### 2.2. DNA Extraction, Polymerase Chain Reaction (PCR) Amplification and Sequencing

For the new samples included in this study, total genomic DNA was extracted from freshly collected materials and frozen specimens, using an estimate of 1 cm^2^ of material appearing free of lichenicolous fungi for each sample. Secondary metabolites were removed by soaking the material in methanol for 1–3 h after crushing the specimens [67]. Total DNA was extracted using the SpeedTools Tissue DNA Extraction Kit (Biotools, B & M Labs, S.A., Madrid, Spain), following the manufacturer’s instructions, obtaining two elution aliquots of 50 µL each. Genomic DNA (5–25 ng) was used for PCR amplifications of the ITS region.

Fungal nuclear ITS rDNA was amplified using the primers ITS1-LM [68] and ITS2-KL [69]. Amplifications were performed in a 25 μL volume containing 5 μL of DNA template, plus 12.5 μL of DNA Amplitools MasterMix (Biotools, B & M Labs, S.A., Madrid, Spain), 4.5 μL of distilled water, and 1.5 μL of each primer (10 mM). The amplifications for nuITSrDNA were carried out in an automatic thermocycler (Techne Progene, Jepson Bolton & Co. Ltd., Watford, Hertfordshire, UK) following a touchdown protocol in which the annealing temperature decreased stepwise from 67 °C to 55 °C, followed by 30 cycles at 55 °C and a final extension of 10 min at 72 °C.

Amplification products were visualized on 1% agarose gels stained with SYBR1 Safe DNA (Life Technologies Corporation, Carlsbad, CA, USA) gel stain (10,000× concentrated in DMSO) and subsequently purified using the Biotools PCR Clean-Up kit (Biotools, B & M Labs, S.A., Madrid, Spain) with the following modifications to the manufacturer’s instructions: Distilled water was heated at 65 °C for the final elution. Then 15 μL of PCR product was diluted with 15 μL of distilled water, 60 μL of Buffer B was added, then centrifuged, washed twice with 600 μL of Buffer T3, dried, then 30 μL of pre-heated distilled water was added, incubated for 5 min at 65 °C, and finally eluted. Centrifugation times were doubled.

Both complementary strands were sequenced using the BigDye Terminator reaction kit (ABI PRISM, Applied Biosystems, Foster City, CA, USA). Cycle sequencing reactions were performed with the same sets of primers used in the amplification step. Sequencing reactions were electrophoresed on a 3730 DNA analyzer (Applied Biosystems, Foster City, CA, USA) at Macrogen Spain (Madrid, Spain).

### 2.3. Sequence Alignment

Sequence fragments were assembled and edited using the program SeqMan v.7 (Lasergene R, DNASTAR, Madison, WI, USA). Sequence identity was confirmed using the Mega-BLAST search function in GenBank. Sequences were aligned using the program MAFFT v7 [70], implementing the G-INS-I alignment algorithm and ‘1PAM/K = 2’ scoring matrix, with an offset value of 0.0. Remaining parameters were set to default values. Identical sequences in the resulting 454-position alignment were collapsed into haplotypes, reducing the 749 ingroup sequences to 94 haplotypes. Positions that were gapped or carried ambiguity codes were not considered when assessing identity, so that two sequences differing only at such positions were assigned to the same haplotype. The representative sequence of each haplotype is underlined in Table S1, and the haplotype, clade and subclade to which every specimen belongs are given in additional columns of that table. No haplotype is shared between subclades or between species.

### 2.4. Phylogenetic Analyses

The complete ITS and the haplotype ITS alignment datasets were analyzed using maximum likelihood (ML) and a Bayesian Markov chain Monte Carlo (B/MCMC) approach. ML analyses were performed with IQ-TREE v. 2.0.7 on the IQ-TREE web server (http://iqtree.cibiv.univie.ac.at/, accessed on 21 September 2026) [71,72]. Branch support was assessed with 1000 ultrafast bootstrap (UFBoot) replicates; clades that received UFBoot support equal to or above 70% were considered strongly supported [73].

The ITS data sets were also analyzed with Bayesian inference as implemented in MrBayes v 3.2.7 [74]. The GTR + Gamma model of DNA sequence evolution was selected for both the 749-sequence and the 94-haplotype matrices with the program ModelFinder [75] implemented in IQ-TREE, according to the Bayesian Information Criterion [76], and was applied in both the maximum-likelihood and the Bayesian analyses. No molecular clock was assumed. Two parallel runs of 3 million generations were done, starting with a random tree and employing 8 simultaneous chains each. Every 1000th tree was saved into a file. The first 300,000 generations (i.e., 3000 trees) were deleted as the “burn-in” of the chains. The outputs of MrBayes were examined with the program Tracer v1.7 [77]. Convergence was assessed from the average standard deviation of split frequencies (ASDSF = 0.031 at the end of the run), from the potential scale reduction factor (PSRF = 1.001 for all parameters) and from the effective sample sizes, which exceeded 1700 for every parameter. Topological convergence in the two independent MCMC runs was checked with the “compare” plots in the program AWTY [78]. Posterior probabilities (PPs) of clades were obtained from the 50% majority-rule consensus of sampled trees after excluding the initial 10% as burn-in (3000 trees). Only clades that received posterior probabilities equal to or above 0.95 were considered strongly supported. Phylogenetic trees were drawn using the program FIGTREE v1.4.2 [79]. The sequence alignments and phylogenetic trees have been deposited in Zenodo and are available at https://zenodo.org/records/20664736 (accessed on 21 September 2026) (DOI 10.5281/zenodo.20664735).

### 2.5. Calculation of Genetic Distances

Pairwise ML distances (given as the number of nucleotide substitutions per site) among the ITS rDNA sequences of *Parmelina* species were calculated from the haplotype dataset. Genetic distances were calculated with TREE-PUZZLE 5.2 [80] using the GTR model of nucleotide substitution, assuming a discrete gamma distribution with six rate categories. Positions that were gapped or ambiguous in either sequence of a pair were excluded from that comparison only (pairwise deletion); consequently, haplotypes that differ exclusively at such positions have a distance of zero. The program generates an output file that consists of a triangular matrix with all pairwise distances between all the samples included. All distances were independently recomputed in R v. 4.3.3 with the packages *ape* and *pegas*, and every summary statistic, table and figure reported below was generated programmatically from the alignment; the annotated scripts are deposited together with the data (see Data Availability Statement). Haplotype diversity (Hd) and nucleotide diversity (π) were calculated per species and per subclade with *pegas*. As a check on the substitution model, all distance-based analyses were repeated with Tamura–Nei distances with gamma-distributed rates (TN93 + Gamma, α = 0.455); the two models agreed to three decimal places for every statistic reported here, and both values are given wherever the fourth decimal differs.

Candidate species were proposed based on the threshold of 0.015–0.017 substitutions per site (s/s) that separate intra- and interspecific distances in parmelioid lichens [3]. The operational rule applied throughout is the following: a pairwise distance ≤ 0.015 s/s is treated as intraspecific, a distance ≥ 0.017 s/s as interspecific, and distances falling within the interval (0.015, 0.017) are treated as inconclusive and reported as such, with no taxonomic conclusion drawn from them. Because importing a threshold from a broader taxonomic context requires validation, we also evaluated the threshold empirically within *Parmelina* by comparing the distributions of intra- and inter-lineage distances and by locating the value that minimizes the cumulative error, i.e., the sum of interspecific pairs falling below it and intraspecific pairs falling above it.

### 2.6. Automated Species Delimitation

Two distance-based automated partitioning methods were applied in order to test whether the current species circumscriptions are recovered without a priori assumptions. Automatic Barcode Gap Discovery (ABGD) and Assemble Species by Automatic Partitioning (ASAP) were run on the matrix of all 749 ingroup sequences—rather than on the haplotype matrix, so that the intraspecific component of the distance distribution is represented at its true frequency—using the pairwise distances described above. ABGD was run over the default range of prior intraspecific divergence (*p* = 0.001–0.1, ten steps); ASAP was run with 1000 replicates for the statistical tests, and its ten best-ranked partitions were retained. Both analyses were repeated on the 469 sequences of the *P. tiliacea* aggregate alone. The resulting partitions are given in Table S2.

## 3. Results

### 3.1. Phylogenetic Analyses

Initially, a phylogenetic analysis of the ITS data matrix was performed with 749 sequences that had 454 unambiguously aligned nucleotide positions. In total, 270 sequences were newly generated (Table S1). From this data set we selected one sequence for each haplotype, resulting in a matrix with 94 sequences. For the Bayesian analysis, the LnL value was −2379.6359 with a standard deviation of ±10.7842, and for IQ-TREE analysis, the Log-likelihood value of the consensus tree was −2262.945.

The phylogenetic tree of the entire data set is available in the Supplementary Material (Figure S1); the results of the phylogenetic analysis of the haplotypes are shown in Figure 1. Since the topologies of the trees estimated from ML and Bayesian methods did not present any well-supported conflict, only the ML tree topology is shown, with bootstrap and posterior probability values indicated on the nodes of this tree (Figure 1). All *Parmelina* samples nested in a supported group that split into six supported monophyletic clades. Clade A (only supported in IQ-TREE analysis) included samples of *P. yalungana* (subclade A1) and *Parmelina* sp. from China (subclade A2); clade B included samples of *P. cryptotiliacea*; clade C included samples of *P. quercina*; clade D samples of *P. coleae*; clade E samples of *P. atricha* and *P. carporrhizans*; and clade F samples of “*Parmelina tiliacea* aggregate” [63] (see Figure 1). The sister relationship of clades E and F was supported in IQ-TREE analysis, and clade D formed a strongly supported sister group to the clades F + E. Clade A was resolved as an early-diverging lineage of *Parmelina*; however, the evolutionary relationship was only supported by IQ-TREE analysis. The relationships of clades B and C remained unresolved.

Clade E further split into three supported clusters: subclades E1 and E2 included samples of *P. atricha*, and subclade E3 included samples of *P. carporrhizans*. Within this clade, *P. atricha* did not form a monophyletic group since samples from Spain, France, and Switzerland were grouped in subclade E1 and two samples from France clustered in subclade E2, which formed a sister relationship to *P. carporrhizans*.

Clade F included the samples of the “*P. tiliacea* aggregate”. Within this clade, five monophyletic supported groups were distinguished, although relationships among them were not resolved: subclades F2 and F4 included samples of *P. clandestina*; F5 included all samples of *P. mediterranea* except one; and subclade F3 included samples of *P. tiliacea* together with the clusters F5 and F4. While part of the samples of *P. pastillifera* were nested in subclade F1, the others did not form monophyletic clusters.

In summary, the six main clades are arranged as follows. Clade A, the earliest-diverging lineage, contains *P. yalungana* (A1) and a single specimen from China (A2). Clades B (*P. cryptotiliacea*) and C (*P. quercina*) branch next, with unresolved relationships to each other. Clade D (*P. coleae*) is the strongly supported sister group of clades E + F. Clade E comprises *P. atricha* and *P. carporrhizans* and splits into three supported subclades: E1 (4 haplotypes, 36 specimens), E2 (2 haplotypes, 2 specimens, including the material from the type locality) and E3 (*P. carporrhizans*; 22 haplotypes, 193 specimens); E2 is sister to E3, which renders *P. atricha* paraphyletic. Clade F corresponds to the *P. tiliacea* aggregate (47 haplotypes, 469 specimens) and contains five supported subclades whose mutual relationships are unresolved: F1 (*P. pastillifera*), F2 and F4 (both *P. clandestina*), F3 (*P. tiliacea*) and F5 (*P. mediterranea*). In the 94-haplotype tree, 21 nodes were supported by both criteria (UFBoot ≥ 70% and PP ≥ 0.95) and 20 by bootstrap only; no node was supported by posterior probability alone. Support values for all nodes are given in Table S3.

The ITS data alone were able to resolve the monophyly of five of the ten accepted species of *Parmelina* included here. Samples of *P. atricha* fell into two clades. The four currently accepted species of the “*P. tiliacea* aggregate” could not be separated by ITS data alone.

### 3.2. Genetic Distance Analysis

The intra- and interspecific pairwise genetic distances, sample sizes for each clade/species, number of haplotypes, and mean and range of genetic distances are summarized in Table 1, Table 2, Table 3 and Table 4. Further, the comparison of genetic distances within and between species/clades is depicted in Figure 2 and Figure 3.

Considering only the lineages that diverged during the Miocene (clades A–E, with subclades E1, E2 and E3 treated as separate entities), intra- and inter-lineage distances did not overlap: the largest intra-lineage distance was 0.0137 s/s and the smallest inter-lineage distance 0.0162 s/s (1081 comparisons: 280 intra- and 801 inter-lineage pairs), leaving a clean barcode gap that brackets the parmelioid threshold of 0.015–0.017 s/s. None of these comparisons were misclassified at a threshold of 0.015 s/s, and only two at 0.017 s/s. In contrast, when the *P. tiliacea* aggregate was included, 565 of the 4371 haplotype pairs (12.9%) were misclassified at the 0.015 s/s threshold, 511 interspecific pairs falling below it and 54 intraspecific pairs exceeding it. All intraspecific pairs above 0.017 s/s belonged to only two taxa, *P. atricha* (8 pairs) and *P. clandestina* (37 pairs), and every interspecific pair below 0.015 s/s involved species of the aggregate, in all six of their pairwise combinations (Figure 4).

This heterogeneity is what a single global threshold cannot accommodate, and the threshold-optimization analysis in Figure 4d makes it explicit. Minimizing the cumulative error—interspecific pairs falling below the threshold plus intraspecific pairs falling above it—over all 4371 haplotype pairs places the optimum at 0.0068 s/s (0.0070 under TN93 + Gamma), well below the parmelioid interval, with 229 pairs still misclassified; treating the subclades as separate entities leaves the optimum unchanged and reduces the error to 173 pairs. This low value is driven entirely by the aggregate: because every interspecific pair below 0.015 s/s belongs to it, lowering the threshold removes a large number of false-conspecific assignments at little cost elsewhere. When the within-aggregate comparisons are excluded, the optimum rises to 0.0138 s/s (0.0139 under TN93 + Gamma), adjacent to the published interval, and the cumulative error falls to zero across all 3290 remaining pairs. The best-performing single threshold in *Parmelina* is therefore not a property of the genus but of which lineages are considered: no single value performs well for the Miocene lineages and for the Pliocene aggregate at the same time.

It is worth underlining that the genetic distances for most of the *Parmelina* species or clades fell below the established barcode gap used to delimit species in the parmelioid lichens (0.015–0.017 s/s), except the “*P. tiliacea* aggregate” with distances that ranged from below to above this threshold (Figure 2, Table 1). Within this aggregate, genetic distances within samples of *P. mediterranea* and *P. pastillifera* remained below the established barcode gap (Table 2, Figure 3). In *P. tiliacea*, distances reached a maximum of 0.016, falling within the threshold that separates intra- and interspecific distances (0.015–0.017) (Table 2, Figure 3). Distances within the samples of *P. clandestina* spanned values both below and above the threshold (Table 2, Figure 3). Genetic distances among the four formally described species within the “*P. tiliacea* aggregate” showed wide variation, including values both below and above the established species delimitation threshold in parmelioid lichens (0.015–0.017 s/s). Exceptions were the comparisons of *P. mediterranea*—*P. tiliacea* and *P. mediterranea*—*P. clandestina* (F4), which reached a maximum value of 0.016, remaining within the threshold that separates intra- and interspecific distances (Table 4, Figure 3).

Genetic distances within clades E1 and E2 (*P. atricha*), considered separately, fell below the established barcode gap. However, when all samples of *P. atricha* (clades E1 + E2) were analyzed together, as well as in comparisons between clades E1 and E2, genetic distances exceeded the threshold (Table 1 and Table 3).

Pairwise distances between samples of *P. carporrhizans* (clade E3) and samples of *P. atricha* nested in clade E2 ranged between 0.016 and 0.023 s/s. Therefore, the lowest value falls within the threshold that separates intra- and interspecific distances (0.015–0.017), while higher values coincide with the values established for interspecific distances (Table 3)

In clade A, pairwise genetic distances between samples of *P. yalungana* (A1) and the sample of *Parmelina* from China (A2) exceed the established threshold.

### 3.3. Automated Species Delimitation

ABGD recovered ten groups and ASAP eight and nine subsets in its two best-ranked partitions (asap-scores 2.5 and 4.5). Both methods produced essentially the same result. Every lineage that diverged during the Miocene was recovered exactly as currently circumscribed: *P. yalungana*, *Parmelina* sp., *P. cryptotiliacea*, *P. quercina*, *P. coleae* and *P. carporrhizans* each formed a single, exclusive partition. *P. atricha* was split by both methods into two partitions corresponding to subclades E1 (36 specimens) and E2 (2 specimens). Conversely, all 469 specimens of the *P. tiliacea* aggregate were assigned to a single partition by both methods, so that none of its four currently accepted species was recovered. When the analyses were restricted to the aggregate, ASAP recovered subclade F2 as a distinct entity but did not separate *P. tiliacea*, *P. mediterranea* and *P. clandestina* F4 from one another. The complete partitions are given in Table S2.

## 4. Discussion

Our study, using 749 samples, is one of the largest datasets for DNA barcoding in a group of lichenized fungi so far. We included all species of the genus that are currently known to unambiguously belong to *Parmelina*. Within the dataset, 94 haplotypes were found that were subsequently analyzed to evaluate their phylogeny and barcode gaps among the currently accepted species. Previous molecular and genomic studies have shown that *Parmelina* includes several species that remain taxonomically controversial. Therefore, the specific aim of this paper was to evaluate the efficiency of the previously established parmelioid barcode gap using the ITS region [3] for the rapid identification of species in *Parmelina*. The ITS region is the universal DNA barcode marker of fungi, including lichens [43].

### 4.1. Phylogenetic Relationships

Within the monophyletic genus *Parmelina*, the samples of *P. yalungana*, *P. cryptotiliacea*, *P. quercina*, *P. carporrhizans*, and *P. coleae* formed monophyletic clades, whereas the samples of *P. atricha* were paraphyletic with *P. carporrhizans* nested within. Also, all the samples included in the *P. tiliacea* aggregate [63] grouped in a supported monophyletic clade, but relationships within the group remained poorly resolved. However, it is remarkable that a phylogenetic analysis based solely on the ITS marker is largely consistent, but less resolved, with a recent phylogeny of *Parmelina* based on a multi-gene analysis of five markers [66]. The genus split from its sister-group *Myelochroa* c. 36–44 million years ago (Mya), and the current species started to diversify c. 17–18 Mya [66,81], with most of the diversification occurring in the Miocene, whereas in the *P. tiliacea* aggregate and the clade including *P. carporrhizans*, the diversification extended into the Pliocene [66]. It is known that, also in other clades of lichenized fungi with diversification during the Pliocene, single-locus phylogenies fail to resolve closely related species [30,33,82,83]. Hence, the lack of resolution in the phylogenetic relationships among the two *Parmelina* clades is consistent with results from other studies. The relationships among the species in the *P. tiliacea* aggregate have been resolved previously using restriction site-associated DNA (RAD) markers [63].

Despite these shortcomings of using ITS alone as a phylogenetic marker in this genus, our analysis revealed some interesting results that point out areas where more research is required. For example, *Parmelina* sp. from China, while nested in clade A with samples of *P. yalungana*, as in previous studies [66], was distinct from *P. yalungana*. Nelson et al. [65] segregated *P. yalungana* from *P. quercina* based on phenotypic features and a molecular phylogeny. However, the type of *P. yalungana* has been described to be from China [84], while the samples of *P. yalungana* in this study were collected in Alaska and Russia. It is possible that the specimen here identified as *Parmelina* sp. might represent *P. yalungana* s.str. Additional studies with samples from China are required to identify which species represents *P. yalungana*. Another issue that requires further studies is the species circumscription of *P. atricha*. This species was paraphyletic in the ITS analysis and a previous multi-gene study [66], but monophyletic in a RADseq study [63]. Two samples of *P. atricha* from the type location in La Preste in France clustered with *P. carporrhizans*, whereas the other samples of *P. atricha* were sister to the clade of *P. carporrhizans* + *P. atricha* s.str.

The two recently described cryptic species, *P. clandestina* and *P. mediterranea*, have so far been reported from Germany, Italy, Morocco, Portugal, Slovenia, Spain, and the UK, and from Italy, Spain (including the Canary Islands), and Tunisia, respectively [63]. Our expanded taxon sampling now allows us to report *P. clandestina* for the first time in Greece, Hungary, India, Norway, Slovakia, and Switzerland, and *P. mediterranea* in France, Greece, Morocco, Turkey, and Switzerland (Table S1). These findings indicate that *P. clandestina* is widespread across Europe, North Africa, and India, whereas *P. mediterranea* is primarily distributed in Europe, North Africa, and Turkey.

### 4.2. Genetic Distances

We assembled the largest data set of ITS sequences for a DNA barcoding project so far to test the utility of the fungal barcode marker in the genus *Parmelina*. Our results show that all currently accepted species that diversified during the Miocene can be reliably distinguished using the established barcode gap for parmelioid lichens (0.015–0.017 s/s) [3] (Table 1, Figure 1). In contrast, in the “*P. tiliacea* aggregate” and in the clade including *P. carporrhizans*, where diversification extends into the Pliocene, the ITS barcode marker reveals limitations and underscores the need for additional studies to establish robust species boundaries.

In fact, the genetic distance analysis supports the results of the phylogenetic study that the species delimitation of *Parmelina atricha* requires additional studies. Pairwise genetic distances of the samples from the two subclades (E1 + E2), i.e., considering all the samples of *P. atricha* as a single species, exceed the threshold established in parmelioids, showing values characteristic of interspecific distances (Table 1). However, pairwise genetic distances of the samples from each clade separately follow intraspecific distance values for Parmelioid lichens. Therefore, additional studies with more extensive sampling of *P. atricha*, especially in the type locality and its surroundings, are needed, focusing on morphological and ecological characters that differentiate *P. atricha* from *P. carporrhizans* [61]. Since the type locality of *P. atricha* is in the Eastern Pyrenees, La Preste [85], we considered that clade (E2) as *P. atricha* s.str. However, the case is further complicated by the fact that some specimens from the French Pyrenees cluster together with samples from Switzerland and Spain in *P.* aff. *atricha* (subclade E1). No morphological or chemical differences between these two clades were detected.

While the ITS data were insufficient to fully resolve the phylogenetic relationships among the species of the “*P. tiliacea* aggregate”, the genetic distance analyses revealed values consistent with interspecific distances, supporting the species delimitation introduced by Barcenas-Peña et al. [63]. Notably, samples of *P. clandestina* were split into two separate clades in the phylogenetic analysis. Genetic distance values of all the samples of *P. clandestina* (subclades F2 + F4) exceeded the threshold established for parmelioid lichens, showing values characteristic of interspecific distances (Table 2). These findings suggest that broader genomic analyses with increased taxon sampling may uncover additional, currently unrecognized species.

The two subclades of *P. clandestina* are not only genetically but also geographically segregated. Assigning each of the 154 specimens to its subclade and to its country of origin (Table S1) shows that subclade F4 is overwhelmingly western Mediterranean—96 of its 116 specimens (83%) come from Spain, Portugal and Morocco—and is the only subclade recorded from India, whereas subclade F2 is virtually absent from that region (1 of 38 specimens, 2.6%) and is instead concentrated in central and southern Europe (Italy, Slovenia, Switzerland, Germany) and in south-western Asia (Turkey, Iran). The difference between the two distributions is highly significant (Fisher’s exact test, *p* = 5 × 10^−5^). Nucleotide diversity within *P. clandestina* as currently circumscribed (π = 0.0075) is five-to-six times higher than within either subclade (F2, π = 0.0020; F4, π = 0.0013). Two morphologically indistinguishable, genetically distinct and largely allopatric entities within a single nominal species is a pattern repeatedly reported for cryptic species in lichen-forming fungi [14,22,23,24,25,26], and strengthens the case that *P. clandestina* as currently circumscribed comprises more than one species.

The success of ITS barcoding in *Parmelina* clades that diversified during the Miocene is consistent with findings from numerous other lichenized fungal groups [29,31,53,73,86,87,88]. In some lichen groups, however, the ITS barcode marker was shown to lack the power to separate species (e.g., [19,30,46,47,89]). As discussed in Leavitt et al. [48], the resolution power of a single genetic marker is reduced, especially in clades with recent diversification, as is the case of the “*P. tiliacea* aggregate” clade and of the *P. carporrhizans* + *P. atricha*.

Several non-exclusive mechanisms are expected to underlie this lack of resolution. First, and most simply, too few substitutions have accumulated in a single, relatively short locus since these lineages diverged in the Pliocene. Second, when the interval between successive speciation events is short relative to effective population size, ancestral polymorphism is retained through speciation and lineage sorting remains incomplete, so that any single gene tree, the ITS tree included, is unlikely to match the species tree. Third, the rDNA cistron evolves in a concerted fashion within the tandem array; homogenisation can erase recently acquired diagnostic variation and, where occasional introgression occurs, can transfer ITS variants between closely related lineages. Finally, gene flow between incompletely isolated lineages cannot be excluded, and is plausible for the aggregate given the broad geographic overlap of its species. Resolving these complexes will require genome-wide data from many independent single-copy loci—RADseq, as already applied successfully to this aggregate [63], or target-capture and low-coverage whole-genome approaches—analyzed in a multispecies coalescent framework that explicitly accommodates incomplete lineage sorting, and combined with population-level sampling across the contact zones between the geographically segregated subclades identified here.

In summary, the established parmelioid barcode gap using the ITS region has accurately identified five species, as well as highlighted issues in the current species circumscription within two clades. Our results show that the threshold between intraspecific and interspecific genetic distances (i.e., the barcode gap) established in parmelioid lichens [3] is an effective tool for the identification of species within the genus *Parmelina*. The ITS dataset presented here thus constitutes a robust DNA barcode reference library for the identification of *Parmelina* species distributed worldwide—reliable for the lineages that diverged during the Miocene, and explicitly flagging the two Pliocene complexes in which a single-locus barcode cannot, on present evidence, support species-level identification—and provides a foundation for future species monitoring under changing environmental conditions. To our knowledge, this is the first comprehensive species-level DNA barcode dataset for lichen-forming fungi, based on 749 well-identified specimens sampled across the geographic range of *Parmelina* species.

## Figures and Tables

**Figure 1:**
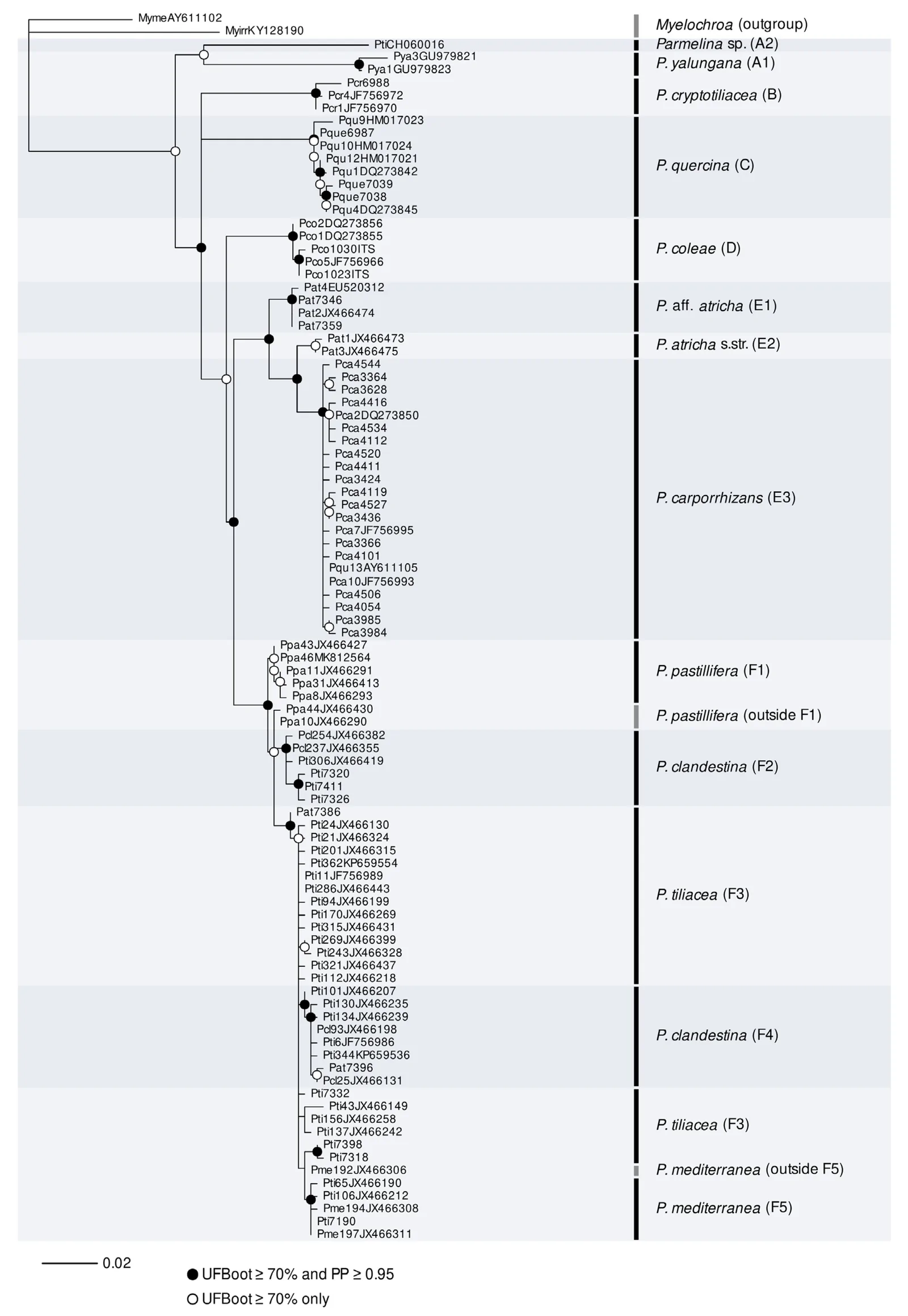
Best-scoring tree from the maximum-likelihood analysis of internal transcribed spacer (ITS) sequences of the 94 *Parmelina* haplotypes; the complete tree of the 749 *Parmelina* samples is presented in Figure S1. Node support is shown with symbols: a filled black circle marks a node supported both by ultrafast bootstrap ≥ 70% and by Bayesian posterior probability ≥ 0.95 (21 nodes), and an open circle a node supported by bootstrap alone (20 nodes). No node was supported by posterior probability alone, and the two nodes created by outgroup rooting, which carry no bootstrap value, are left unmarked. Bars along the right-hand margin delimit the six main clades (A–F) and their subclades and are labelled with the corresponding species; grey bars mark the specimens of *P. pastillifera* and *P. mediterranea* that fall outside subclades F1 and F5 respectively. *Myelochroa irrugans* and *M. metarrevoluta* were used as outgroup. Scale bar: substitutions per site.

**Figure 2:**
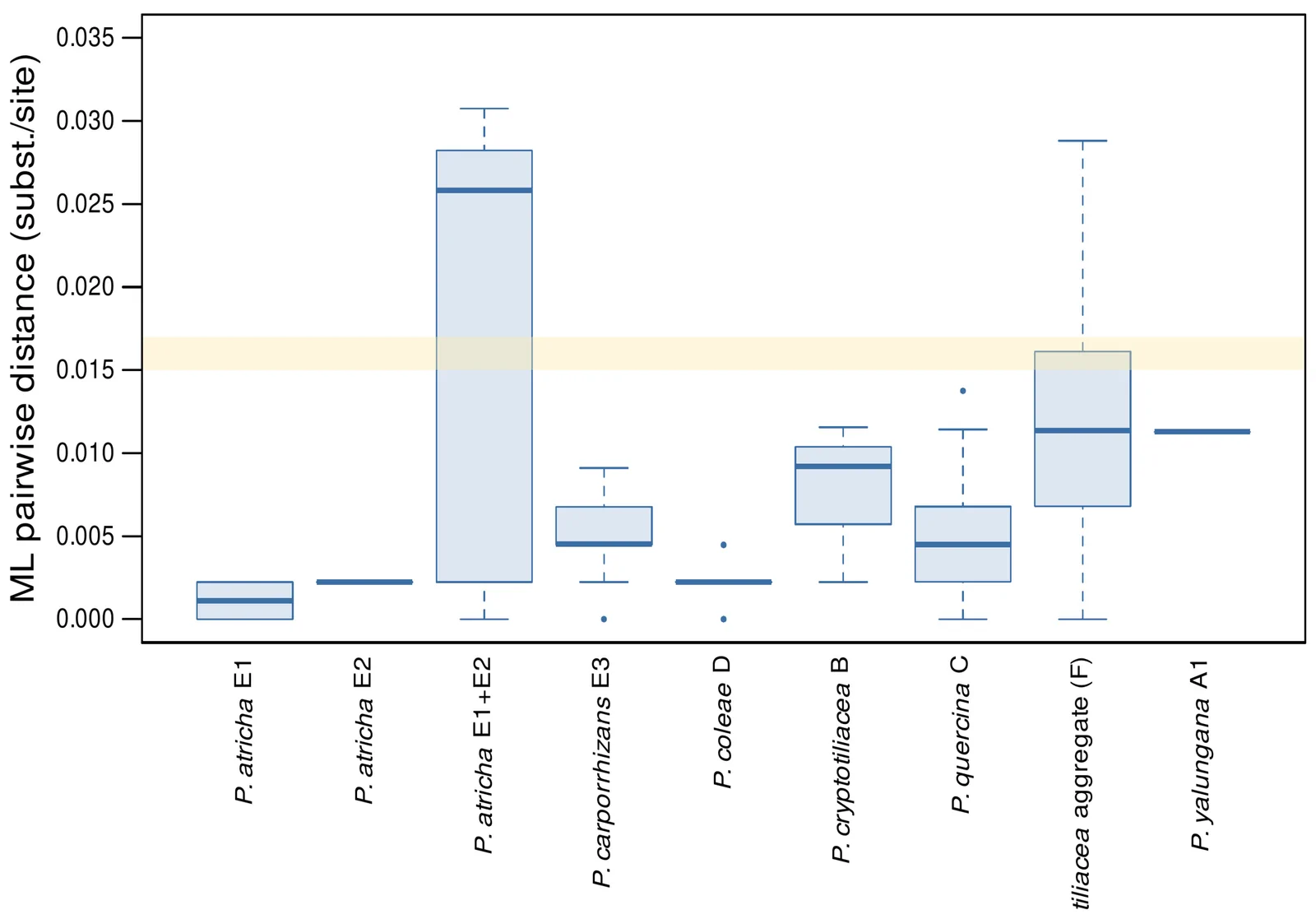
Box plots of pairwise maximum-likelihood distances (substitutions per site) within the species and clades of *Parmelina*, computed from the 94-haplotype matrix. The shaded band marks the interval of 0.015–0.017 s/s that separates intra- from interspecific distances in parmelioid lichens [3]. Only *P. atricha*, when subclades E1 and E2 are pooled into a single species, and the *P. tiliacea* aggregate extend above the band.

**Figure 3:**
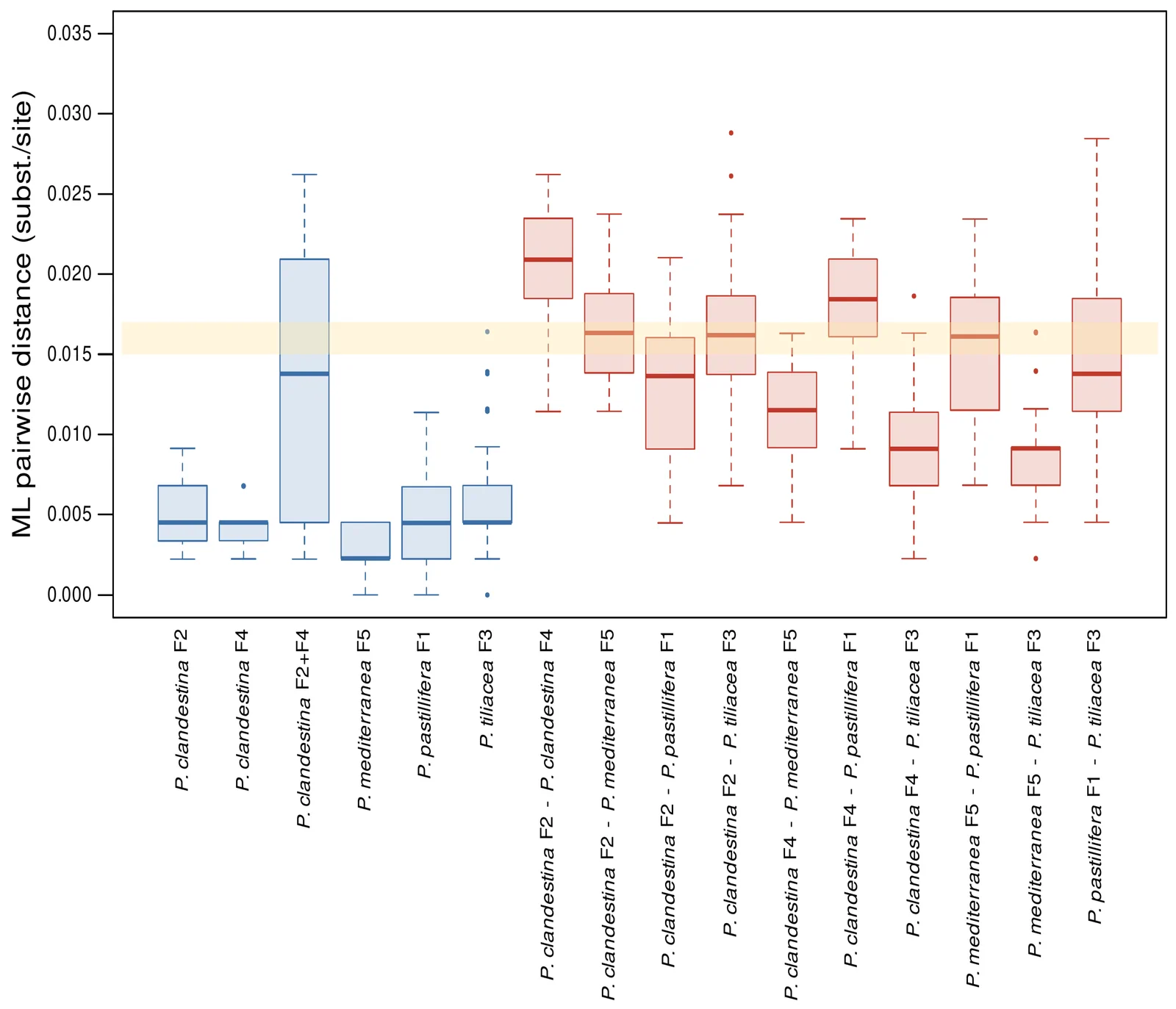
Box plots of pairwise maximum-likelihood distances (substitutions per site) within (blue) and between (red) the formally described species of the “*Parmelina tiliacea* aggregate”. The shaded band marks the threshold of 0.015–0.017 s/s [3]. Distances within *P. clandestina* exceed the threshold when subclades F2 and F4 are pooled, whereas most between-species comparisons fall below it or across it.

**Figure 4:**
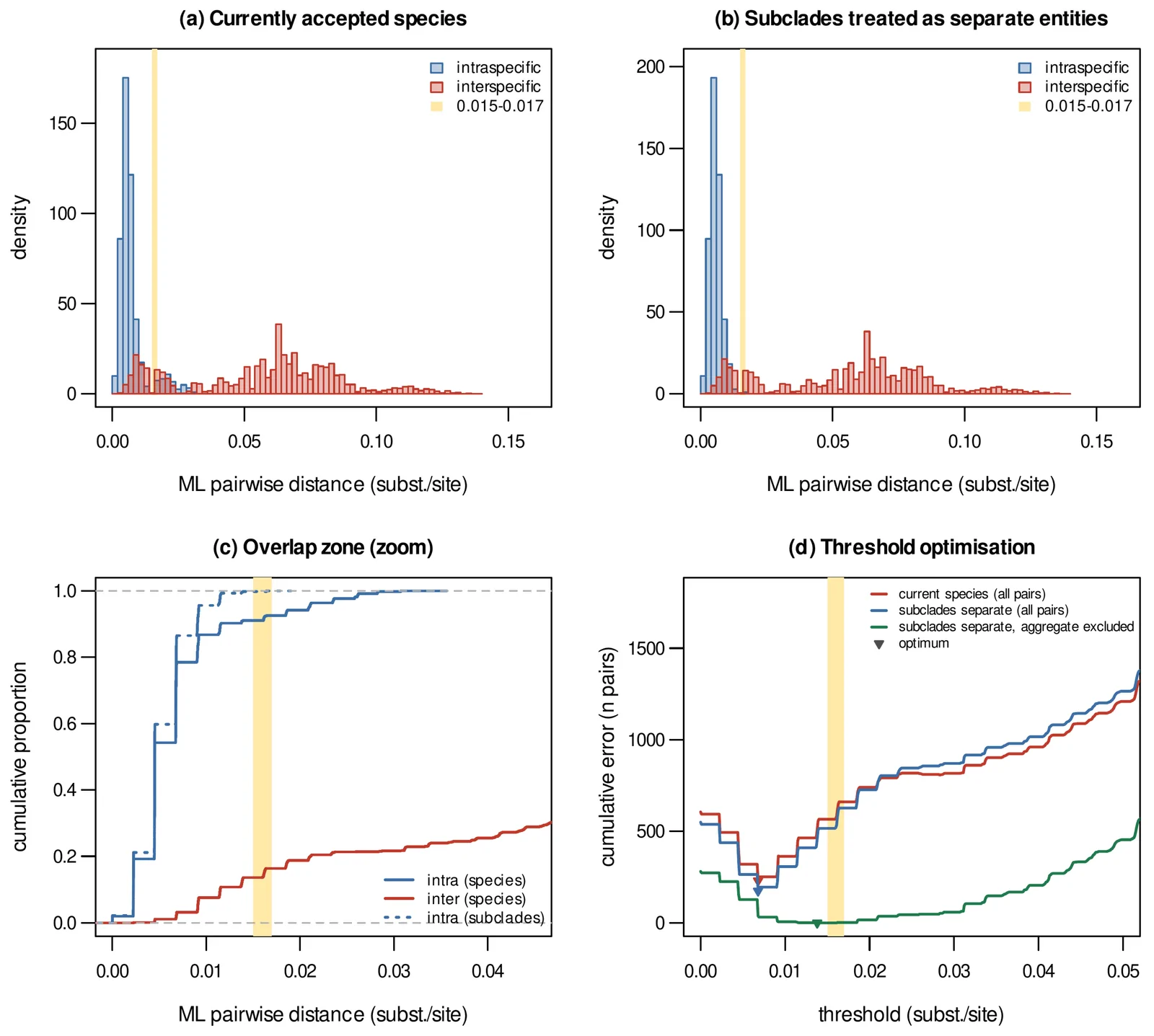
Performance of the ITS barcode gap in *Parmelina*. (**a**) Frequency distribution of intraspecific (blue) and interspecific (red) pairwise distances under the currently accepted species circumscriptions; (**b**) the same distributions when subclades E1, E2, F1–F5 are treated as separate entities; (**c**) cumulative distributions across the zone of overlap; (**d**) threshold optimization: cumulative error—interspecific pairs falling below a given threshold plus intraspecific pairs falling above it—for three comparison sets, with triangles marking each optimum. Over all 4371 haplotype pairs the error is minimized at 0.0068 s/s, well below the parmelioid interval, whether species are taken as currently circumscribed (red) or the subclades are treated as separate entities (blue); excluding the within-aggregate comparisons (green) moves the optimum to 0.0138 s/s and brings the cumulative error to zero. In all panels, the shaded band marks the parmelioid threshold of 0.015–0.017 substitutions per site [3], and treating the subclades as separate entities removes almost every intraspecific distance above it.

**Table 1:** Pairwise genetic distances within *Parmelina* species/clades (substitutions/site).

Species/Clade	N Sequences	N Haplotypes	Range	Mean ± SD	Hd ± SD	π
*P. atricha* E1	36	4	0.000–0.002	0.001 ± 0.001	0.5429 ± 0.0533	0.0011
*P. atricha* E2	2	2	0.002–0.002	0.002 ± NA	1.0000 ± 0.5000	0.0022
*P. atricha* E1 + E2	38	6	0.000–0.031	0.015 ± 0.014	0.5903 ± 0.0568	0.0036
*P. carporrhizans* E3	193	22	0.000–0.009	0.006 ± 0.002	0.5479 ± 0.0396	0.0016
*P. coleae* D	18	5	0.000–0.004	0.002 ± 0.001	0.6732 ± 0.1059	0.0012
*P. cryptotiliacea* B	10	3	0.002–0.012	0.008 ± 0.005	0.3778 ± 0.1813	0.0022
*P. quercina* C	17	8	0.000–0.014	0.005 ± 0.004	0.8162 ± 0.0815	0.0041
*P. tiliacea* aggregate F	469	47	0.000–0.029	0.011 ± 0.006	0.9102 ± 0.0053	0.0087
*P. yalungana* A1	3	2	0.011–0.011	0.011 ± NA	0.6667 ± 0.3143	0.0074
*Parmelina sp*. A2	1	1	-	-	-	-

Haplotypes were assigned by collapsing identical sequences in the 454-position alignment; positions that were gapped or carried ambiguity codes were not considered when assessing identity, so that two sequences differing only at such positions were assigned to the same haplotype. Distances were computed under GTR + Gamma with pairwise deletion, which is why some minimum distances are 0.000. Hd, haplotype diversity; π, nucleotide diversity.

**Table 2:** Pairwise genetic distances within the formally described species in the *P. tiliacea* aggregate (substitutions/site).

Species/Clade	N Sequences	N Haplotypes	Range	Mean ± SD	Hd ± SD	π
*P. clandestina* F2	38	6	0.002–0.009	0.005 ± 0.003	0.5846 ± 0.0740	0.0020
*P. clandestina* F4	116	8	0.002–0.007	0.004 ± 0.002	0.5242 ± 0.0332	0.0013
*P. clandestina* F2 + F4	154	14	0.002–0.026	0.013 ± 0.008	0.7067 ± 0.0263	0.0075
*P. mediterranea* F5	105	6	0.000–0.005	0.003 ± 0.001	0.5599 ± 0.0352	0.0012
*P. pastillifera* F1	59	7	0.000–0.011	0.005 ± 0.003	0.5751 ± 0.0405	0.0035
*P. tiliacea* F3	151	20	0.000–0.016	0.006 ± 0.003	0.7111 ± 0.0365	0.0042

Haplotypes 454. Position alignment; positions that were gapped or carried ambiguity codes were not considered when assessing identity, so that two sequences differing only at such positions were assigned to the same haplotype. Distances were computed under GTR + Gamma with pairwise deletion, which is why some minimum distances are 0.000. Hd, haplotype diversity; π, nucleotide diversity.

**Table 3:** Pairwise genetic distances between *Parmelina species*/clades (substitutions/site).

Comparison	N Pairs	Range	Mean ± SD
*P. atricha* E1—*P. atricha* E2	8	0.026–0.031	0.028 ± 0.002
*P. atricha* E1—*P. carporrhizans*	88	0.029–0.036	0.032 ± 0.002
*P. atricha* E1—*P. coleae*	20	0.044–0.057	0.051 ± 0.003
*P. atricha* E1—*P. cryptotiliacea*	12	0.070–0.089	0.078 ± 0.006
*P. atricha* E1—*P. quercina*	32	0.069–0.084	0.076 ± 0.004
*P. atricha* E1—*P. tiliacea* aggregate	188	0.028–0.052	0.041 ± 0.004
*P. atricha* E1—*P. yalungana*	8	0.109–0.119	0.113 ± 0.004
*P. atricha* E1—*P.* sp.	4	0.104–0.107	0.105 ± 0.002
*P. atricha* E2—*P. carporrhizans*	44	0.016–0.023	0.020 ± 0.002
*P. atricha* E2—*P. coleae*	10	0.057–0.068	0.062 ± 0.003
*P. atricha* E2—*P. cryptotiliacea*	6	0.081–0.090	0.086 ± 0.003
*P. atricha* E2—*P. quercina*	16	0.074–0.085	0.080 ± 0.003
*P. atricha* E2—*P. tiliacea* aggregate	94	0.041–0.063	0.052 ± 0.005
*P. atricha* E2—*P. yalungana*	4	0.112–0.122	0.117 ± 0.004
*P. atricha* E2—*P.* sp.	2	0.110–0.113	0.112 ± 0.002
*P. carporrhizans*—*P. coleae*	110	0.058–0.075	0.068 ± 0.004
*P. carporrhizans*—*P. cryptotiliacea*	66	0.085–0.098	0.092 ± 0.003
*P. carporrhizans*—*P. quercina*	176	0.072–0.090	0.083 ± 0.004
*P. carporrhizans*—*P. tiliacea* aggregate	1034	0.046–0.075	0.063 ± 0.005
*P. carporrhizans*—*P. yalungana*	44	0.117–0.131	0.125 ± 0.004
*P. carporrhizans*—*P.* sp.	22	0.106–0.117	0.112 ± 0.003
*P. coleae*—*P. cryptotiliacea*	15	0.072–0.095	0.082 ± 0.007
*P. coleae*—*P. quercina*	40	0.072–0.090	0.080 ± 0.004
*P. coleae*—*P. tiliacea* aggregate	235	0.038–0.065	0.052 ± 0.005
*P. coleae*—*P. yalungana*	10	0.100–0.118	0.109 ± 0.007
*P. coleae*—*P.* sp.	5	0.097–0.101	0.099 ± 0.002
*P. cryptotiliacea*—*P. quercina*	24	0.076–0.089	0.082 ± 0.003
*P. cryptotiliacea*—*P. tiliacea* aggregate	141	0.065–0.101	0.082 ± 0.007
*P. cryptotiliacea*—*P. yalungana*	6	0.101–0.124	0.111 ± 0.008
*P. cryptotiliacea*—*P.* sp.	3	0.120–0.136	0.126 ± 0.008
*P. quercina*—*P. tiliacea* aggregate	376	0.068–0.090	0.079 ± 0.005
*P. quercina*—*P. yalungana*	16	0.104–0.121	0.113 ± 0.005
*P. quercina*—*P.* sp.	8	0.103–0.113	0.108 ± 0.003
*P. tiliacea* aggregate—*P. yalungana*	94	0.095–0.129	0.112 ± 0.006
*P. tiliacea* aggregate—*P.* sp.	47	0.093–0.117	0.105 ± 0.006
*P. yalungana*—*P.* sp.	2	0.114–0.121	0.118 ± 0.005

Distances were computed under GTR + Gamma with pairwise deletion. N pairs, number of pairwise haplotype comparisons.

**Table 4:** Pairwise genetic distances between the formally described species in the *P. tiliacea* aggregate (substitutions/site).

Comparison	N Pairs	Range	Mean ± SD
*P. clandestina* F4—*P. clandestina* F2	48	0.011–0.026	0.020 ± 0.004
*P. clandestina* F4—*P. mediterranea*	48	0.005–0.016	0.012 ± 0.003
*P. clandestina* F4—*P. pastillifera*	56	0.009–0.023	0.018 ± 0.004
*P. clandestina* F4—*P. tiliacea*	160	0.002–0.019	0.009 ± 0.003
*P. clandestina* F2—*P. mediterranea*	36	0.011–0.024	0.017 ± 0.004
*P. clandestina* F2—*P. pastillifera*	42	0.004–0.021	0.013 ± 0.004
*P. clandestina* F2—*P. tiliacea*	120	0.007–0.029	0.016 ± 0.004
*P. mediterranea*—*P. pastillifera*	42	0.007–0.023	0.015 ± 0.004
*P. mediterranea*—*P. tiliacea*	120	0.002–0.016	0.008 ± 0.003
*P. pastillifera*—*P. tiliacea*	140	0.005–0.028	0.015 ± 0.005

Distances were computed under GTR + Gamma with pairwise deletion. N pairs, number of pairwise haplotype comparisons.

## Data Availability

The datasets generated and/or analyzed during the current study are available in the GenBank (https://www.ncbi.nlm.nih.gov/genbank/, accessed on 21 September 2026), as detailed in Supplementary Materials (Table S1). Newly generated sequences have been deposited in GenBank under accession numbers PZ290771-PZ291040. Voucher specimens have been deposited in the MAF-Lich herbarium (https://www.ucm.es/farmabot/herbario-maf-1, accessed on 21 September 2026), and collection details together with voucher numbers are provided in Table S1. In addition, the sequence alignments and phylogenetic trees have been deposited in Zenodo and are available at https://zenodo.org/records/20664736 (accessed on 21 September 2026) (DOI 10.5281/zenodo.20664735). The same record also contains the annotated R (v. 4.3.3) and Python (v. 3.12.3) scripts used to assign specimens to clades and haplotypes, to recompute the genetic distances, to run the barcode-gap, threshold-optimization and automated delimitation analyses, and to generate Figure 1, Figure 2, Figure 3 and Figure 4, together with the derived tables.

## Supplementary Materials

The following supporting information can be downloaded at https://www.mdpi.com/article/10.3390/jof12100720/s1, Figure S1: Maximum-likelihood phylogenetic tree inferred from the complete ITS dataset of 749 *Parmelina* specimens; Table S1: Specimens of *Parmelina* (and Myelochroa) species used in the present study, including collection data and GenBank accession numbers. Newly generated sequences are shown in bold. Underlined sequence codes indicate haplotypes included in the phylogenetic tree (Figure 1) and are representatives of the sequences listed below them. Table S2: Complete partitions recovered by ABGD and ASAP, both for the 749 ingroup specimens and for the 469 specimens of the *P. tiliacea* aggregate analyzed separately; Table S3: Ultrafast bootstrap and Bayesian posterior probability values for every node of the tree shown in Figure 1.

## References

[1] Hebert P.D.N., Cywinska A., Ball S.L., de Waard J.R. (2003). Biological Identifications through DNA Barcodes. Proc. R. Soc. Lond. B.

[2] Pennisi E. (2019). DNA Barcodes Jump-Start Search for New Species. Science.

[3] Del-Prado R., Cubas P., Lumbsch H.T., Divakar P.K., Blanco O., de Paz G.A., Molina M.C., Crespo A. (2010). Genetic Distances within and among Species in Monophyletic Lineages of Parmeliaceae (Ascomycota) as a Tool for Taxon Delimitation. Mol. Phylogenet. Evol..

[4] Kekkonen M., Hebert P.D.N. (2014). DNA Barcode-based Delineation of Putative Species: Efficient Start for Taxonomic Workflows. Mol. Ecol. Resour..

[5] DeSalle R., Goldstein P. (2019). Review and Interpretation of Trends in DNA Barcoding. Front. Ecol. Evol..

[6] Hartop E., Srivathsan A., Ronquist F., Meier R. (2022). Towards Large-Scale Integrative Taxonomy (LIT): Resolving the Data Conundrum for Dark Taxa. Syst. Biol..

[7] Taberlet P., Bonin A., Zinger L., Coissac E. (2018). Environmental DNA: For Biodiversity Research and Monitoring.

[8] Nimis P.L., Scheidegger C., Wolseley P.A. (2002). Monitoring with Lichens—Monitoring Lichens.

[9] Sancho L.G., Pintado A., Green T.G.A. (2019). Antarctic Studies Show Lichens to Be Excellent Biomonitors of Climate Change. Diversity.

[10] Gómez-Serranillos M.P., Fernández-Moriano C., González-Burgos E., Divakar P.K., Crespo A. (2014). Parmeliaceae Family: Phytochemistry, Pharmacological Potential and Phylogenetic Features. RSC Adv..

[11] Yamamoto Y., Hara K., Kawakami H., Komine M., Upreti D.K., Divakar P.K., Shukla V., Bajpai R. (2015). Lichen Substances and Their Biological Activities. Recent Advances in Lichenology.

[12] Calcott M.J., Ackerley D.F., Knight A., Keyzers R.A., Owen J.G. (2018). Secondary Metabolism in the Lichen Symbiosis. Chem. Soc. Rev..

[13] Divakar P.K., Molina M.C., Lumbsch H.T., Crespo A. (2005). *Parmelia barrenoae*, a New Lichen Species Related to *Parmelia sulcata* (Parmeliaceae) Based on Molecular and Morphological Data. Lichenologist.

[14] Argüello A., Del Prado R., Cubas P., Crespo A. (2007). *Parmelina quercina* (Parmeliaceae, Lecanorales) Includes Four Phylogenetically Supported Morphospecies. Biol. J. Linn. Soc..

[15] Giraud T., Refrégier G., Le Gac M., De Vienne D.M., Hood M.E. (2008). Speciation in Fungi. Fungal Genet. Biol..

[16] Baloch E., Grube M. (2009). Pronounced Genetic Diversity in Tropical Epiphyllous Lichen Fungi. Mol. Ecol..

[17] Leavitt S.D., Esslinger T.L., Divakar P.K., Crespo A., Lumbsch H.T. (2016). Hidden Diversity before Our Eyes: Delimiting and Describing Cryptic Lichen-Forming Fungal Species in Camouflage Lichens (Parmeliaceae, Ascomycota). Fungal Biol..

[18] Molina M.C., Divakar P.K., Goward T., Millanes A.M., Lumbsch H.T., Crespo A. (2017). Neogene Diversification in the Temperate Lichen-Forming Fungal Genus *Parmelia* (Parmeliaceae, Ascomycota). Syst. Biodivers..

[19] Boluda C.G., Rico V.J., Divakar P.K., Nadyeina O., Myllys L., McMullin R.T., Zamora J.C., Scheidegger C., Hawksworth D.L. (2019). Evaluating Methodologies for Species Delimitation: The Mismatch between Phenotypes and Genotypes in Lichenized Fungi (*Bryoria* Sect. *Implexae*, Parmeliaceae). Persoonia.

[20] Crespo A., Rico V.J., Garrido E., Lumbsch H.T., Divakar P.K. (2020). A Revision of Species of the *Parmelia saxatilis* Complex in the Iberian Peninsula with the Description of *P. rojoi*, a New Potentially Relict Species. Lichenologist.

[21] Zhang Y., Clancy J., Jensen J., McMullin R.T., Wang L., Leavitt S.D. (2022). Providing Scale to a Known Taxonomic Unknown—At Least a 70-Fold Increase in Species Diversity in a Cosmopolitan Nominal Taxon of Lichen-Forming Fungi. J. Fungi.

[22] Wirtz N., Printzen C., Lumbsch H.T. (2008). The Delimitation of Antarctic and Bipolar Species of Neuropogonoid *Usnea* (Ascomycota, Lecanorales): A Cohesion Approach of Species Recognition for the *Usnea perpusilla* Complex. Mycol. Res..

[23] Crespo A., Ferencova Z., Pérez-Ortega S., Elix J.A., Divakar P.K. (2010). *Austroparmelina*, a New Australasian Lineage in Parmelioid Lichens (Parmeliaceae, Ascomycota). Syst. Biodivers..

[24] Molina M.C., Del-Prado R., Divakar P.K., Sánchez-Mata D., Crespo A. (2011). Another Example of Cryptic Diversity in Lichen-Forming Fungi: The New Species *Parmelia mayi* (Ascomycota: Parmeliaceae). Org. Divers. Evol..

[25] Kirika P.M., Divakar P.K., Crespo A., Lumbsch H.T. (2019). Molecular and Phenotypical Studies on Species Diversity of *Hypotrachyna* (Parmeliaceae, Ascomycota) in Kenya, East Africa. Bryologist.

[26] Garrido-Huéscar E., González-Burgos E., Kirika P.M., Boustie J., Ferron S., Gómez-Serranillos M.P., Lumbsch H.T., Divakar P.K. (2022). A New Cryptic Lineage in Parmeliaceae (Ascomycota) with Pharmacological Properties. J. Fungi.

[27] Lumbsch H.T., Leavitt S.D. (2011). Goodbye Morphology? A Paradigm Shift in the Delimitation of Species in Lichenized Fungi. Fungal Divers..

[28] Crespo A., Perez-Ortega S. (2009). Cryptic Species and Species Pairs in Lichens: A Discussion on the Relationship between Molecular Phylogenies and Morphological Characters. An. Jardín Botánico Madr..

[29] Parnmen S., Rangsiruji A., Mongkolsuk P., Boonpragob K., Nutakki A., Lumbsch H.T. (2012). Using Phylogenetic and Coalescent Methods to Understand the Species Diversity in the *Cladia aggregata* Complex (Ascomycota, Lecanorales). PLoS ONE.

[30] Leavitt S.D., Lumbsch H.T., Stenroos S., Clair L.L.S. (2013). Pleistocene Speciation in North American Lichenized Fungi and the Impact of Alternative Species Circumscriptions and Rates of Molecular Evolution on Divergence Estimates. PLoS ONE.

[31] Leavitt S.D., Esslinger T.L., Hansen E.S., Divakar P.K., Crespo A., Loomis B.F., Lumbsch H.T. (2014). DNA Barcoding of Brown *Parmeliae* (Parmeliaceae) Species: A Molecular Approach for Accurate Specimen Identification, Emphasizing Species in Greenland. Org. Divers. Evol..

[32] Lücking R., Dal-Forno M., Sikaroodi M., Gillevet P.M., Bungartz F., Moncada B., Yánez-Ayabaca A., Chaves J.L., Coca L.F., Lawrey J.D. (2014). A Single Macrolichen Constitutes Hundreds of Unrecognized Species. Proc. Natl. Acad. Sci. USA.

[33] Divakar P.K., Leavitt S.D., Molina M.C., Del-Prado R., Lumbsch H.T., Crespo A. (2016). A DNA Barcoding Approach for Identification of Hidden Diversity in Parmeliaceae (Ascomycota): *Parmelia* sensu stricto as a Case Study. Bot. J. Linn. Soc..

[34] Leavitt S.D., Westberg M., Nelsen M.P., Elix J.A., Timdal E., Sohrabi M., St Clair L.L., Williams L., Wedin M., Lumbsch H.T. (2018). Multiple, Distinct Intercontinental Lineages but Isolation of Australian Populations in a Cosmopolitan Lichen-Forming Fungal Txon, *Psora decipiens* (Psoraceae, Ascomycota). Front. Microbiol..

[35] Simon A., Goffinet B., Magain N., Sérusiaux E. (2018). High Diversity, High Insular Endemism and Recent Origin in the Lichen Genus *Sticta* (Lichenized Ascomycota, Peltigerales) in Madagascar and the Mascarenes. Mol. Phylogenetics Evol..

[36] Magain N., Miadlikowska J., Goffinet B., Goward T., Pardo-De la Hoz C.J., Jüriado I., Simon A., Mercado-Díaz J.A., Barlow T., Moncada B. (2023). High Species Richness in the Lichen Genus *Peltigera* (Ascomycota, Lecanoromycetes): 34 Species in the Dolichorhizoid and Scabrosoid Clades of Section *Polydactylon*, Including 24 New to Science. Persoonia.

[37] Hebert P.D.N., Penton E.H., Burns J.M., Janzen D.H., Hallwachs W. (2004). Ten Species in One: DNA Barcoding Reveals Cryptic Species in the Neotropical Skipper Butterfly *Astraptes fulgerator*. Proc. Natl. Acad. Sci. USA.

[38] Zemlak T.S., Ward R.D., Connell A.D., Holmes B.H., Hebert P.D.N. (2009). DNA Barcoding Reveals Overlooked Marine Fishes. Mol. Ecol. Resour..

[39] Hebert P.D.N., deWaard J.R., Landry J.-F. (2010). DNA Barcodes for 1/1000 of the Animal Kingdom. Biol. Lett..

[40] Liu J., Möller M., Gao L., Zhang D., Li D. (2011). DNA Barcoding for the Discrimination of Eurasian Yews (*Taxus* L., Taxaceae) and the Discovery of Cryptic Species. Mol. Ecol. Resour..

[41] Dal Forno M., Lawrey J.D., Moncada B., Bungartz F., Grube M., Schuettpelz E., Lücking R. (2022). DNA Barcoding of Fresh and Historical Collections of Lichen-Forming Basidiomycetes in the Genera Cora and Corella (Agaricales: Hygrophoraceae): A Success Story?. Diversity.

[42] La Torre R.D., Ramos D., Mejía M.D., Neyra E., Loarte E., Orjeda G. (2023). Survey of Lichenized Fungi DNA Barcodes on King George Island (Antarctica): An Aid to Species Discovery. J. Fungi.

[43] Schoch C.L., Seifert K.A., Huhndorf S., Robert V., Spouge J.L., Levesque C.A., Chen W., Bolchacova E., Fungal Barcoding Consortium, Fungal Barcoding Consortium Author List (2012). Nuclear Ribosomal Internal Transcribed Spacer (ITS) Region as a Universal DNA Barcode Marker for Fungi. Proc. Natl. Acad. Sci. USA.

[44] Marthinsen G., Rui S., Timdal E. (2019). OLICH: A Reference Library of DNA Barcodes for Nordic Lichens. BDJ.

[45] Vondrák J., Svoboda S., Košnar J., Malíček J., Šoun J., Frolov I., Svensson M., Novotný P., Palice Z. (2023). Martin7: A Reference Database of DNA Barcodes for European Epiphytic Lichens and Its Taxonomic Implications. Preslia.

[46] Pino-Bodas R., Martín M.P., Burgaz A.R., Lumbsch H.T. (2013). Species Delimitation in *Cladonia* (Ascomycota): A Challenge to the DNA Barcoding Philosophy. Mol. Ecol. Resour..

[47] Lücking R., Nadel M., Araujo E., Gerlach A. (2020). Two Decades of DNA Barcoding in the Genus Usnea (Parmeliaceae): How Useful and Reliable Is the ITS?. Plant Fungal Syst..

[48] Leavitt S.D., Divakar P.K., Crespo A., Lumbsch H.T. (2016). A Matter of Time—Understanding the Limits of the Power of Molecular Data for Delimiting Species Boundaries. Herzogia.

[49] Crespo A., Lumbsch H.T. (2010). Cryptic Species in Lichen-Forming Fungi. IMA Fungus.

[50] Divakar P.K., Figueras G., Hladun N.L., Crespo A. (2010). Molecular Phylogenetic Studies Reveal an Undescribed Species within the North American Concept of *Melanelixia glabra* (Parmeliaceae). Fungal Divers..

[51] Singh G., Dal Grande F., Divakar P.K., Otte J., Leavitt S.D., Szczepanska K., Crespo A., Rico V.J., Aptroot A., Cáceres M.E.D.S. (2015). Coalescent-Based Species Delimitation Approach Uncovers High Cryptic Diversity in the Cosmopolitan Lichen-Forming Fungal Genus *Protoparmelia* (Lecanorales, Ascomycota). PLoS ONE.

[52] Alors D., Lumbsch H.T., Divakar P.K., Leavitt S.D., Crespo A. (2016). An Integrative Approach for Understanding Diversity in the *Punctelia rudecta* Species Complex (Parmeliaceae, Ascomycota). PLoS ONE.

[53] Del-Prado R., Divakar P.K., Lumbsch H.T., Crespo A.M. (2016). Hidden Genetic Diversity in an Asexually Reproducing Lichen Forming Fungal Group. PLoS ONE.

[54] Kirika P.M., Divakar P.K., Buaruang K., Leavitt S.D., Crespo A., Gatheri G.W., Mugambi G., Benatti M.N., Lumbsch H.T. (2017). Molecular Phylogenetic Studies Unmask Overlooked Diversity in the Tropical Lichenized Fungal Genus *Bulbothrix* s.l. (Parmeliaceae, Ascomycota). Bot. J. Linn. Soc..

[55] Kirika P.M., Lumbsch H.T., Huéscar E.G., Quedensley T.S., Divakar P.K. (2022). *Canoparmelia texana* (Parmeliaceae, Ascomycota) Consists of Two Independent Lineages. Lichenologist.

[56] Hale M.E. (1974). *Bulbothrix*, *Parmelina*, *Relicina* and *Xanthoparmelia*, Four New Genera in the Parmeliaceae (Lichenes). Phytologia.

[57] Hale M.E. (1976). A Monograph of the lichen genus *Parmelina* Hale (Parmeliaceae). Smithson. Contrib. Bot..

[58] Elix J.A., Hale M.E. (1987). *Canomaculina*, *Myelochroa, Parmelinella*, *Parmelinopsis* and *Parmotremopsis*, Five New Genera in the Parmeliaceae (Lichenized Ascomycotina). Mycotaxon.

[59] Nuñez-Zapata J. (2013). Genetic Variability, Cryptic Species and Molecular Phylogeny in the Lichen-Forming Fungal Genus Parmelina (Parmeliaceae, Ascomycota).

[60] Coppins B.J. (1978). Bestimmungsschlüssel Europäischer Flechten. Erganzungsheft I. By J Poelt and A. Vězda [Bibliotheca Lichenologica No. 9.] Cramer, Vaduz. October 1977. Pp. v + 258. Price DM 50 [about £12] [Subscription Rate DM 40]. Lichenologist.

[61] Clerc P., Truong C. (2008). The Non-Sorediate and Non-Isidiate *Parmelina* Species (Lichenized Ascomycetes, Parmeliaceae) in Switzerland—*Parmelina atricha* (Nyl.) P. Clerc Reinstated in the European Lichen Flora. Sauteria.

[62] Núñez-Zapata J., Divakar P.K., Del-Prado R., Cubas P., Hawksworth D.L., Crespo A. (2011). Conundrums in Species Concepts: The Discovery of a New Cryptic Species Segregated from *Parmelina tiliacea* (Ascomycota: Parmeliaceae). Lichenologist.

[63] Barcenas-Peña A., Divakar P.K., Crespo A., Nuñez-Zapata J., Lumbsch H.T., Grewe F. (2023). Reference-Based Restriction-Site-Associated DNA Sequencing Data Are Useful for Species Delineation in a Recently Diverged Asexually Reproducing Species Complex (Parmeliaceae, Ascomycota). J. Fungi.

[64] Wang S.L., Chen J.B., Elix J.A. (2000). New Species of Parmeliaceae (Lichenized Ascomycotina) from China. Mycotaxon.

[65] Nelson P.R., Kepler R., Walton J., Fankhauser J., Nelson L., Song W.L. (2012). *Parmelina yalungana* Resurrected and Reported from Alaska, China and Russia. Bryologist.

[66] Nuñez-Zapata J., Alors D., Cubas P., Divakar P.K., Leavitt S.D., Lumbsch H.T., Crespo A. (2017). Understanding Disjunct Distribution Patterns in Lichen-Forming Fungi: Insights from *Parmelina* (Parmeliaceae: Ascomycota). Bot. J. Linn. Soc..

[67] Cubero O.F., Crespo A., Fatehi J., Bridge P.D. (1999). DNA Extraction and PCR Amplification Method Suitable for Fresh, Herbarium-Stored, Lichenized, and Other Fungi. Plant Syst. Evol..

[68] Myllys L., Lohtander K., Källersjö M., Tehler A. (1999). Sequence Insertions and ITS Data Provide Congruent Information on *Roccella canariensis* and *R. tuberculata* (Arthoniales, Euascomycetes) Phylogeny. Mol. Phylogenetics Evol..

[69] Lohtander K., Myllys L., Sundin R., Källersjö M., Tehler A. (1998). The Species Pair Concept in the Lichen *Dendrographa leucophaea* (Arthoniales): Analyses Based on ITS Sequences. Bryologist.

[70] Katoh K., Toh H. (2008). Recent Developments in the MAFFT Multiple Sequence Alignment Program. Brief. Bioinform..

[71] Nguyen L.-T., Schmidt H.A., Von Haeseler A., Minh B.Q. (2015). IQ-TREE: A Fast and Effective Stochastic Algorithm for Estimating Maximum-Likelihood Phylogenies. Mol. Biol. Evol..

[72] Trifinopoulos J., Nguyen L.-T., von Haeseler A., Minh B.Q. (2016). W-IQ-TREE: A Fast Online Phylogenetic Tool for Maximum Likelihood Analysis. Nucleic Acids Res..

[73] Kelly L.J., Hollingsworth P.M., Coppins B.J., Ellis C.J., Harrold P., Tosh J., Yahr R. (2011). DNA Barcoding of Lichenized Fungi Demonstrates High Identification Success in a Floristic Context. New Phytol..

[74] Ronquist F., Teslenko M., Van Der Mark P., Ayres D.L., Darling A., Höhna S., Larget B., Liu L., Suchard M.A., Huelsenbeck J.P. (2012). MrBayes 3.2: Efficient Bayesian Phylogenetic Inference and Model Choice Across a Large Model Space. Syst. Biol..

[75] Kalyaanamoorthy S., Minh B.Q., Wong T.K.F., Von Haeseler A., Jermiin L.S. (2017). ModelFinder: Fast Model Selection for Accurate Phylogenetic Estimates. Nat. Methods.

[76] Schwarz G. (1978). Estimating the Dimension of a Model. Ann. Stat..

[77] Rambaut A., Drummond A.J., Xie D., Baele G., Suchard M.A. (2018). Posterior Summarization in Bayesian Phylogenetics Using Tracer 1.7. Syst. Biol..

[78] Nylander J.A.A., Wilgenbusch J.C., Warren D.L., Swofford D.L. (2008). AWTY (Are We There yet?): A System for Graphical Exploration of MCMC Convergence in Bayesian Phylogenetics. Bioinformatics.

[79] Rambaut A. (2014). FigTree.

[80] Schmidt H.A., Strimmer K., Vingron M., Von Haeseler A. (2002). TREE-PUZZLE: Maximum Likelihood Phylogenetic Analysis Using Quartets and Parallel Computing. Bioinformatics.

[81] Divakar P.K., Crespo A., Kraichak E., Leavitt S.D., Singh G., Schmitt I., Lumbsch H.T. (2017). Using a Temporal Phylogenetic Method to Harmonize Family- and Genus-Level Classification in the Largest Clade of Lichen-Forming Fungi. Fungal Divers..

[82] Cornejo C., Chabanenko S., Scheidegger C. (2018). Are Species-Pairs Diverging Lineages? A Nine-Locus Analysis Uncovers Speciation among Species-Pairs of the *Lobaria meridionalis*-Group (Ascomycota). Mol. Phylogenetics Evol..

[83] Divakar P.K., Wei X., McCune B., Cubas P., Boluda C.G., Leavitt S.D., Crespo A., Tchabanenko S., Lumbsch H.T. (2019). Parallel Miocene Dispersal Events Explain the Cosmopolitan Distribution of the Hypogymnioid Lichens. J. Biogeogr..

[84] Zahlbruckner A. (1934). Nachtrage Zur Flechtenflora Chinas. Hedwigia.

[85] Nylander J.A.A. (1873). Observata Lichenologica in Pyreneis Orientalibus.

[86] Del-Prado R., Buaruang K., Lumbsch H.T., Crespo A., Divakar P.K. (2019). DNA Sequence-Based Identification and Barcoding of a Morphologically Highly Plastic Lichen Forming Fungal Genus (*Parmotrema*, Parmeliaceae) from the Tropics. Bryologist.

[87] Bradshaw M., Grewe F., Thomas A., Harrison C.H., Lindgren H., Muggia L., Clair L.L.S., Lumbsch H.T., Leavitt S.D. (2020). Characterizing the Ribosomal Tandem Repeat and Its Utility as a DNA Barcode in Lichen-Forming Fungi. BMC Evol. Biol..

[88] Jannah M., Hariri M.R., Kasiamdari R.S., Handayani N.S.N. (2021). The Use of DNA Barcoding and Phylogenetic Analysis to Improve Identification of *Usnea* Spp. Based on ITS rDNA. J. Trop. Biodivers. Biotechnol..

[89] Wei X., McCune B., Lumbsch H.T., Li H., Leavitt S., Yamamoto Y., Tchabanenko S., Wei J. (2016). Limitations of Species Delimitation Based on Phylogenetic Analyses: A Case Study in the *Hypogymnia hypotrypa* Group (Parmeliaceae, Ascomycota). PLoS ONE.

