# Supplementary material for: Divergence Time Determines the Performance of the Fungal Internal Transcribed Spacer (ITS) DNA Barcode in the Lichen-Forming Ascomycete Genus Parmelina
Source: J Fungi (Basel). 2026 Sep 24;12(10):720. doi: 10.3390/jof12100720 (PMC13706601; doi:10.3390/jof12100720)
Supplement: Supplementary file 1 [file jof-12-00720-s001.zip › jof-4488092-supplementary.pdf]

Supplementary Materials

Divergence Time Determines the Performance of the Fungal Internal Transcribed Spacer (ITS) DNA Barcode in the Lichen-Forming Ascomycete Genus *Parmelina*

Ruth Del-Prado, David Pizarro, David Alors, Guillermo Amo de Paz, Zahira Rebollo, Marta Sánchez, H. Thorsten Lumbsch and Pradeep K. Divakar

Journal of Fungi — Manuscript ID jof-4488092

Contents

Figure S1. Maximum-likelihood phylogenetic tree inferred from the complete ITS dataset of 749 *Parmelina* specimens.

Table S1. Specimens of *Parmelina* (and *Myelochroa*) species used in the present study, including collection data and GenBank accession numbers. Newly generated sequences are indicated in the column "Newly generated". The column "Haplotype representative" indicates the sequence representing each haplotype in the phylogenetic tree of Figure 1.

Table S2. Complete partitions recovered by ABGD and ASAP, both for the 749 ingroup specimens and for the 469 specimens of the *P. tiliacea* aggregate analyzed separately.

Table S3. Ultrafast bootstrap and Bayesian posterior probability values for every node of the tree shown in Figure 1.

Figure S1. Maximum-likelihood phylogenetic tree inferred from the complete ITS dataset of 749 *Parmelina* specimens.

Node support follows the notation of Figure 1: a filled circle marks a node supported both by ultrafast bootstrap ≥ 70% and by posterior probability ≥ 0.95, an open circle one supported by bootstrap alone. Nodes left unlabelled in the consensus tree, which group identical or near-identical specimens, carry no symbol. Bars and shading in the right-hand margin mark the subclades.

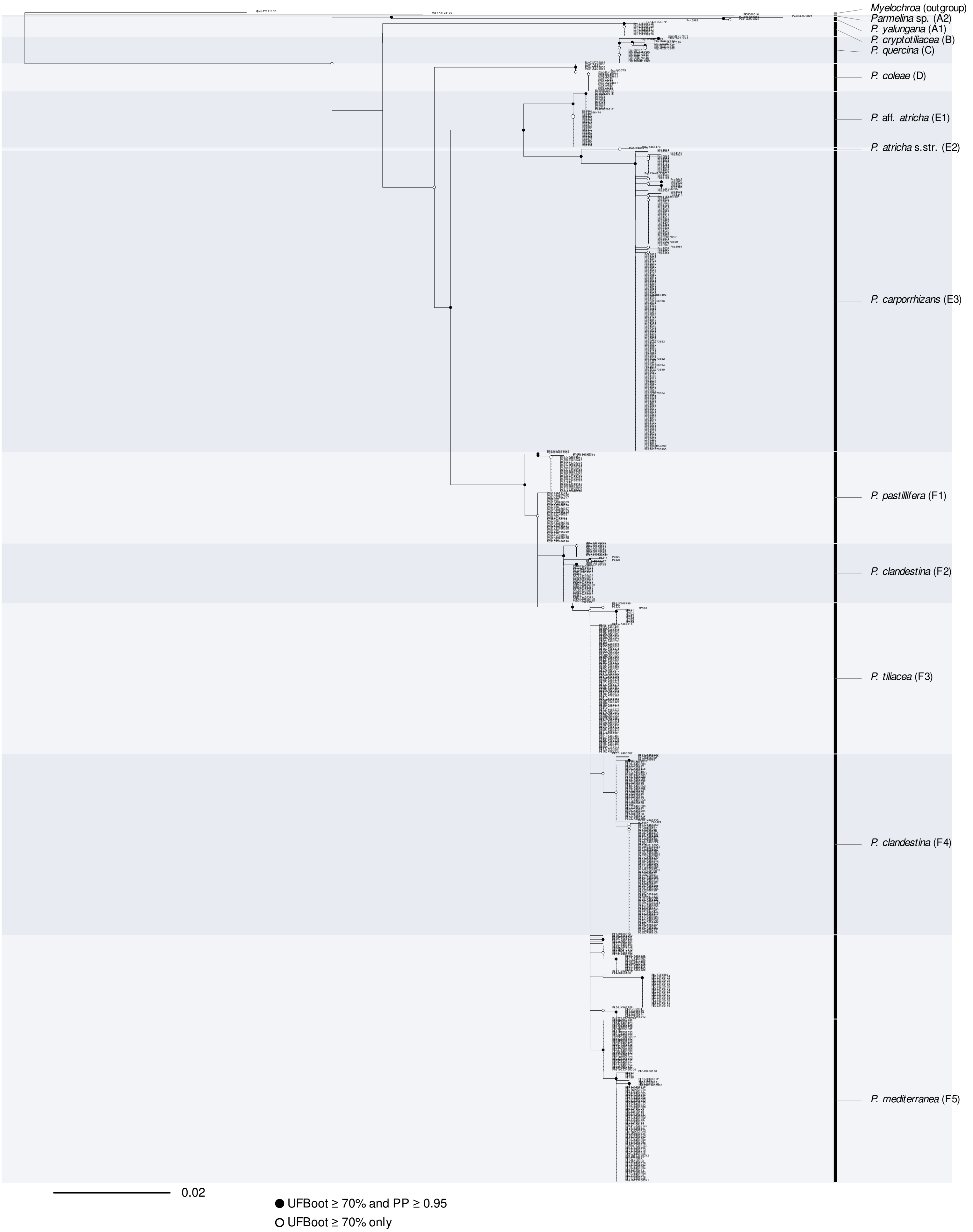

Table S1

Table S1. Specimens of *Parmelina* (and *Myelochroa*) species used in the present study, including collection data and GenBank accession numbers.

Sequence code	Species (this study)	Clade/subclade	Haplotype representative	Locality	GenBank	Newly generated	Haplotype in Figure 1
Pat1JX466473	*P. atricha*	E2	Pat1JX466473	France: Pyrénées-Orientales, La Preste, road towards le Fourquets	JX466473	no	yes
Pat2JX466474	*P. atricha*	E1	Pat2JX466474	Spain: Gerona, Espinavell, forest road towards Setcases	JX466474	no	yes
Pat3JX466475	*P. atricha*	E2	Pat3JX466475	France: Pyrénées-Orientales, La Preste, before the village	JX466475	no	yes
Pat4EU520312	*P. atricha*	E1	Pat4EU520312	France: Hautes-Pyrénées, Pyrénées, road from Andorra to Foix, near Mérens-les-Vals, past the Andorra customs	EU520312	no	yes
Pat5EU520313	*P. atricha*	E1	Pat4EU520312	France: Hautes-Pyrénées, Pyrénées, road from Andorra to Foix, near Mérens-les-Vals, past the Andorra customs	EU520313	no	no
Pat6EU520314	*P. atricha*	E1	Pat4EU520312	France: Hautes-Pyrénées, Pyrénées, road from Andorra to Foix, near Mérens-les-Vals, past the Andorra customs	EU520314	no	no
Pat7EU520315	*P. atricha*	E1	Pat4EU520312	France: Hautes-Pyrénées, Pyrénées, road from Andorra to Foix, near Mérens-les-Vals, past the Andorra customs	EU520315	no	no
Pat7330	*P. atricha*	E1	Pat4EU520312	Switzerland: Collonges, Plex, Plex parking	PZ290771	yes	no
Pat7331	*P. atricha*	E1	Pat4EU520312	Switzerland: Collonges, Plex, Plex parking	PZ290772	yes	no
Pat7370	*P. atricha*	E1	Pat4EU520312	Switzerland: Bourg-Saint-Pierre, scenic viewpoint (bench)	PZ290773	yes	no
Pat7374	*P. atricha*	E1	Pat4EU520312	Switzerland: Bourg-Saint-Pierre, scenic viewpoint (bench)	PZ290774	yes	no
Pat7384	*P. atricha*	E1	Pat4EU520312	Switzerland: Bourg-Saint-Pierre, scenic viewpoint (bench)	PZ290775	yes	no
Pat7397	*P. atricha*	E1	Pat4EU520312	Switzerland: Bourg-Saint-Pierre, scenic viewpoint (bench)	PZ290776	yes	no
Pat7401	*P. atricha*	E1	Pat4EU520312	Switzerland: Collonges, Plex, Plex parking	PZ290777	yes	no
Pat7403	*P. atricha*	E1	Pat4EU520312	Switzerland: Bourg-Saint-Pierre, scenic viewpoint (bench)	PZ290778	yes	no
Pat7406	*P. atricha*	E1	Pat4EU520312	Switzerland: Bourg-Saint-Pierre, Forêt des Trones	PZ290779	yes	no
Pat7346	*P. atricha*	E1	Pat7346	Switzerland: Collonges, Plex, Plex parking	PZ290780	yes	yes
Pat7359	*P. atricha*	E1	Pat7359	Switzerland: Bourg-Saint-Pierre, Forêt des Trones	PZ290781	yes	yes
Pat7336	*P. atricha*	E1	Pat7359	Switzerland: Collonges, Plex, Plex parking	PZ290782	yes	no
Pat7337	*P. atricha*	E1	Pat7359	Switzerland: Bourg-Saint-Pierre, Forêt des Trones	PZ290783	yes	no
Pat7338	*P. atricha*	E1	Pat7359	Switzerland: Bourg-Saint-Pierre, scenic viewpoint (bench)	PZ290784	yes	no
Pat7347	*P. atricha*	E1	Pat7359	Switzerland: Bourg-Saint-Pierre, Forêt des Trones	PZ290785	yes	no
Pat7348	*P. atricha*	E1	Pat7359	Switzerlan: Collonges, Plex, Plex parking	PZ290786	yes	no
Pat7369	*P. atricha*	E1	Pat7359	Switzerland: Collonges, Plex, Plex parking	PZ290787	yes	no
Pat7371	*P. atricha*	E1	Pat7359	Switzerland: Bourg-Saint-Pierre, Forêt des Trones	PZ290788	yes	no
Pat7372	*P. atricha*	E1	Pat7359	Switzerland: Collonges, Plex, Plex parking	PZ290789	yes	no
Pat7373	*P. atricha*	E1	Pat7359	Switzerland: Collonges, Plex, Plex parking	PZ290790	yes	no
Pat7375	*P. atricha*	E1	Pat7359	Switzerland: Collonges, Plex, Plex parking	PZ290791	yes	no
Pat7376	*P. atricha*	E1	Pat7359	Switzerland: Bourg-Saint-Pierre, scenic viewpoint (bench)	PZ290792	yes	no
Pat7377	*P. atricha*	E1	Pat7359	Switzerland: Bourg-Saint-Pierre, Forêt des Trones	PZ290793	yes	no
Pat7388	*P. atricha*	E1	Pat7359	Switzerland: Bourg-Saint-Pierre, Forêt des Trones	PZ290794	yes	no
Pat7389	*P. atricha*	E1	Pat7359	Switzerland: Collonges, Plex, Plex parking	PZ290795	yes	no
Pat7390	*P. atricha*	E1	Pat7359	Switzerland: Collonges, Plex, Plex parking	PZ290796	yes	no
Pat7393	*P. atricha*	E1	Pat7359	Switzerland: Collonges, Plex, Plex parking	PZ290797	yes	no
Pat7394	*P. atricha*	E1	Pat7359	Switzerland: Collonges, Plex, Plex parking	PZ290798	yes	no

Sequence code	Species (this study)	Clade/subclade	Haplotype representative	Locality	GenBank	Newly generated	Haplotype in Figure 1
Pat7395	*P. atricha*	E1	Pat7359	Switzerland: Collonges, Plex, Plex parking	PZ290799	yes	no
Pat7400	*P. atricha*	E1	Pat7359	Switzerland: Collonges, Plex, Plex parking	PZ290800	yes	no
Pat7402	*P. atricha*	E1	Pat7359	Switzerland: Bourg-Saint-Pierre, Forêt des Trones	PZ290801	yes	no
Pca2DQ273850	*P. carporrhizans*	E3	Pca2DQ273850	Spain: Castellón, Herbés, Puerto de la Creu	DQ273850	no	yes
Pca3DQ273851	*P. carporrhizans*	E3	Pca2DQ273850	Spain: Castellón, Herbés, Puerto de la Creu	DQ273851	no	no
Pca11KX457684	*P. carporrhizans*	E3	Pca2DQ273850	Armenia: Prov. of Tavush, Dilijan, Dilijan National Park, H50 road to Haghartsin Monastery	KX457684	no	no
Pca3350	*P. carporrhizans*	E3	Pca2DQ273850	Spain: Sierra de Gredos, Puerto del Pico	PZ290802	yes	no
Pca3365	*P. carporrhizans*	E3	Pca2DQ273850	Spain: Sierra de Gredos, Puerto del Pico	PZ290803	yes	no
Pca3419	*P. carporrhizans*	E3	Pca2DQ273850	Spain: Sierra de Gredos, Cuevas del Valle	PZ290804	yes	no
Pca3421	*P. carporrhizans*	E3	Pca2DQ273850	Spain: Sierra de Gredos, Cuevas del Valle	PZ290805	yes	no
Pca3426	*P. carporrhizans*	E3	Pca2DQ273850	Spain: Sierra de Gredos, Cuevas del Valle	PZ290806	yes	no
Pca3435	*P. carporrhizans*	E3	Pca2DQ273850	Spain: Sierra de Gredos, Cuevas del Valle	PZ290807	yes	no
Pca3646	*P. carporrhizans*	E3	Pca2DQ273850	Spain: Gran Canaria, Mirador de Degollada de Becerra	PZ290808	yes	no
Pca3655	*P. carporrhizans*	E3	Pca2DQ273850	Spain: Gran Canaria, Mirador de Degollada de Becerra	PZ290809	yes	no
Pca4015	*P. carporrhizans*	E3	Pca2DQ273850	Italy: Sicilia, San Fratello	PZ290810	yes	no
Pca4061	*P. carporrhizans*	E3	Pca2DQ273850	Italy: Sicilia, Monreale	PZ290811	yes	no
Pca4123	*P. carporrhizans*	E3	Pca2DQ273850	Morocco: near Beni Hassan, between Chefchaouen and Tetouan	PZ290812	yes	no
Pca4417	*P. carporrhizans*	E3	Pca2DQ273850	Spain: Castellón, Herbés	PZ290813	yes	no
Pca4493	*P. carporrhizans*	E3	Pca2DQ273850	Portugal: Alto Alentejo, Marvão	PZ290814	yes	no
Pca4495	*P. carporrhizans*	E3	Pca2DQ273850	Portugal: Alto Alentejo, Marvão	PZ290815	yes	no
Pca4514	*P. carporrhizans*	E3	Pca2DQ273850	Portugal: Beira Baixa, Covilhã	PZ290816	yes	no
Pca4517	*P. carporrhizans*	E3	Pca2DQ273850	Portugal: Beira Baixa, Covilhã	PZ290817	yes	no
Pca4518	*P. carporrhizans*	E3	Pca2DQ273850	Portugal: Beira Baixa, Covilhã	PZ290818	yes	no
Pca4519	*P. carporrhizans*	E3	Pca2DQ273850	Portugal: Beira Baixa, Covilhã	PZ290819	yes	no
Pca4522	*P. carporrhizans*	E3	Pca2DQ273850	Portugal: Beira Baixa, Covilhã	PZ290820	yes	no
Pca4526	*P. carporrhizans*	E3	Pca2DQ273850	Portugal: Beira Baixa, Covilhã	PZ290821	yes	no
Pca4532	*P. carporrhizans*	E3	Pca2DQ273850	Portugal: Beira Baixa, Famalicão	PZ290822	yes	no
Pca4536	*P. carporrhizans*	E3	Pca2DQ273850	Portugal: Beira Baixa, Famalicão	PZ290823	yes	no
Pca4537	*P. carporrhizans*	E3	Pca2DQ273850	Portugal: Beira Baixa, Famalicão	PZ290824	yes	no
Pca4538	*P. carporrhizans*	E3	Pca2DQ273850	Portugal: Beira Baixa, Famalicão	PZ290825	yes	no
Pca4543	*P. carporrhizans*	E3	Pca2DQ273850	Portugal: Beira Baixa, Famalicão	PZ290826	yes	no
Pca4545	*P. carporrhizans*	E3	Pca2DQ273850	Portugal: Beira Baixa, Famalicão	PZ290827	yes	no
Pca4549	*P. carporrhizans*	E3	Pca2DQ273850	Portugal: Beira Baixa, Famalicão	PZ290828	yes	no
Pca7JF756995	*P. carporrhizans*	E3	Pca7JF756995	Spain: Gran Canaria, Mirador de Degollada de Becerra	JF756995	no	yes
Pca10JF756993	*P. carporrhizans*	E3	Pca10JF756993	Turkey: Prov. of Bursa, Uludaj roadside	JF756993	no	yes
Pca1DQ273849	*P. carporrhizans*	E3	Pca10JF756993	Spain: Islas Canarias, Tenerife, El Sauzal, Ravelo	DQ273849	no	no
Pca4DQ273852	*P. carporrhizans*	E3	Pca10JF756993	Portugal: Fortaleza de Marvão	DQ273852	no	no
Pca5DQ273853	*P. carporrhizans*	E3	Pca10JF756993	Portugal: Fortaleza de Marvão	DQ273853	no	no
Pca6DQ273854	*P. carporrhizans*	E3	Pca10JF756993	Spain: Guadalajara, Selas	DQ273854	no	no
Pca8JF756996	*P. carporrhizans*	E3	Pca10JF756993	Spain: Islas Canarias, Tenerife, El Sauzal	JF756996	no	no
Pca9JF756994	*P. carporrhizans*	E3	Pca10JF756993	Turkey: Prov. of Canakkale, north of Karadag	JF756994	no	no
Pca12KM357893	*P. carporrhizans*	E3	Pca10JF756993	Spain: Sierra de Gredos, Cuevas del Valle	KM357893	no	no

Sequence code	Species (this study)	Clade/subclade	Haplotype representative	Locality	GenBank	Newly generated	Haplotype in Figure 1
Pca13KM357892	*P. carporrhizans*	E3	Pca10JF756993	Spain: Sierra de Gredos, Cuevas del Valle	KM357892	no	no
Pca3367	*P. carporrhizans*	E3	Pca10JF756993	Spain: Sierra de Gredos, Puerto del Pico	PZ290829	yes	no
Pca3418	*P. carporrhizans*	E3	Pca10JF756993	Spain: Sierra de Gredos, Cuevas del Valle	PZ290830	yes	no
Pca3423	*P. carporrhizans*	E3	Pca10JF756993	Spain: Sierra de Gredos, Cuevas del Valle	PZ290831	yes	no
Pca3425	*P. carporrhizans*	E3	Pca10JF756993	Spain: Sierra de Gredos, Cuevas del Valle	PZ290832	yes	no
Pca3427	*P. carporrhizans*	E3	Pca10JF756993	Spain: Sierra de Gredos, Cuevas del Valle	PZ290833	yes	no
Pca3428	*P. carporrhizans*	E3	Pca10JF756993	Spain: Sierra de Gredos, Cuevas del Valle	PZ290834	yes	no
Pca3429	*P. carporrhizans*	E3	Pca10JF756993	Spain: Sierra de Gredos, Cuevas del Valle	PZ290835	yes	no
Pca3430	*P. carporrhizans*	E3	Pca10JF756993	Spain: Sierra de Gredos, Cuevas del Valle	PZ290836	yes	no
Pca3431	*P. carporrhizans*	E3	Pca10JF756993	Spain: Sierra de Gredos, Cuevas del Valle	PZ290837	yes	no
Pca3432	*P. carporrhizans*	E3	Pca10JF756993	Spain: Sierra de Gredos, Cuevas del Valle	PZ290838	yes	no
Pca3433	*P. carporrhizans*	E3	Pca10JF756993	Spain: Sierra de Gredos, Cuevas del Valle	PZ290839	yes	no
Pca3434	*P. carporrhizans*	E3	Pca10JF756993	Spain: Sierra de Gredos, Cuevas del Valle	PZ290840	yes	no
Pca3437	*P. carporrhizans*	E3	Pca10JF756993	Spain: Sierra de Gredos, Cuevas del Valle	PZ290841	yes	no
Pca3618	*P. carporrhizans*	E3	Pca10JF756993	Spain: Gran Canaria, San Mateo	PZ290842	yes	no
Pca3619	*P. carporrhizans*	E3	Pca10JF756993	Spain: Gran Canaria, San Mateo	PZ290843	yes	no
Pca3620	*P. carporrhizans*	E3	Pca10JF756993	Spain: Gran Canaria, San Mateo	PZ290844	yes	no
Pca3621	*P. carporrhizans*	E3	Pca10JF756993	Spain: Gran Canaria, San Mateo	PZ290845	yes	no
Pca3622	*P. carporrhizans*	E3	Pca10JF756993	Spain: Gran Canaria, San Mateo	PZ290846	yes	no
Pca3623	*P. carporrhizans*	E3	Pca10JF756993	Spain: Gran Canaria, San Mateo	PZ290847	yes	no
Pca3624	*P. carporrhizans*	E3	Pca10JF756993	Spain: Gran Canaria, San Mateo	PZ290848	yes	no
Pca3625	*P. carporrhizans*	E3	Pca10JF756993	Spain: Gran Canaria, San Mateo	PZ290849	yes	no
Pca3626	*P. carporrhizans*	E3	Pca10JF756993	Spain: Gran Canaria, San Mateo	PZ290850	yes	no
Pca3627	*P. carporrhizans*	E3	Pca10JF756993	Spain: Gran Canaria, San Mateo	PZ290851	yes	no
Pca3629	*P. carporrhizans*	E3	Pca10JF756993	Spain: Gran Canaria, San Mateo	PZ290852	yes	no
Pca3630	*P. carporrhizans*	E3	Pca10JF756993	Spain: Gran Canaria, San Mateo	PZ290853	yes	no
Pca3631	*P. carporrhizans*	E3	Pca10JF756993	Spain: Gran Canaria, San Mateo	PZ290854	yes	no
Pca3636	*P. carporrhizans*	E3	Pca10JF756993	Spain: Gran Canaria, San Mateo	PZ290855	yes	no
Pca3637	*P. carporrhizans*	E3	Pca10JF756993	Spain: Gran Canaria, San Mateo	PZ290856	yes	no
Pca3638	*P. carporrhizans*	E3	Pca10JF756993	Spain: Gran Canaria, San Mateo	PZ290857	yes	no
Pca3642	*P. carporrhizans*	E3	Pca10JF756993	Spain: Gran Canaria, San Mateo	PZ290858	yes	no
Pca3645	*P. carporrhizans*	E3	Pca10JF756993	Spain: Gran Canaria, Mirador de Degollada de Becerra	PZ290859	yes	no
Pca3647	*P. carporrhizans*	E3	Pca10JF756993	Spain: Gran Canaria, Mirador de Degollada de Becerra	PZ290860	yes	no
Pca3651	*P. carporrhizans*	E3	Pca10JF756993	Spain: Gran Canaria, Mirador de Degollada de Becerra	PZ290861	yes	no
Pca3652	*P. carporrhizans*	E3	Pca10JF756993	Spain: Gran Canaria, Mirador de Degollada de Becerra	PZ290862	yes	no
Pca3654	*P. carporrhizans*	E3	Pca10JF756993	Spain: Gran Canaria, Mirador de Degollada de Becerra	PZ290863	yes	no
Pca3656	*P. carporrhizans*	E3	Pca10JF756993	Spain: Gran Canaria, Mirador de Degollada de Becerra	PZ290864	yes	no
Pca3657	*P. carporrhizans*	E3	Pca10JF756993	Spain: Gran Canaria, Mirador de Degollada de Becerra	PZ290865	yes	no
Pca3658	*P. carporrhizans*	E3	Pca10JF756993	Spain: Gran Canaria, Mirador de Degollada de Becerra	PZ290866	yes	no
Pca3659	*P. carporrhizans*	E3	Pca10JF756993	Spain: Gran Canaria, Mirador de Degollada de Becerra	PZ290867	yes	no
Pca3660	*P. carporrhizans*	E3	Pca10JF756993	Spain: Gran Canaria, Mirador de Degollada de Becerra	PZ290868	yes	no
Pca3661	*P. carporrhizans*	E3	Pca10JF756993	Spain: Gran Canaria, Mirador de Degollada de Becerra	PZ290869	yes	no

Sequence code	Species (this study)	Clade/subclade	Haplotype representative	Locality	GenBank	Newly generated	Haplotype in Figure 1
Pca3662	*P. carporrhizans*	E3	Pca10JF756993	Spain: Gran Canaria, Mirador de Degollada de Becerra	PZ290870	yes	no
Pca3663	*P. carporrhizans*	E3	Pca10JF756993	Spain: Gran Canaria, Mirador de Degollada de Becerra	PZ290871	yes	no
Pca3664	*P. carporrhizans*	E3	Pca10JF756993	Spain: Gran Canaria, Mirador de Degollada de Becerra	PZ290872	yes	no
Pca3668	*P. carporrhizans*	E3	Pca10JF756993	Spain: Gran Canaria, San Mateo	PZ290873	yes	no
Pca3983	*P. carporrhizans*	E3	Pca10JF756993	Italy: Sicilia, San Fratello	PZ290874	yes	no
Pca4011	*P. carporrhizans*	E3	Pca10JF756993	Italy: Sicilia, Capizzi	PZ290875	yes	no
Pca4012	*P. carporrhizans*	E3	Pca10JF756993	Italy: Sicilia, Capizzi	PZ290876	yes	no
Pca4013	*P. carporrhizans*	E3	Pca10JF756993	Italy: Sicilia, Capizzi	PZ290877	yes	no
Pca4014	*P. carporrhizans*	E3	Pca10JF756993	Italy: Sicilia, Capizzi	PZ290878	yes	no
Pca4016	*P. carporrhizans*	E3	Pca10JF756993	Italy: Sicilia, Capizzi	PZ290879	yes	no
Pca4053	*P. carporrhizans*	E3	Pca10JF756993	Italy: Sicilia, Monreale	PZ290880	yes	no
Pca4055	*P. carporrhizans*	E3	Pca10JF756993	Italy: Sicilia, Monreale	PZ290881	yes	no
Pca4056	*P. carporrhizans*	E3	Pca10JF756993	Italy: Sicilia, Monreale	PZ290882	yes	no
Pca4057	*P. carporrhizans*	E3	Pca10JF756993	Italy: Sicilia, Monreale	PZ290883	yes	no
Pca4058	*P. carporrhizans*	E3	Pca10JF756993	Italy: Sicilia, Monreale	PZ290884	yes	no
Pca4059	*P. carporrhizans*	E3	Pca10JF756993	Italy: Sicilia, Monreale	PZ290885	yes	no
Pca4060	*P. carporrhizans*	E3	Pca10JF756993	Italy: Sicilia, Monreale	PZ290886	yes	no
Pca4062	*P. carporrhizans*	E3	Pca10JF756993	Italy: Sicilia, Monreale	PZ290887	yes	no
Pca4063	*P. carporrhizans*	E3	Pca10JF756993	Italy: Sicilia, Monreale	PZ290888	yes	no
Pca4100	*P. carporrhizans*	E3	Pca10JF756993	Morocco: Tánger-Tetouán	PZ290889	yes	no
Pca4102	*P. carporrhizans*	E3	Pca10JF756993	Morocco: Tánger-Tetouán	PZ290890	yes	no
Pca4103	*P. carporrhizans*	E3	Pca10JF756993	Morocco: Tánger-Tetouán	PZ290891	yes	no
Pca4105	*P. carporrhizans*	E3	Pca10JF756993	Morocco: Tánger-Tetouán	PZ290892	yes	no
Pca4106	*P. carporrhizans*	E3	Pca10JF756993	Morocco: Tánger-Tetouán	PZ290893	yes	no
Pca4108	*P. carporrhizans*	E3	Pca10JF756993	Morocco: near Beni Hassan, between Chefchaouen and Tetouan	PZ290894	yes	no
Pca4111	*P. carporrhizans*	E3	Pca10JF756993	Morocco: near Beni Hassan, between Chefchaouen and Tetouan	PZ290895	yes	no
Pca4114	*P. carporrhizans*	E3	Pca10JF756993	Morocco: near Beni Hassan, between Chefchaouen and Tetouan	PZ290896	yes	no
Pca4118	*P. carporrhizans*	E3	Pca10JF756993	Morocco: near Beni Hassan, between Chefchaouen and Tetouan	PZ290897	yes	no
Pca4120	*P. carporrhizans*	E3	Pca10JF756993	Morocco: near Beni Hassan, between Chefchaouen and Tetouan	PZ290898	yes	no
Pca4122	*P. carporrhizans*	E3	Pca10JF756993	Morocco: near Beni Hassan, between Chefchaouen and Tetouan	PZ290899	yes	no
Pca4384	*P. carporrhizans*	E3	Pca10JF756993	Spain: Cádiz, Grazalema	PZ290900	yes	no
Pca4385	*P. carporrhizans*	E3	Pca10JF756993	Spain: Cádiz, Grazalema	PZ290901	yes	no
Pca4387	*P. carporrhizans*	E3	Pca10JF756993	Spain: Cádiz, Grazalema	PZ290902	yes	no
Pca4388	*P. carporrhizans*	E3	Pca10JF756993	Spain: Cádiz, Grazalema	PZ290903	yes	no
Pca4389	*P. carporrhizans*	E3	Pca10JF756993	Spain: Cádiz, Grazalema	PZ290904	yes	no
Pca4392	*P. carporrhizans*	E3	Pca10JF756993	Spain: Cádiz, Grazalema	PZ290905	yes	no
Pca4399	*P. carporrhizans*	E3	Pca10JF756993	Spain: Castellón, Herbés	PZ290906	yes	no
Pca4401	*P. carporrhizans*	E3	Pca10JF756993	Spain: Castellón, Herbés	PZ290907	yes	no
Pca4405	*P. carporrhizans*	E3	Pca10JF756993	Spain: Castellón, Herbés	PZ290908	yes	no
Pca4406	*P. carporrhizans*	E3	Pca10JF756993	Spain: Castellón, Herbés	PZ290909	yes	no
Pca4421	*P. carporrhizans*	E3	Pca10JF756993	Spain: Cádiz, Grazalema	PZ290910	yes	no
Pca4494	*P. carporrhizans*	E3	Pca10JF756993	Portugal: Alto Alentejo, Marvão	PZ290911	yes	no

Sequence code	Species (this study)	Clade/subclade	Haplotype representative	Locality	GenBank	Newly generated	Haplotype in Figure 1
Pca4496	*P. carporrhizans*	E3	Pca10JF756993	Portugal: Alto Alentejo, Marvão	PZ290912	yes	no
Pca4497	*P. carporrhizans*	E3	Pca10JF756993	Portugal: Alto Alentejo, Marvão	PZ290913	yes	no
Pca4498	*P. carporrhizans*	E3	Pca10JF756993	Portugal: Alto Alentejo, Marvão	PZ290914	yes	no
Pca4502	*P. carporrhizans*	E3	Pca10JF756993	Portugal: Alto Alentejo, Marvão	PZ290915	yes	no
Pca4503	*P. carporrhizans*	E3	Pca10JF756993	Portugal, Alto Alentejo, Marvão	PZ290916	yes	no
Pca4507	*P. carporrhizans*	E3	Pca10JF756993	Portugal: Alto Alentejo, Marvão	PZ290917	yes	no
Pca4508	*P. carporrhizans*	E3	Pca10JF756993	Portugal: Alto Alentejo, Marvão	PZ290918	yes	no
Pca4509	*P. carporrhizans*	E3	Pca10JF756993	Portugal: Alto Alentejo, Marvão	PZ290919	yes	no
Pca4511	*P. carporrhizans*	E3	Pca10JF756993	Portugal: Alto Alentejo, Marvão	PZ290920	yes	no
Pca4513	*P. carporrhizans*	E3	Pca10JF756993	Portugal: Beira Baixa, Covilhã	PZ290921	yes	no
Pca4515	*P. carporrhizans*	E3	Pca10JF756993	Portugal: Beira Baixa, Covilhã	PZ290922	yes	no
Pca4516	*P. carporrhizans*	E3	Pca10JF756993	Portugal: Beira Baixa, Covilhã	PZ290923	yes	no
Pca4521	*P. carporrhizans*	E3	Pca10JF756993	Portugal: Beira Baixa, Covilhã	PZ290924	yes	no
Pca4523	*P. carporrhizans*	E3	Pca10JF756993	Portugal: Beira Baixa, Covilhã	PZ290925	yes	no
Pca4524	*P. carporrhizans*	E3	Pca10JF756993	Portugal: Beira Baixa, Covilhã	PZ290926	yes	no
Pca4528	*P. carporrhizans*	E3	Pca10JF756993	Portugal: Beira Baixa, Covilhã	PZ290927	yes	no
Pca4529	*P. carporrhizans*	E3	Pca10JF756993	Portugal: Beira Baixa, Covilhã	PZ290928	yes	no
Pca4530	*P. carporrhizans*	E3	Pca10JF756993	Portugal: Beira Baixa, Famalicão	PZ290929	yes	no
Pca4531	*P. carporrhizans*	E3	Pca10JF756993	Portugal: Beira Baixa, Famalicão	PZ290930	yes	no
Pca4535	*P. carporrhizans*	E3	Pca10JF756993	Portugal: Beira Baixa, Famalicão	PZ290931	yes	no
Pca4539	*P. carporrhizans*	E3	Pca10JF756993	Portugal: Beira Baixa, Famalicão	PZ290932	yes	no
Pca4540	*P. carporrhizans*	E3	Pca10JF756993	Portugal: Beira Baixa, Famalicão	PZ290933	yes	no
Pca4541	*P. carporrhizans*	E3	Pca10JF756993	Portugal: Beira Baixa, Famalicão	PZ290934	yes	no
Pca4542	*P. carporrhizans*	E3	Pca10JF756993	Portugal: Beira Baixa, Famalicão	PZ290935	yes	no
Pca4546	*P. carporrhizans*	E3	Pca10JF756993	Portugal: Beira Baixa, Famalicão	PZ290936	yes	no
Pca4547	*P. carporrhizans*	E3	Pca10JF756993	Portugal: Beira Baixa, Famalicão	PZ290937	yes	no
Pca4548	*P. carporrhizans*	E3	Pca10JF756993	Portugal: Beira Baixa, Famalicão	PZ290938	yes	no
Pca4551	*P. carporrhizans*	E3	Pca10JF756993	Portugal: Beira Baixa, Famalicão	PZ290939	yes	no
Pca4552	*P. carporrhizans*	E3	Pca10JF756993	Portugal: Beira Baixa, Famalicão	PZ290940	yes	no
Pca4554	*P. carporrhizans*	E3	Pca10JF756993	Portugal: Beira Baixa, Famalicão	PZ290941	yes	no
Pca4556	*P. carporrhizans*	E3	Pca10JF756993	Portugal: Beira Baixa, Famalicão	PZ290942	yes	no
Pca4557	*P. carporrhizans*	E3	Pca10JF756993	Portugal: Beira Baixa, Famalicão	PZ290943	yes	no
Pca4558	*P. carporrhizans*	E3	Pca10JF756993	Portugal: Beira Baixa, Famalicão	PZ290944	yes	no
Pca4559	*P. carporrhizans*	E3	Pca10JF756993	Portugal: Beira Baixa, Famalicão	PZ290945	yes	no
Pca3364	*P. carporrhizans*	E3	Pca3364	Spain: Sierra de Gredos, Puerto del Pico	PZ290946	yes	yes
Pca3639	*P. carporrhizans*	E3	Pca3364	Spain: Gran Canaria, San Mateo	PZ290947	yes	no
Pca4525	*P. carporrhizans*	E3	Pca3364	Portugal: Beira Baixa, Covilhã	PZ290948	yes	no
Pca4550	*P. carporrhizans*	E3	Pca3364	Portugal: Beira Baixa, Famalicão	PZ290949	yes	no
Pca3366	*P. carporrhizans*	E3	Pca3366	Spain: Sierra de Gredos, Puerto del Pico	PZ290950	yes	yes
Pca4390	*P. carporrhizans*	E3	Pca3366	Spain: Cádiz, Grazalema	PZ290951	yes	no
Pca4555	*P. carporrhizans*	E3	Pca3366	Portugal: Beira Baixa, Famalicão	PZ290952	yes	no
Pca3424	*P. carporrhizans*	E3	Pca3424	Spain: Sierra de Gredos, Puerto del Pico	PZ290953	yes	yes

Sequence code	Species (this study)	Clade/subclade	Haplotype representative	Locality	GenBank	Newly generated	Haplotype in Figure 1
Pca3436	*P. carporrhizans*	E3	Pca3436	Spain: Sierra de Gredos, Puerto del Pico	PZ290954	yes	yes
Pca3641	*P. carporrhizans*	E3	Pca3436	Spain: Gran Canaria, San Mateo	PZ290955	yes	no
Pca3653	*P. carporrhizans*	E3	Pca3436	Spain: Gran Canaria, Mirador de Degollada de Becerra	PZ290956	yes	no
Pca4064	*P. carporrhizans*	E3	Pca3436	Italy: Sicilia, Monreale	PZ290957	yes	no
Pca4107	*P. carporrhizans*	E3	Pca3436	Morocco: Tánger-Tetouán	PZ290958	yes	no
Pca4110	*P. carporrhizans*	E3	Pca3436	Morocco: Tánger-Tetouán	PZ290959	yes	no
Pca4400	*P. carporrhizans*	E3	Pca3436	Spain: Castellón, Herbés	PZ290960	yes	no
Pca4492	*P. carporrhizans*	E3	Pca3436	Portugal: Alto Alentejo, Marvão	PZ290961	yes	no
Pca4504	*P. carporrhizans*	E3	Pca3436	Portugal: Alto Alentejo, Marvão	PZ290962	yes	no
Pca4512	*P. carporrhizans*	E3	Pca3436	Portugal: Alto Alentejo, Marvão	PZ290963	yes	no
Pca4533	*P. carporrhizans*	E3	Pca3436	Portugal: Beira Baixa, Famalicão	PZ290964	yes	no
Pca3628	*P. carporrhizans*	E3	Pca3628	Spain: Gran Canaria, San Mateo	PZ290965	yes	yes
Pca3648	*P. carporrhizans*	E3	Pca3628	Spain: Gran Canaria, Mirador de Degollada de Becerra	PZ290966	yes	no
Pca3984	*P. carporrhizans*	E3	Pca3984	Italy: Sicilia, San Fratello	PZ290967	yes	yes
Pca3985	*P. carporrhizans*	E3	Pca3985	Italy: Sicilia, San Fratello	PZ290968	yes	yes
Pca4054	*P. carporrhizans*	E3	Pca4054	Italy: Sicilia, Monreale	PZ290969	yes	yes
Pca4101	*P. carporrhizans*	E3	Pca4101	Morocco: near Beni Hassan, between Chefchaouen and Tetouan	PZ290970	yes	yes
Pca4104	*P. carporrhizans*	E3	Pca4101	Morocco: Tánger-Tetouán	PZ290971	yes	no
Pca4112	*P. carporrhizans*	E3	Pca4112	Morocco: near Beni Hassan, between Chefchaouen and Tetouan	PZ290972	yes	yes
Pca4119	*P. carporrhizans*	E3	Pca4119	Morocco: near Beni Hassan, between Chefchaouen and Tetouan	PZ290973	yes	yes
Pca4411	*P. carporrhizans*	E3	Pca4411	Spain: Castellón, Herbés	PZ290974	yes	yes
Pca4416	*P. carporrhizans*	E3	Pca4416	Spain: Castellón, Herbés	PZ290975	yes	yes
Pca4506	*P. carporrhizans*	E3	Pca4506	Portugal: Alto Alentejo, Marvão	PZ290976	yes	yes
Pca4520	*P. carporrhizans*	E3	Pca4520	Portugal: Beira Baixa, Covilhã	PZ290977	yes	yes
Pca4527	*P. carporrhizans*	E3	Pca4527	Portugal: Beira Baixa, Covilhã	PZ290978	yes	yes
Pca4534	*P. carporrhizans*	E3	Pca4534	Portugal: Beira Baixa, Famalicão	PZ290979	yes	yes
Pca4544	*P. carporrhizans*	E3	Pca4544	Portugal: Beira Baixa, Famalicão	PZ290980	yes	yes
Pqu13AY611105	*P. carporrhizans*	E3	Pqu13AY611105	Spain: Madrid, El Boalo, Mataelpino	AY611105	no	yes
Pat7396	*P. clandestina*	F4	Pat7396	Switzerland: Martigny, Fully, route des Follateres, about 1926 Fully	PZ290981	yes	yes
Pcl25JX466131	*P. clandestina*	F4	Pcl25JX466131	Spain: La Palma, road to Roque after the junction to Pico de la Nieve	JX466131	no	yes
Pat7385	*P. clandestina*	F4		Switzerland: Martigny, Fully, route des Follateres, about 1926 Fully	PZ290982	yes	no
Pcl288JX466445	*P. clandestina*	F4	Pcl25JX466131	United Kingdom: Wales, road A5, between Rhydlydan and Glasfryn	JX466445	no	no
Pcl289JX466446	*P. clandestina*	F4	Pcl25JX466131	United Kingdom: Wales, road A5, between Rhydlydan and Glasfryn	JX466446	no	no
Pcl323JX466439	*P. clandestina*	F4	Pcl25JX466131	Turkey: Eskisehir, Sivirihisar Mountains	JX466439	no	no
Pcl325JX466441	*P. clandestina*	F4	Pcl25JX466131	Turkey: Eskisehir, Sivirihisar Mountains	JX466441	no	no
Pti7JF756976	*P. clandestina*	F4	Pcl25JX466131	Morocco: Medium Atlas, Ifrane	JF756976	no	no
Pti13JF756992	*P. clandestina*	F4	Pcl25JX466131	Slovenia: Bled, east side of the lake	JF756992	no	no
Pti15JF756980	*P. clandestina*	F4	Pcl25JX466131	Spain: Madrid, El Escorial, surroundings of Silla de Felipe II	JF756980	no	no
Pti19AY581084	*P. clandestina*	F4	Pcl25JX466131	Spain: Teruel, Andorra	AY581084	no	no
Pti20DQ273861	*P. clandestina*	F4	Pcl25JX466131	Spain: Madrid, Robledillo de la Jara	DQ273861	no	no
Pti23JX466265	*P. clandestina*	F4	Pcl25JX466131	Spain: Madrid, Montejo de la Sierra, beech forest, next to the river	JX466265	no	no
Pti27JX466133	*P. clandestina*	F4	Pcl25JX466131	Spain: La Palma, Road to Roque after the junction to Pico de la Nieve	JX466133	no	no

Sequence code	Species (this study)	Clade/subclade	Haplotype representative	Locality	GenBank	Newly generated	Haplotype in Figure 1
Pti28JX466134	*P. clandestina*	F4	Pcl25JX466131	Spain: La Palma, Road to Roque after the junction to Pico de la Nieve	JX466134	no	no
Pti30JX466136	*P. clandestina*	F4	Pcl25JX466131	Spain: La Palma, Road to Roque after the junction to Pico de la Nieve	JX466136	no	no
Pti31JX466137	*P. clandestina*	F4	Pcl25JX466131	Spain: La Palma, Road to Roque after the junction to Pico de la Nieve	JX466137	no	no
Pti32JX466138	*P. clandestina*	F4	Pcl25JX466131	Spain: La Palma, Road to Roque after the junction to Pico de la Nieve	JX466138	no	no
Pti72JX466159	*P. clandestina*	F4	Pcl25JX466131	Spain: Gran Canaria, Degollada de Becerra	JX466159	no	no
Pti74JX466161	*P. clandestina*	F4	Pcl25JX466131	Spain: Gran Canaria, Degollada de Becerra	JX466161	no	no
Pti75JX466162	*P. clandestina*	F4	Pcl25JX466131	Spain: Gran Canaria, Degollada de Becerra	JX466162	no	no
Pti85JX466172	*P. clandestina*	F4	Pcl25JX466131	Spain: Gran Canaria, above Valleseco, in cross of San Mateo and Antenara GC-21 / GC-231	JX466172	no	no
Pti87JX466174	*P. clandestina*	F4	Pcl25JX466131	Spain: Gran Canaria, above Valleseco, in cross of San Mateo and Antenara GC-21 / GC-231	JX466174	no	no
Pti92JX466197	*P. clandestina*	F4	Pcl25JX466131	Morocco: Ifrane, Forêt Sidi	JX466197	no	no
Pti95JX466200	*P. clandestina*	F4	Pcl25JX466131	Morocco: Ifrane, Forêt Sidi	JX466200	no	no
Pti100JX466205	*P. clandestina*	F4	Pcl25JX466131	Morocco: Ifrane, Forêt Sidi	JX466205	no	no
Pti103JX466209	*P. clandestina*	F4	Pcl25JX466131	Spain: Cádiz, La Sauceda	JX466209	no	no
Pti108JX466214	*P. clandestina*	F4	Pcl25JX466131	Spain: Cádiz, La Sauceda	JX466214	no	no
Pti113JX466219	*P. clandestina*	F4	Pcl25JX466131	Spain: Cádiz, Grazalema	JX466219	no	no
Pti115JX466221	*P. clandestina*	F4	Pcl25JX466131	Spain: Cádiz, Grazalema	JX466221	no	no
Pti117JX466223	*P. clandestina*	F4	Pcl25JX466131	Spain: Cádiz, Grazalema	JX466223	no	no
Pti118JX466224	*P. clandestina*	F4	Pcl25JX466131	Spain: Cádiz, Grazalema	JX466224	no	no
Pti119JX466225	*P. clandestina*	F4	Pcl25JX466131	Spain: Cádiz, Grazalema	JX466225	no	no
Pti120JX466226	*P. clandestina*	F4	Pcl25JX466131	Spain: Cádiz, Grazalema	JX466226	no	no
Pti121JX466227	*P. clandestina*	F4	Pcl25JX466131	Spain: Cádiz, Grazalema	JX466227	no	no
Pti122JX466228	*P. clandestina*	F4	Pcl25JX466131	Spain: Ciudad Real, Aldea del Rey, Castillo de Calatrava	JX466228	no	no
Pti123JX466229	*P. clandestina*	F4	Pcl25JX466131	Spain: Ciudad Real, Aldea del Rey, Castillo de Calatrava	JX466229	no	no
Pti124JX466230	*P. clandestina*	F4	Pcl25JX466131	Spain: Ciudad Real, Aldea del Rey, Castillo de Calatrava	JX466230	no	no
Pti125JX466231	*P. clandestina*	F4	Pcl25JX466131	Spain: Ciudad Real, Minas de San Quintín	JX466231	no	no
Pti138JX466243	*P. clandestina*	F4	Pcl25JX466131	Spain: Cáceres, Cuacos de Yuste to Garganta de la Olla Road	JX466243	no	no
Pti139JX466244	*P. clandestina*	F4	Pcl25JX466131	Spain: Cáceres, Cuacos de Yuste to Garganta de la Olla Road	JX466244	no	no
Pti140JX466245	*P. clandestina*	F4	Pcl25JX466131	Spain: Cáceres, Cuacos de Yuste to Garganta de la Olla Road	JX466245	no	no
Pti141JX466246	*P. clandestina*	F4	Pcl25JX466131	Spain: Cáceres, Cuacos de Yuste to Garganta de la Olla Road	JX466246	no	no
Pti142JX466247	*P. clandestina*	F4	Pcl25JX466131	Spain: Salamanca, Peña de Francia	JX466247	no	no
Pti143JX466248	*P. clandestina*	F4	Pcl25JX466131	Spain: Salamanca, Peña de Francia	JX466248	no	no
Pti144JX466249	*P. clandestina*	F4	Pcl25JX466131	Spain: Salamanca, Peña de Francia	JX466249	no	no
Pti145JX466250	*P. clandestina*	F4	Pcl25JX466131	Spain: Salamanca, Peña de Francia	JX466250	no	no
Pti146JX466251	*P. clandestina*	F4	Pcl25JX466131	Spain: Salamanca, Morasverdes	JX466251	no	no
Pti147JX466252	*P. clandestina*	F4	Pcl25JX466131	Spain: Salamanca, Morasverdes	JX466252	no	no
Pti149JX466254	*P. clandestina*	F4	Pcl25JX466131	Spain: Salamanca, Morasverdes	JX466254	no	no
Pti152JX466260	*P. clandestina*	F4	Pcl25JX466131	Spain: Madrid, El Escorial, surroundings of Silla de Felipe II	JX466260	no	no
Pti157JX466259	*P. clandestina*	F4	Pcl25JX466131	Spain: Madrid, El Pardo	JX466259	no	no
Pti159JX466266	*P. clandestina*	F4	Pcl25JX466131	Spain: Madrid, Montejo de la Sierra, beech forest, next to the river	JX466266	no	no
Pti160JX466267	*P. clandestina*	F4	Pcl25JX466131	Spain: Madrid, Montejo de la Sierra, beech forest, next to the river	JX466267	no	no
Pti161JX466268	*P. clandestina*	F4	Pcl25JX466131	Spain: Madrid, Montejo de la Sierra, beech forest, next to the river	JX466268	no	no
Pti162JX466283	*P. clandestina*	F4	Pcl25JX466131	Spain: Asturias, Saliencia	JX466283	no	no

Sequence code	Species (this study)	Clade/subclade	Haplotype representative	Locality	GenBank	Newly generated	Haplotype in Figure 1
Pti163JX466284	*P. clandestina*	F4	Pcl25JX466131	Spain: Asturias, Saliencia	JX466284	no	no
Pti164JX466285	*P. clandestina*	F4	Pcl25JX466131	Spain: Asturias, Saliencia	JX466285	no	no
Pti165JX466286	*P. clandestina*	F4	Pcl25JX466131	Spain: Asturias, Saliencia	JX466286	no	no
Pti166JX466279	*P. clandestina*	F4	Pcl25JX466131	Spain: Cantabria, La Lomba, ascent to Alto Campoo station	JX466279	no	no
Pti167JX466280	*P. clandestina*	F4	Pcl25JX466131	Spain: Cantabria, La Lomba, ascent to Alto Campoo station	JX466280	no	no
Pti168JX466281	*P. clandestina*	F4	Pcl25JX466131	Spain: Cantabria, La Lomba, ascent to Alto Campoo station	JX466281	no	no
Pti238JX466323	*P. clandestina*	F4	Pcl25JX466131	France: Cordes, Château de Cordes	JX466323	no	no
Pti280JX466410	*P. clandestina*	F4	Pcl25JX466131	Germany: Bad Reichenhall	JX466410	no	no
Pti287JX466444	*P. clandestina*	F4	Pcl25JX466131	United Kingdom: Wales, road A5, between Rhydlydan and Glasfryn	JX466444	no	no
Pti311JX466425	*P. clandestina*	F4	Pcl25JX466131	Greece: Daphnes, Samotracia island	JX466425	no	no
Pti371MK812693	*P. clandestina*	F4	Pcl25JX466131	Norway: Vestfold, Tjome, Tjome church	MK812693	no	no
Pti375MK812323	*P. clandestina*	F4	Pcl25JX466131	Norway: Akershus, As, 200 m northwest of Norby church	MK812323	no	no
PtiI1954	*P. clandestina*	F4	Pcl25JX466131	Spain: Valencia, Aleppo pine forest in the north of the Prov. of Alicante	PZ290985	yes	no
PtiI1992	*P. clandestina*	F4	Pcl25JX466131	Spain: Valencia, Aleppo pine forest in the north of the Prov. of Alicante	PZ290986	yes	no
Pti7405	*P. clandestina*	F4	Pcl25JX466131	Spain: Segovia, Hayedo de la Pedrosa	PZ290983	yes	no
Pti7414	*P. clandestina*	F4	Pcl25JX466131	Switzerland: Collonges, Plex, Plex parking	PZ290984	yes	no
Pcl93JX466198	*P. clandestina*	F4	Pcl93JX466198	Morocco: Ifrane, Forêt Sidi	JX466198	no	yes
Pcl98JX466203	*P. clandestina*	F4	Pcl93JX466198	Morocco: Ifrane, Forêt Sidi	JX466203	no	no
Pcl99JX466204	*P. clandestina*	F4	Pcl93JX466198	Morocco: Ifrane, Forêt Sidi	JX466204	no	no
Pcl132JX466237	*P. clandestina*	F4	Pcl93JX466198	Spain: Cáceres, Parque Natural de Monfragüe	JX466237	no	no
Pcl285JX466417	*P. clandestina*	F4	Pcl93JX466198	Germany: Bavaria, Oberbayern, Kreis Meisbach, Bad Wiessee	JX466417	no	no
Pti5JF756979	*P. clandestina*	F4	Pcl93JX466198	Spain: Madrid, Madrid, El Pardo	JF756979	no	no
Pti9JF756978	*P. clandestina*	F4	Pcl93JX466198	India: Uttarahkand, Karnaprayag district, Badrinath, on way Mana to Vasundhara Fall	JF756978	no	no
Pti14JF756981	*P. clandestina*	F4	Pcl93JX466198	Portugal: Bragança, Gondesende, 8 km from Bragança capital, next to the town	JF756981	no	no
Pti17JF756988	*P. clandestina*	F4	Pcl93JX466198	Morocco: Medium Atlas, Ifrane, Forest de Yeiva	JF756988	no	no
Pti26JX466132	*P. clandestina*	F4	Pcl93JX466198	Spain: La Palma, road to Roque after the junction to Pico de la Nieve	JX466132	no	no
Pti29JX466135	*P. clandestina*	F4	Pcl93JX466198	Spain: La Palma, road to Roque after the junction to Pico de la Nieve	JX466135	no	no
Pti71JX466158	*P. clandestina*	F4	Pcl93JX466198	Spain: Gran Canaria, Degollada de Becerra	JX466158	no	no
Pti73JX466160	*P. clandestina*	F4	Pcl93JX466198	Spain: Gran Canaria, Degollada de Becerra	JX466160	no	no
Pti76JX466163	*P. clandestina*	F4	Pcl93JX466198	Spain: Gran Canaria, Degollada de Becerra	JX466163	no	no
Pti78JX466165	*P. clandestina*	F4	Pcl93JX466198	Spain: Gran Canaria, Degollada de Becerra	JX466165	no	no
Pti79JX466166	*P. clandestina*	F4	Pcl93JX466198	Spain: Gran Canaria, Degollada de Becerra	JX466166	no	no
Pti80JX466167	*P. clandestina*	F4	Pcl93JX466198	Spain: Gran Canaria, Degollada de Becerra	JX466167	no	no
Pti82JX466169	*P. clandestina*	F4	Pcl93JX466198	Spain: Gran Canaria, above Valleseco, in cross of San Mateo and Antenara GC-21 / GC-231	JX466169	no	no
Pti83JX466170	*P. clandestina*	F4	Pcl93JX466198	Spain: Gran Canaria, above Valleseco, in cross of San Mateo and Antenara GC-21 / GC-232	JX466170	no	no
Pti86JX466173	*P. clandestina*	F4	Pcl93JX466198	Spain: Gran Canaria, above Valleseco, in cross of San Mateo and Antenara GC-21 / GC-233	JX466173	no	no
Pti88JX466175	*P. clandestina*	F4	Pcl93JX466198	Spain: Gran Canaria, above Valleseco, in cross of San Mateo and Antenara GC-21 / GC-234	JX466175	no	no
Pti89JX466176	*P. clandestina*	F4	Pcl93JX466198	Spain: Gran Canaria, above Valleseco, in cross of San Mateo and Antenara GC-21 / GC-235	JX466176	no	no
Pti96JX466201	*P. clandestina*	F4	Pcl93JX466198	Morocco: Ifrane, Forêt Sidi	JX466201	no	no
Pti107JX466213	*P. clandestina*	F4	Pcl93JX466198	Spain: Cádiz, La Sauceda	JX466213	no	no
Pti114JX466220	*P. clandestina*	F4	Pcl93JX466198	Spain: Cádiz, Grazalema	JX466220	no	no
Pti126JX466232	*P. clandestina*	F4	Pcl93JX466198	Spain: Ciudad Real, Minas de San Quintín	JX466232	no	no

Sequence code	Species (this study)	Clade/subclade	Haplotype representative	Locality	GenBank	Newly generated	Haplotype in Figure 1
Pti127JX466256	*P. clandestina*	F4	Pcl93JX466198	Spain: Cáceres, 5 km south of Nvalvillar de Ibor, EX-118 road between km 18 and 19, entrance to the Alcornocosa estate	JX466256	no	no
Pti128JX466233	*P. clandestina*	F4	Pcl93JX466198	Spain: Cáceres, Parque Natural de Monfragüe	JX466233	no	no
Pti129JX466234	*P. clandestina*	F4	Pcl93JX466198	Spain: Cáceres, Parque Natural de Monfragüe	JX466234	no	no
Pti131JX466236	*P. clandestina*	F4	Pcl93JX466198	Spain: Cáceres, Parque Natural de Monfragüe	JX466236	no	no
Pti136JX466241	*P. clandestina*	F4	Pcl93JX466198	Spain: Cáceres, Monfragüe, way to the castle	JX466241	no	no
Pti148JX466253	*P. clandestina*	F4	Pcl93JX466198	Spain: Salamanca, Morasverdes	JX466253	no	no
Pti150JX466255	*P. clandestina*	F4	Pcl93JX466198	Spain: Salamanca, Morasverdes	JX466255	no	no
Pti158JX466264	*P. clandestina*	F4	Pcl93JX466198	Spain: Montejo de la Sierra, beech forest, next to the river	JX466264	no	no
Pti169JX466282	*P. clandestina*	F4	Pcl93JX466198	Spain: Cantabria, La Lomba, ascent to Alto Campoo station	JX466282	no	no
Pti277JX466407	*P. clandestina*	F4	Pcl93JX466198	Germany: Bad Reichenhall	JX466407	no	no
Pti305JX466418	*P. clandestina*	F4	Pcl93JX466198	Greece, Maries, Thasos island	JX466418	no	no
Pti7408	*P. clandestina*	F4	Pcl93JX466198	Switzerland: Martigny, Fully, route des Follateres, about 1926 Fully	PZ290987	yes	no
Pcl237JX466355	*P. clandestina*	F2	Pcl237JX466355	Italy: Toscana, Monte Amiatta	JX466355	no	yes
Pcl259JX466389	*P. clandestina*	F2	Pcl237JX466355	Slovenia: Bled, east side of the lake	JX466389	no	no
Pcl260JX466390	*P. clandestina*	F2	Pcl237JX466355	Slovenia: Bled, east side of the lake	JX466390	no	no
Pti180JX466287	*P. clandestina*	F2	Pcl237JX466355	Spain: Navarra, Belagua	JX466287	no	no
Pti209JX466360	*P. clandestina*	F2	Pcl237JX466355	Italy: Lodrino, Val Trompia	JX466360	no	no
Pti210JX466361	*P. clandestina*	F2	Pcl237JX466355	Italy: Lodrino, Val Trompia	JX466361	no	no
Pti252JX466380	*P. clandestina*	F2	Pcl237JX466355	Italy: Friuli-Venezia Giulia, Camporosso	JX466380	no	no
Pti255JX466383	*P. clandestina*	F2	Pcl237JX466355	Italy: Friuli-Venezia Giulia, Camporosso	JX466383	no	no
Pti256JX466384	*P. clandestina*	F2	Pcl237JX466355	Italy: Friuli-Venezia Giulia, Camporosso	JX466384	no	no
Pti261JX466391	*P. clandestina*	F2	Pcl237JX466355	Slovenia: Bled, east side of the lake	JX466391	no	no
Pti262JX466392	*P. clandestina*	F2	Pcl237JX466355	Slovenia: Bled, east side of the lake	JX466392	no	no
Pti263JX466393	*P. clandestina*	F2	Pcl237JX466355	Slovenia: Bled, east side of the lake	JX466393	no	no
Pti264JX466394	*P. clandestina*	F2	Pcl237JX466355	Slovenia: Bled, east side of the church	JX466394	no	no
Pti265JX466395	*P. clandestina*	F2	Pcl237JX466355	Slovenia: Bled, east side of the church	JX466395	no	no
Pti266JX466396	*P. clandestina*	F2	Pcl237JX466355	Slovenia: Bled, east side of the church	JX466396	no	no
Pti274JX466404	*P. clandestina*	F2	Pcl237JX466355	Germany: Bad Reichenhall	JX466404	no	no
Pti317JX466433	*P. clandestina*	F2	Pcl237JX466355	Turkey: Eskisehir, Nort of Asagi Caglan village	JX466433	no	no
Pti342KP659534	*P. clandestina*	F2	Pcl237JX466355	Italy: Sicilia, Messina, Nebrodi Natural Park, along the SS289 km road. 27, near Fossa di Lupo	KP659534	no	no
Pti346KP659538	*P. clandestina*	F2	Pcl237JX466355	Italy: Sicilia, Messina, Nebrodi Natural Park, along the SS289 km road. 27, near Fossa di Lupo	KP659538	no	no
Pti361KP659553	*P. clandestina*	F2	Pcl237JX466355	Iran: Azarbaijan, East, Kaleybar county, on the way towards Aynaloo-Vaygan, ca. 5 km SE Vaygan, beside the main road	KP659553	no	no
Pti377MK811862	*P. clandestina*	F2	Pcl237JX466355	Norway: Oppland, Vang, Grindaheim, Vang church	MK811862	no	no
Pti7357	*P. clandestina*	F2	Pcl237JX466355	Switzerland: Martigny, Fully, route des Follateres, about 1926 Fully	PZ290988	yes	no
Pti7383	*P. clandestina*	F2	Pcl237JX466355	Switzerland: Martigny, Fully, route des Follateres, about 1926 Fully	PZ290989	yes	no
Pcl254JX466382	*P. clandestina*	F2	Pcl254JX466382	Italy: Friuli-Venezia Giulia, Camporosso	JX466382	no	yes
Pti257JX466385	*P. clandestina*	F2	Pcl254JX466382	Italy: Friuli-Venezia Giulia, Camporosso	JX466385	no	no
Pti313JX466428	*P. clandestina*	F2	Pcl254JX466382	Turkey: Canakkale, Lapseki, north of Dumanli village	JX466428	no	no
Pti326JX466442	*P. clandestina*	F2	Pcl254JX466382	Turkey: Cankiri, Cankiri, south of Ilgaz	JX466442	no	no

Sequence code	Species (this study)	Clade/subclade	Haplotype representative	Locality	GenBank	Newly generated	Haplotype in Figure 1
Pti351KP659543	*P. clandestina*	F2	Pcl254JX466382	Slovakia: Trnava Region, Smolenice, road from Smolenicky Zámok castle, towards Smolenice, next to a small reservoir	KP659543	no	no
Pti352KP659544	*P. clandestina*	F2	Pcl254JX466382	Slovakia: Trnava Region, Smolenice, road from Smolenicky Zámok castle, towards Smolenice, next to a small reservoir	KP659544	no	no
Pti353KP659545	*P. clandestina*	F2	Pcl254JX466382	Slovakia: Trnava Region, Smolenice, road from Smolenicky Zámok castle, towards Smolenice, next to a small reservoir	KP659545	no	no
Pti369KP659561	*P. clandestina*	F2	Pcl254JX466382	Iran: Azarbaijan, East, Kaleybar county, on the way towards Aynaloo-Vaygan, ca. 5 km SE Vaygan, beside the main road	KP659561	no	no
Pti370KP659562	*P. clandestina*	F2	Pcl254JX466382	Iran: Azarbaijan, East, Kaleybar county, on the way towards Aynaloo-Vaygan, ca. 5 km SE Vaygan, beside the main road	KP659562	no	no
Pti6JF756986	*P. clandestina*	F4	Pti6JF756986	Spain: Ciudad Real, Minas de San Quintín	JF756986	no	yes
Pti97JX466202	*P. clandestina*	F4	Pti6JF756986	Morocco: Ifrane, Forêt Sidi	JX466202	no	no
Pti101JX466207	*P. clandestina*	F4	Pti101JX466207	Morocco: Medium Atlas, Fes, Taza, near Fez	JX466207	no	yes
Pti130JX466235	*P. clandestina*	F4	Pti130JX466235	Spain: Cáceres, Parque Natural de Monfragüe	JX466235	no	yes
Pti134JX466239	*P. clandestina*	F4	Pti134JX466239	Spain: Cáceres, Parque Natural de Monfragüe	JX466239	no	yes
Pti306JX466419	*P. clandestina*	F2	Pti306JX466419	Greece: Maries, Thasos island	JX466419	no	yes
Pti373MK812477	*P. clandestina*	F2	Pti306JX466419	Norway: Rogaland, Stavanger, Hillevag, north of junction Hamneveien	MK812477	no	no
Pti381EU266085	*P. clandestina*	F2	Pti306JX466419	Hungary: Vertes Mountain	EU266085	no	no
Pti344KP659536	*P. clandestina*	F4	Pti344KP659536	Italy: Sicily, Messina, Nebrodi Natural Park, along the SS289 km road. 27, near Fossa di Lupo	KP659536	no	yes
Pti7320	*P. clandestina*	F2	Pti7320	Switzerland: Martigny, Fully, route des Follateres, about 1926 Fully	PZ290990	yes	yes
Pti7326	*P. clandestina*	F2	Pti7326	Switzerland: Martigny, Fully, route des Follateres, about 1926 Fully	PZ290991	yes	yes
Pti7411	*P. clandestina*	F2	Pti7411	Switzerland: Bourg-Saint-Pierre, Forêt des Trones	PZ290992	yes	yes
Pco1DQ273855	*P. coleae*	D	Pco1DQ273855	USA: California, dirt road 12,9 km northeast of Coloma	DQ273855	no	yes
Pco4DQ273858	*P. coleae*	D	Pco1DQ273855	USA: California, Nacimiento Road	DQ273858	no	no
Pco2DQ273856	*P. coleae*	D	Pco2DQ273856	USA: California, along CA 96, 13,7 km east of intersection with Beaver Creek Road	DQ273856	no	yes
Pco7JF756968	*P. coleae*	D	Pco2DQ273856	USA: California, Sonoma Co., Sonoma lake	JF756968	no	no
Pco1026ITS	*P. coleae*	D	Pco2DQ273856	USA: California, Lake Co.	PZ290993	yes	no
Pco5JF756966	*P. coleae*	D	Pco5JF756966	USA: California, Sonoma Co., Sonoma lake	JF756966	no	yes
Pco6JF756967	*P. coleae*	D	Pco5JF756966	USA: California, Lake Co., Mendocino National Forest, Mendocino lake county between Upper lake and Lake Pillsbury	JF756967	no	no
Pco1023ITS	*P. coleae*	D	Pco1023ITS	USA: California, Sonoma Co., Sonoma lake	PZ290994	yes	yes
Pco3DQ273857	*P. coleae*	D	Pco1023ITS	USA: California, Tehama Co. along CA 32 above Potato Patch Campground	DQ273857	no	no
Pco8GU979822	*P. coleae*	D	Pco1023ITS	USA: Oregon	GU979822	no	no
Pco1024ITS	*P. coleae*	D	Pco1023ITS	USA: California, Lake Co., Mendocino National Forest, between Upper lake and Lake Pillsbury	PZ290995	yes	no
Pco1027ITS	*P. coleae*	D	Pco1023ITS	USA: California, Lake Co.	PZ290996	yes	no
Pco1028ITS	*P. coleae*	D	Pco1023ITS	USA: California, Mendocino Co.	PZ290997	yes	no
Pco1029ITS	*P. coleae*	D	Pco1023ITS	USA: California, Lake Co.	PZ290998	yes	no
Pco1031ITS	*P. coleae*	D	Pco1023ITS	USA: California, Sonoma Co., Sonoma lake	PZ290999	yes	no
Pco1032ITS	*P. coleae*	D	Pco1023ITS	USA: California, Sonoma Co., Sonoma lake	PZ291000	yes	no
Pco1033ITS	*P. coleae*	D	Pco1023ITS	USA: California, Sonoma Co., Sonoma lake	PZ291001	yes	no
Pco1030ITS	*P. coleae*	D	Pco1030ITS	USA: California Sonoma Co., Sonoma lake	PZ291002	yes	yes
Pcr1JF756970	*P. cryptotiliacea*	B	Pcr1JF756970	Spain: Madrid, El Pardo	JF756970	no	yes
Pcr2JF756975	*P. cryptotiliacea*	B	Pcr1JF756970	Spain: Ciudad Real, Mina de San Quintín	JF756975	no	no

Sequence code	Species (this study)	Clade/subclade	Haplotype representative	Locality	GenBank	Newly generated	Haplotype in Figure 1
Pcr3JF756974	*P. cryptotiliacea*	B	Pcr1JF756970	Spain: Ciudad Real, Castillo de Calatrava	JF756974	no	no
Pcr5JF756969	*P. cryptotiliacea*	B	Pcr1JF756970	Spain: Madrid, El Pardo	JF756969	no	no
Pcr6JF756973	*P. cryptotiliacea*	B	Pcr1JF756970	Spain: Cáceres, Monfragüe	JF756973	no	no
Pcr7JF756971	*P. cryptotiliacea*	B	Pcr1JF756970	Spain: Cáceres, Monfragüe	JF756971	no	no
Pcr8JX466476	*P. cryptotiliacea*	B	Pcr1JF756970	Spain: Madrid	JX466476	no	no
Pcr9JX466477	*P. cryptotiliacea*	B	Pcr1JF756970	Spain: Ciudad Real	JX466477	no	no
Pcr4JF756972	*P. cryptotiliacea*	B	Pcr4JF756972	Spain: Cáceres, Monfragüe	JF756972	no	yes
Pcr6988	*P. cryptotiliacea*	B	Pcr6988	Spain: Madrid, Ciudad Universitaria	PZ291003	yes	yes
Pme192JX466306	*P. mediterranea*	F5*	Pme192JX466306	Spain: Mallorca, Municipio de Benyalbufar, Finca de Planicie	JX466306	no	yes
Pme221JX466348	*P. mediterranea*	F5*	Pme192JX466306	Italy: Toscana, Poggioferro	JX466348	no	no
Pme225JX466352	*P. mediterranea*	F5*	Pme192JX466306	Italy: Toscana, Poggioferro	JX466352	no	no
Pti3JF756987	*P. mediterranea*	F5*	Pme192JX466306	Tunisia: Gobernación de Jendouba, near Al Mawajin	JF756987	no	no
Pti51JX466157	*P. mediterranea*	F5*	Pme192JX466306	Spain: Tenerife, La Escalona, rocks of the ravine	JX466157	no	no
Pti203JX466317	*P. mediterranea*	F5*	Pme192JX466306	Italy: Sardegna, Bosa, Badde Orca	JX466317	no	no
Pti219JX466346	*P. mediterranea*	F5*	Pme192JX466306	Italy: Toscana, Poggioferro	JX466346	no	no
Pti220JX466347	*P. mediterranea*	F5*	Pme192JX466306	Italy: Toscana, Poggioferro	JX466347	no	no
Pti222JX466349	*P. mediterranea*	F5*	Pme192JX466306	Italy: Toscana, Poggioferro	JX466349	no	no
Pti227JX466354	*P. mediterranea*	F5*	Pme192JX466306	Italy: Toscana, Poggioferro	JX466354	no	no
Pti228JX466336	*P. mediterranea*	F5*	Pme192JX466306	Italy: Toscana, Ansedonia	JX466336	no	no
Pti230JX466338	*P. mediterranea*	F5*	Pme192JX466306	Italy: Toscana, Ansedonia	JX466338	no	no
Pti231JX466339	*P. mediterranea*	F5*	Pme192JX466306	Italy: Toscana, Ansedonia	JX466339	no	no
Pti232JX466340	*P. mediterranea*	F5*	Pme192JX466306	Italy: Toscana, Ansedonia	JX466340	no	no
Pti234JX466342	*P. mediterranea*	F5*	Pme192JX466306	Italy: Toscana, Ansedonia	JX466342	no	no
Pti307JX466420	*P. mediterranea*	F5*	Pme192JX466306	Greece: Maries, Thasos island	JX466420	no	no
Pti309JX466423	*P. mediterranea*	F5*	Pme192JX466306	Greece: Samothraki island, Daphnes	JX466423	no	no
Pti312JX466426	*P. mediterranea*	F5*	Pme192JX466306	Greece: Samothraki island, Daphnes	JX466426	no	no
Pti314JX466429	*P. mediterranea*	F5*	Pme192JX466306	Turkey: Prov. Of Canakkale, Biga, south of Bakacik	JX466429	no	no
Pti328KP659520	*P. mediterranea*	F5*	Pme192JX466306	Italy: Sicilia, Santuario de Gibilmanna, Frazione di Gibilmanna, Cefalú	KP659520	no	no
Pti329KP659521	*P. mediterranea*	F5*	Pme192JX466306	Italy: Sicilia, Santuario de Gibilmanna, Frazione di Gibilmanna, Cefalú	KP659521	no	no
Pti330KP659522	*P. mediterranea*	F5*	Pme192JX466306	Italy: Sicilia, Santuario de Gibilmanna, Frazione di Gibilmanna, Cefalú	KP659522	no	no
Pti331KP659523	*P. mediterranea*	F5*	Pme192JX466306	Italy: Sicilia, Santuario de Gibilmanna, Frazione di Gibilmanna, Cefalú	KP659523	no	no
Pti333KP659525	*P. mediterranea*	F5*	Pme192JX466306	Italy: Sicilia, Santuario de Gibilmanna, Frazione di Gibilmanna, Cefalú	KP659525	no	no
Pti334KP659526	*P. mediterranea*	F5*	Pme192JX466306	Italy: Sicilia, Santuario de Gibilmanna, Frazione di Gibilmanna, Cefalú	KP659526	no	no
Pti335KP659527	*P. mediterranea*	F5*	Pme192JX466306	Italy: Sicilia, Santuario de Gibilmanna, Frazione di Gibilmanna, Cefalú	KP659527	no	no
Pti336KP659528	*P. mediterranea*	F5*	Pme192JX466306	Italy: Sicilia, Santuario de Gibilmanna, Frazione di Gibilmanna, Cefalú	KP659528	no	no
Pti339KP659531	*P. mediterranea*	F5*	Pme192JX466306	Italy: Sicilia, Santuario de Gibilmanna, Frazione di Gibilmanna, Cefalú	KP659531	no	no
Pti340KP659532	*P. mediterranea*	F5*	Pme192JX466306	Italy: Sicilia, Santuario de Gibilmanna, Frazione di Gibilmanna, Cefalú	KP659532	no	no
Pti341KP659533	*P. mediterranea*	F5*	Pme192JX466306	Italy: Sicily, Messina, Nebrodi Natural Park, along the SS289 km road. 27, near Fossa di Lupo	KP659533	no	no
Pti343KP659535	*P. mediterranea*	F5*	Pme192JX466306	Italy: Sicily, Messina, Nebrodi Natural Park, along the SS289 km road. 27, near Fossa di Lupo	KP659535	no	no
Pti345KP659537	*P. mediterranea*	F5*	Pme192JX466306	Italy: Sicily, Messina, Nebrodi Natural Park, along the SS289 km road. 27, near Fossa di Lupo	KP659537	no	no
Pti347KP659539	*P. mediterranea*	F5*	Pme192JX466306	Italy: Sicily, Messina, Nebrodi Natural Park, along the SS289 km road. 27, near Fossa di Lupo	KP659539	no	no
Pti7416	*P. mediterranea*	F5*	Pme192JX466306	Switzerland: Martigny: Fully, route des Follateres, about 1926 Fully	PZ291004	yes	no

Sequence code	Species (this study)	Clade/subclade	Haplotype representative	Locality	GenBank	Newly generated	Haplotype in Figure 1
Pme194JX466308	*P. mediterranea*	F5	Pme194JX466308	Spain: Mallorca, Municipio de Benyalbufar, Finca de Planicie	JX466308	no	yes
Pti1JF756977	*P. mediterranea*	F5	Pme194JX466308	Turkey: Bursa, Uludaj road side	JF756977	no	no
Pti223JX466350	*P. mediterranea*	F5	Pme194JX466308	Italy: Toscana, Poggioferro	JX466350	no	no
Pti308JX466421	*P. mediterranea*	F5	Pme194JX466308	Greece: Maries, Thasos island	JX466421	no	no
Pme197JX466311	*P. mediterranea*	F5	Pme197JX466311	Spain: Mallorca, Son Ufanes, cultivated fields of the Church of Sant Michel	JX466311	no	yes
Pme39JX466145	*P. mediterranea*	F5	Pme197JX466311	Spain: Tenerife, Degollada de Teno Alto	JX466145	no	no
Pme41JX466147	*P. mediterranea*	F5	Pme197JX466311	Spain: Tenerife, Degollada de Teno Alto	JX466147	no	no
Pme198JX466312	*P. mediterranea*	F5	Pme197JX466311	Tunisia: Jendouba Governorate, near Al Mawajin Natural Park	JX466312	no	no
Pti2JF756982	*P. mediterranea*	F5	Pme197JX466311	Fance: Provence, road between Saint-Tropez and Toulon	JF756982	no	no
Pti10JF756985	*P. mediterranea*	F5	Pme197JX466311	Italy: Val Trompia, Lodrino	JF756985	no	no
Pti16JF756990	*P. mediterranea*	F5	Pme197JX466311	Spain: Mallorca, Binifaldo entrance	JF756990	no	no
Pti22JX466320	*P. mediterranea*	F5	Pme197JX466311	Italy: Sardegna, Bosa, Badde Orca	JX466320	no	no
Pti33JX466139	*P. mediterranea*	F5	Pme197JX466311	Spain: Tenerife, Degollada de Teno Alto	JX466139	no	no
Pti34JX466140	*P. mediterranea*	F5	Pme197JX466311	Spain: Tenerife, Degollada de Teno Alto	JX466140	no	no
Pti35JX466141	*P. mediterranea*	F5	Pme197JX466311	Spain: Tenerife, Degollada de Teno Alto	JX466141	no	no
Pti36JX466142	*P. mediterranea*	F5	Pme197JX466311	Spain: Tenerife, Degollada de Teno Alto	JX466142	no	no
Pti37JX466143	*P. mediterranea*	F5	Pme197JX466311	Spain: Tenerife, Degollada de Teno Alto	JX466143	no	no
Pti38JX466144	*P. mediterranea*	F5	Pme197JX466311	Spain: Tenerife, Degollada de Teno Alto	JX466144	no	no
Pti40JX466146	*P. mediterranea*	F5	Pme197JX466311	Spain: Tenerife, Degollada de Teno Alto	JX466146	no	no
Pti42JX466148	*P. mediterranea*	F5	Pme197JX466311	Spain: Tenerife, Degollada de Teno Alto	JX466148	no	no
Pti46JX466152	*P. mediterranea*	F5	Pme197JX466311	Spain: Tenerife, La Escalona, rocky outcrops of the ravine	JX466152	no	no
Pti47JX466153	*P. mediterranea*	F5	Pme197JX466311	Spain: Tenerife, La Escalona, rocky outcrops of the ravine	JX466153	no	no
Pti48JX466154	*P. mediterranea*	F5	Pme197JX466311	Spain: Tenerife, La Escalona, rocky outcrops of the ravine	JX466154	no	no
Pti49JX466155	*P. mediterranea*	F5	Pme197JX466311	Spain: Tenerife, La Escalona, rocky outcrops of the ravine	JX466155	no	no
Pti50JX466156	*P. mediterranea*	F5	Pme197JX466311	Spain: Tenerife, La Escalona, roquedos del barranco	JX466156	no	no
Pti91JX466206	*P. mediterranea*	F5	Pme197JX466311	Morocco: Medium Atlas, Fes, Mulai-Idriss, climbing Mount J-Z	JX466206	no	no
Pti102JX466208	*P. mediterranea*	F5	Pme197JX466311	Morocco: Tánger-Tetouán, Chauen	JX466208	no	no
Pti104JX466210	*P. mediterranea*	F5	Pme197JX466311	Spain: Andalucía, Cádiz, La Sauceda	JX466210	no	no
Pti105JX466211	*P. mediterranea*	F5	Pme197JX466311	Spain: Andalucía, Cádiz, La Sauceda	JX466211	no	no
Pti109JX466215	*P. mediterranea*	F5	Pme197JX466311	Spain: Andalucía, Cádiz, La Sauceda	JX466215	no	no
Pti110JX466216	*P. mediterranea*	F5	Pme197JX466311	Spain: Andalucía, Cádiz, La Sauceda	JX466216	no	no
Pti111JX466217	*P. mediterranea*	F5	Pme197JX466311	Spain: Andalucía, Cádiz, La Sauceda	JX466217	no	no
Pti183JX466297	*P. mediterranea*	F5	Pme197JX466311	Spain: Valencia, Castellón, Chóvar	JX466297	no	no
Pti184JX466298	*P. mediterranea*	F5	Pme197JX466311	Spain: Valencia, Castellón, Chóvar	JX466298	no	no
Pti185JX466299	*P. mediterranea*	F5	Pme197JX466311	Spain: Valencia, Castellón, Chóvar	JX466299	no	no
Pti186JX466300	*P. mediterranea*	F5	Pme197JX466311	Spain: Valencia, Castellón, Chóvar	JX466300	no	no
Pti187JX466301	*P. mediterranea*	F5	Pme197JX466311	Spain: Valencia, Castellón, Chóvar	JX466301	no	no
Pti188JX466302	*P. mediterranea*	F5	Pme197JX466311	Spain: Valencia, Castellón, Chóvar	JX466302	no	no
Pti189JX466303	*P. mediterranea*	F5	Pme197JX466311	Spain: Valencia, Castellón, Chóvar	JX466303	no	no
Pti190JX466304	*P. mediterranea*	F5	Pme197JX466311	Spain: Valencia, Castellón, Chóvar	JX466304	no	no
Pti191JX466305	*P. mediterranea*	F5	Pme197JX466311	Spain: Valencia, Castellón, Chóvar	JX466305	no	no
Pti193JX466307	*P. mediterranea*	F5	Pme197JX466311	Spain: Mallorca, Municipio de Benyalbufar, Finca de Planicie	JX466307	no	no

Sequence code	Species (this study)	Clade/subclade	Haplotype representative	Locality	GenBank	Newly generated	Haplotype in Figure 1
Pti195JX466309	*P. mediterranea*	F5	Pme197JX466311	Spain: Mallorca, entrance to Benifaldo	JX466309	no	no
Pti196JX466310	*P. mediterranea*	F5	Pme197JX466311	Spain: Mallorca, Son Ufanes, Campanet, Church of Sant Michel	JX466310	no	no
Pti199JX466313	*P. mediterranea*	F5	Pme197JX466311	Italy: Sardegna, Bosa, Badde Orca	JX466313	no	no
Pti200JX466314	*P. mediterranea*	F5	Pme197JX466311	Italy: Sardegna, Bosa, Badde Orca	JX466314	no	no
Pti202JX466316	*P. mediterranea*	F5	Pme197JX466311	Italy: Sardegna, Bosa, Badde Orca	JX466316	no	no
Pti204JX466318	*P. mediterranea*	F5	Pme197JX466311	Italy: Sardegna, Bosa, Badde Orca	JX466318	no	no
Pti205JX466319	*P. mediterranea*	F5	Pme197JX466311	Italy: Sardegna, Bosa, Badde Orca	JX466319	no	no
Pti206JX466321	*P. mediterranea*	F5	Pme197JX466311	Italy: Sardegna, Caldoso, San Antonio de Gallura	JX466321	no	no
Pti208JX466322	*P. mediterranea*	F5	Pme197JX466311	France: Provence, Côte de Provence, road from Saint Tropez to Toulon on N7, Domaine de Champeaux	JX466322	no	no
Pti211JX466362	*P. mediterranea*	F5	Pme197JX466311	Italy: Lodrino, Val Trompia	JX466362	no	no
Pti212JX466363	*P. mediterranea*	F5	Pme197JX466311	Italy: Lodrino, Val Trompia	JX466363	no	no
Pti213JX466364	*P. mediterranea*	F5	Pme197JX466311	Italy: Lodrino, Val Trompia	JX466364	no	no
Pti214JX466365	*P. mediterranea*	F5	Pme197JX466311	Italy: Lodrino, Val Trompia	JX466365	no	no
Pti215JX466366	*P. mediterranea*	F5	Pme197JX466311	Italy: Lodrino, Val Trompia	JX466366	no	no
Pti216JX466367	*P. mediterranea*	F5	Pme197JX466311	Italy: Lodrino, Val Trompia	JX466367	no	no
Pti217JX466368	*P. mediterranea*	F5	Pme197JX466311	Italy: Lodrino, Val Trompia	JX466368	no	no
Pti226JX466353	*P. mediterranea*	F5	Pme197JX466311	Italy: Toscana, Poggioferro	JX466353	no	no
Pti229JX466337	*P. mediterranea*	F5	Pme197JX466311	Italy: Toscana, Ansedonia	JX466337	no	no
Pti236JX466344	*P. mediterranea*	F5	Pme197JX466311	Italy: Toscana, Ansedonia	JX466344	no	no
Pti327KP659519	*P. mediterranea*	F5	Pme197JX466311	Italy: Sicilia, Santuario de Gibilmanna, Frazione di Gibilmanna, Cefalú	KP659519	no	no
Pti332KP659524	*P. mediterranea*	F5	Pme197JX466311	Italy: Sicilia, Santuario de Gibilmanna, Frazione di Gibilmanna, Cefalú	KP659524	no	no
Pti337KP659529	*P. mediterranea*	F5	Pme197JX466311	Italy: Sicilia, Santuario de Gibilmanna, Frazione di Gibilmanna, Cefalú	KP659529	no	no
Pti338KP659530	*P. mediterranea*	F5	Pme197JX466311	Italy: Sicilia, Santuario de Gibilmanna, Frazione di Gibilmanna, Cefalú	KP659530	no	no
Pti65JX466190	*P. mediterranea*	F5	Pti65JX466190	Spain: Gran Canaria, Galdar, between Saucillo and San José	JX466190	no	yes
Pti106JX466212	*P. mediterranea*	F5	Pti106JX466212	Spain: Cádiz, La Sauceda	JX466212	no	yes
Pti7190	*P. mediterranea*	F5	Pti7190	Spain: Cádiz, Los Alcornocales Natural Park	PZ291005	yes	yes
Pti7193	*P. mediterranea*	F5	Pti7190	Spain: Cádiz, Los Alcornocales Natural Park	PZ291006	yes	no
Pti7194	*P. mediterranea*	F5	Pti7190	Spain: Cádiz, Los Alcornocales Natural Park	PZ291007	yes	no
Pti7201	*P. mediterranea*	F5	Pti7190	Spain: Cádiz, Los Alcornocales Natural Park	PZ291008	yes	no
Ppa8JX466293	*P. pastillifera*	F1	Ppa8JX466293	Spain: Navarra, Puerto de Velate	JX466293	no	yes
Ppa10JX466290	*P. pastillifera*	F1*	Ppa10JX466290	Spain: Navarra, Puerto de Velate	JX466290	no	yes
Ppa1AY611104	*P. pastillifera*	F1*	Ppa10JX466290	Spain: Cádiz, Grazalema	AY611104	no	no
Ppa2DQ273860	*P. pastillifera*	F1*	Ppa10JX466290	Spain: Burgos, Puerto de Carrales	DQ273860	no	no
Ppa3JF756998	*P. pastillifera*	F1*	Ppa10JX466290	Spain: Cantabria, La Lomba	JF756998	no	no
Ppa4JF756999	*P. pastillifera*	F1*	Ppa10JX466290	Spain: Asturias, Saliencia	JF756999	no	no
Ppa6JX466412	*P. pastillifera*	F1*	Ppa10JX466290	Germany: Upper Bavaria, Bad Reichenhall	JX466412	no	no
Ppa9JX466289	*P. pastillifera*	F1*	Ppa10JX466290	Spain: Navarra, Puerto de Velate	JX466289	no	no
Ppa14JX466295	*P. pastillifera*	F1*	Ppa10JX466290	Spain: Navarra, Puerto de Velate	JX466295	no	no
Ppa15JX466356	*P. pastillifera*	F1*	Ppa10JX466290	Italy: Toscana, Monte Amiatta	JX466356	no	no
Ppa16JX466357	*P. pastillifera*	F1*	Ppa10JX466290	Italy: Toscana, Monte Amiatta	JX466357	no	no
Ppa18JX466359	*P. pastillifera*	F1*	Ppa10JX466290	Italy: Toscana, Monte Amiatta	JX466359	no	no

Sequence code	Species (this study)	Clade/subclade	Haplotype representative	Locality	GenBank	Newly generated	Haplotype in Figure 1
Ppa19JX466369	*P. pastillifera*	F1*	Ppa10JX466290	Italy: Friuli-Venezia Giulia, Fielis	JX466369	no	no
Ppa20JX466370	*P. pastillifera*	F1*	Ppa10JX466290	Italy: Friuli-Venezia Giulia, Fielis	JX466370	no	no
Ppa21JX466371	*P. pastillifera*	F1*	Ppa10JX466290	Italy: Friuli-Venezia Giulia, Fielis	JX466371	no	no
Ppa22JX466372	*P. pastillifera*	F1*	Ppa10JX466290	Italy: Friuli-Venezia Giulia, Fielis	JX466372	no	no
Ppa23JX466373	*P. pastillifera*	F1*	Ppa10JX466290	Italy: Friuli-Venezia Giulia, Fielis	JX466373	no	no
Ppa24JX466374	*P. pastillifera*	F1*	Ppa10JX466290	Italy: Friuli-Venezia Giulia, Fielis	JX466374	no	no
Ppa25JX466375	*P. pastillifera*	F1*	Ppa10JX466290	Italy: Friuli-Venezia Giulia, Fielis	JX466375	no	no
Ppa26JX466376	*P. pastillifera*	F1*	Ppa10JX466290	Italy: Friuli-Venezia Giulia, Fielis	JX466376	no	no
Ppa27JX466377	*P. pastillifera*	F1*	Ppa10JX466290	Italy: Friuli-Venezia Giulia, Fielis	JX466377	no	no
Ppa28JX466378	*P. pastillifera*	F1*	Ppa10JX466290	Italy: Friuli-Venezia Giulia, Fielis	JX466378	no	no
Ppa29JX466387	*P. pastillifera*	F1*	Ppa10JX466290	Italy: Friuli-Venezia Giulia, Camporosso	JX466387	no	no
Ppa30JX466388	*P. pastillifera*	F1*	Ppa10JX466290	Italy: Friuli-Venezia Giulia, Camporosso	JX466388	no	no
Ppa50KX457685	*P. pastillifera*	F1*	Ppa10JX466290	Armenia: Prov. of Tavush, Dilijan, Dilijan National Park, around Haghartsin Monastery	KX457685	no	no
Ppa7322	*P. pastillifera*	F1*	Ppa10JX466290	Switzerland: Collonges, Plex, Plex parking	PZ291009	yes	no
Ppa7323	*P. pastillifera*	F1*	Ppa10JX466290	Spain: Guipúzcoa, Aratz-Aizkorri Natural Park, Aizgorri beech forest	PZ291014	yes	no
Ppa7328	*P. pastillifera*	F1*	Ppa10JX466290	Spain: Guipúzcoa, Aratz-Aizkorri Natural Park, Aizgorri beech forest	PZ291010	yes	no
Ppa7329	*P. pastillifera*	F1*	Ppa10JX466290	Switzerland: Collonges, Plex, Plex parking	PZ291017	yes	no
Ppa7334	*P. pastillifera*	F1*	Ppa10JX466290	Spain: Guipúzcoa, Aratz-Aizkorri Natural Park, Aizgorri beech forest	PZ291016	yes	no
Ppa7335	*P. pastillifera*	F1*	Ppa10JX466290	Spain: Guipúzcoa, Aratz-Aizkorri Natural Park, Aizgorri beech forest	PZ291018	yes	no
Ppa7339	*P. pastillifera*	F1*	Ppa10JX466290	Switzerland: Collonges, Plex, Plex parking	PZ291015	yes	no
Ppa7404	*P. pastillifera*	F1*	Ppa10JX466290	Switzerland: Collonges, Plex, Plex parking	PZ291011	yes	no
Ppa11JX466291	*P. pastillifera*	F1	Ppa11JX466291	Spain: Navarra, Puerto de Velate	JX466291	no	yes
Ppa7JX466457	*P. pastillifera*	F1	Ppa11JX466291	Norway: Hordaland, Bergen, Sietten, ca 100m northeast of Slettebakken church	JX466457	no	no
Ppa12JX466292	*P. pastillifera*	F1	Ppa11JX466291	Spain: Navarra, Puerto de Velate	JX466292	no	no
Ppa13JX466294	*P. pastillifera*	F1	Ppa11JX466291	Spain: Navarra, Puerto de Velate	JX466294	no	no
Ppa17JX466358	*P. pastillifera*	F1	Ppa11JX466291	Italy: Toscana, Monte Amiatta	JX466358	no	no
Ppa32JX466447	*P. pastillifera*	F1	Ppa11JX466291	United Kingdom: Wales, road A5, between Rhydlydan and Glasfryn	JX466447	no	no
Ppa33JX466448	*P. pastillifera*	F1	Ppa11JX466291	United Kingdom: Wales, road A5, between Rhydlydan and Glasfryn	JX466448	no	no
Ppa34JX466449	*P. pastillifera*	F1	Ppa11JX466291	United Kingdom: Wales, road A5, between Rhydlydan and Glasfryn	JX466449	no	no
Ppa35JX466455	*P. pastillifera*	F1	Ppa11JX466291	Norway: 12, Bergen, Sietten, ca 100m northeast of Slettebakken church	JX466455	no	no
Ppa36JX466456	*P. pastillifera*	F1	Ppa11JX466291	Norway: 12, Bergen, Sietten, ca 100m northeast of Slettebakken church	JX466456	no	no
Ppa37JX466458	*P. pastillifera*	F1	Ppa11JX466291	Norway: 12, Bergen, Sietten, ca 100m northeast of Slettebakken church	JX466458	no	no
Ppa38JX466459	*P. pastillifera*	F1	Ppa11JX466291	Norway: 12, Bergen, Sietten, ca 100m northeast of Slettebakken church	JX466459	no	no
Ppa39JX466460	*P. pastillifera*	F1	Ppa11JX466291	Norway: 12, Bergen, Sietten, ca 100m northeast of Slettebakken church	JX466460	no	no
Ppa40JX466461	*P. pastillifera*	F1	Ppa11JX466291	Norway: 12, Bergen, Sietten, ca 100m northeast of Slettebakken church	JX466461	no	no
Ppa41JX466462	*P. pastillifera*	F1	Ppa11JX466291	Norway: 12, Bergen, Sietten, ca 100m northeast of Slettebakken church	JX466462	no	no
Ppa42JX466422	*P. pastillifera*	F1	Ppa11JX466291	Greece: Maries, Thasos island	JX466422	no	no
Ppa45MK812633	*P. pastillifera*	F1	Ppa11JX466291	Norway: Rogaland, Sandnes, Hoyland kirke	MK812633	no	no
Ppa47MK812554	*P. pastillifera*	F1	Ppa11JX466291	Norway: Rogaland, Stavanger, at crossing of roads Auglendsveien and Stranddalsveien	MK812554	no	no
Ppa48MK812400	*P. pastillifera*	F1	Ppa11JX466291	Norway: Hordaland, Etne, Taraldsoy	MK812400	no	no
Ppa49MK812365	*P. pastillifera*	F1	Ppa11JX466291	Norway: Rogaland, Hjelmeland, Ardal church	MK812365	no	no
Ppa7321	*P. pastillifera*	F1	Ppa11JX466291	Spain: Segovia, Hayedo de la Pedrosa	PZ291012	yes	no

Sequence code	Species (this study)	Clade/subclade	Haplotype representative	Locality	GenBank	Newly generated	Haplotype in Figure 1
Ppa7410	*P. pastillifera*	F1	Ppa11JX466291	Spain: Segovia, Hayedo de la Pedrosa	PZ291013	yes	no
Ppa31JX466413	*P. pastillifera*	F1	Ppa31JX466413	Germany: Bad Reichenhall	JX466413	no	yes
Ppa43JX466427	*P. pastillifera*	F1	Ppa43JX466427	Greece: Samothraki island, Daphnes	JX466427	no	yes
Ppa44JX466430	*P. pastillifera*	F1*	Ppa44JX466430	Turkey: Prov. of Canakale, distr. Lapseki, north of village Dumanli	JX466430	no	yes
Ppa46MK812564	*P. pastillifera*	F1	Ppa46MK812564	Norway: Rogaland, Hjelmeland, Hjelmelandsvagen, at the ferry quay	MK812564	no	yes
Pqu1DQ273842	*P. quercina*	C	Pqu1DQ273842	Spain: Madrid, carretera de Aldea del Fresno a San Martín de Valdeiglesias	DQ273842	no	yes
Pqu4DQ273845	*P. quercina*	C	Pqu4DQ273845	Spain: Madrid, Los Molinos to Navacerrada road	DQ273845	no	yes
Pqu6DQ273847	*P. quercina*	C	Pqu4DQ273845	Spain: La Rioja, Montenegro de Cameros	DQ273847	no	no
Pqu7DQ273848	*P. quercina*	C	Pqu4DQ273845	Spain: Guadalajara, Selas	DQ273848	no	no
Pqu9HM017023	*P. quercina*	C	Pqu9HM017023	Turkey: Prov. of Eskisehir, Tandir village	HM017023	no	yes
Pqu9JF757001	*P. quercina*	C	Pqu9HM017023	Turkey: Prov. of Eskisehir, Tandir village	JF757001	no	no
Pqu10HM017024	*P. quercina*	C	Pqu10HM017024	Turkey: Prov. of Bursa, Uludaj	HM017024	no	yes
Pqu2DQ273843	*P. quercina*	C	Pqu10HM017024	Spain: Madrid, Aldea del Fresno, poplar	DQ273843	no	no
Pqu3DQ273844	*P. quercina*	C	Pqu10HM017024	Spain: Madrid, Matalpino	DQ273844	no	no
Pqu5DQ273846	*P. quercina*	C	Pqu10HM017024	Spain: La Rioja, Montenegro de Cameros	DQ273846	no	no
Pqu8HM017022	*P. quercina*	C	Pqu10HM017024	Turkey: Prov. of Canakkale, Biga, south of Bakacik	HM017022	no	no
Pqu8JF757000	*P. quercina*	C	Pqu10HM017024	Turkey: Prov. of Canakkale, Biga, south of Bakacik	JF757000	no	no
Pqu10JF757002	*P. quercina*	C	Pqu10HM017024	Turkey: Prov. of Bursa, Uludaj	JF757002	no	no
Pqu12HM017021	*P. quercina*	C	Pqu12HM017021	Morocco: Chaguen, near yo Chaoeun	HM017021	no	yes
Pque6987	*P. quercina*	C	Pque6987	Spain: Madrid, Ciudad Universitaria	PZ291019	yes	yes
Pque7038	*P. quercina*	C	Pque7038	Spain: Madrid, Ciudad Universitaria	PZ291020	yes	yes
Pque7039	*P. quercina*	C	Pque7039	Spain: Madrid, Ciudad Universitaria	PZ291021	yes	yes
PtiCH060016	*Parmelina sp.*	A2	PtiCH060016	China	KY128195	no	yes
Pat7386	*P. tiliacea*	F3	Pat7386	Switzerland: Martigny, Fully, route des Follateres, about 1926 Fully	PZ291022	yes	yes
Pti11JF756989	*P. tiliacea*	F3	Pti11JF756989	Germany: Bavaria, Oberfranken, Kteis Forchheim, Ehrenburg	JF756989	no	yes
Pti12JF756991	*P. tiliacea*	F3	Pti11JF756989	Austria: Salzburg, Mittersill town	JF756991	no	no
Pti81JX466168	*P. tiliacea*	F3	Pti11JF756989	Spain: Gran Canaria, above Valleseco, in cross of San Mateo and Antenara GC-21 / GC-231	JX466168	no	no
Pti116JX466222	*P. tiliacea*	F3	Pti11JF756989	Spain: Cádiz, Grazalema	JX466222	no	no
Pti133JX466238	*P. tiliacea*	F3	Pti11JF756989	Spain: Cáceres, Parque Natural de Monfragüe	JX466238	no	no
Pti135JX466240	*P. tiliacea*	F3	Pti11JF756989	Spain: Cáceres, Parque Natural de Monfragüe	JX466240	no	no
Pti151JX466257	*P. tiliacea*	F3	Pti11JF756989	Portugal: Tras-os-Montes, Bragança, Sabor River Valley, near Lagoa, and at the entrance to the village	JX466257	no	no
Pti153JX466261	*P. tiliacea*	F3	Pti11JF756989	Spain: Madrid, El Escorial, surroundings of Silla de Felipe II	JX466261	no	no
Pti154JX466262	*P. tiliacea*	F3	Pti11JF756989	Spain: Madrid, El Escorial, surroundings of Silla de Felipe II	JX466262	no	no
Pti155JX466263	*P. tiliacea*	F3	Pti11JF756989	Spain: Madrid, El Escorial, surroundings of Silla de Felipe II	JX466263	no	no
Pti171JX466270	*P. tiliacea*	F3	Pti11JF756989	Spain: Segovia, Enebral de Moral de Hornuez	JX466270	no	no
Pti172JX466271	*P. tiliacea*	F3	Pti11JF756989	Spain: Segovia, Enebral de Moral de Hornuez	JX466271	no	no
Pti173JX466272	*P. tiliacea*	F3	Pti11JF756989	Spain: Segovia, Enebral de Moral de Hornuez	JX466272	no	no
Pti174JX466273	*P. tiliacea*	F3	Pti11JF756989	Spain: Segovia, Enebral de Moral de Hornuez	JX466273	no	no
Pti175JX466274	*P. tiliacea*	F3	Pti11JF756989	Spain: Segovia, Enebral de Moral de Hornuez	JX466274	no	no
Pti176JX466275	*P. tiliacea*	F3	Pti11JF756989	Spain: Segovia, Enebral de Moral de Hornuez	JX466275	no	no
Pti177JX466276	*P. tiliacea*	F3	Pti11JF756989	Spain: Segovia, Enebral de Moral de Hornuez	JX466276	no	no

Sequence code	Species (this study)	Clade/subclade	Haplotype representative	Locality	GenBank	Newly generated	Haplotype in Figure 1
Pti178JX466277	*P. tiliacea*	F3	Pti11JF756989	Spain: Segovia, Enebral de Moral de Hornuez	JX466277	no	no
Pti179JX466278	*P. tiliacea*	F3	Pti11JF756989	Spain: Segovia, Enebral de Moral de Hornuez	JX466278	no	no
Pti181JX466288	*P. tiliacea*	F3	Pti11JF756989	Spain: Navarra, Belagua	JX466288	no	no
Pti182JX466296	*P. tiliacea*	F3	Pti11JF756989	Spain: Castellón, Chóvar	JX466296	no	no
Pti218JX466345	*P. tiliacea*	F3	Pti11JF756989	Italy: Toscana, Poggioferro	JX466345	no	no
Pti224JX466351	*P. tiliacea*	F3	Pti11JF756989	Italy: Toscana, Poggioferro	JX466351	no	no
Pti233JX466341	*P. tiliacea*	F3	Pti11JF756989	Italy: Toscana, Ansedonia	JX466341	no	no
Pti239JX466325	*P. tiliacea*	F3	Pti11JF756989	France: Cordes, Château de Cordes	JX466325	no	no
Pti240JX466326	*P. tiliacea*	F3	Pti11JF756989	France: Cordes, Château de Cordes	JX466326	no	no
Pti242JX466327	*P. tiliacea*	F3	Pti11JF756989	Switzerland: Neuchatel, Cantón NE Neuchatel, Pierre-à-Bot	JX466327	no	no
Pti244JX466329	*P. tiliacea*	F3	Pti11JF756989	Switzerland: Neuchatel, Cantón NE Neuchatel, Pierre-à-Bot	JX466329	no	no
Pti245JX466330	*P. tiliacea*	F3	Pti11JF756989	Switzerland: Neuchatel, Cantón NE Neuchatel, Pierre-à-Bot	JX466330	no	no
Pti246JX466331	*P. tiliacea*	F3	Pti11JF756989	Switzerland: Neuchatel, Cantón NE Neuchatel, Pierre-à-Bot	JX466331	no	no
Pti247JX466332	*P. tiliacea*	F3	Pti11JF756989	Switzerland: Neuchatel, Cantón NE Neuchatel, Pierre-à-Bot	JX466332	no	no
Pti248JX466333	*P. tiliacea*	F3	Pti11JF756989	Switzerland: Neuchatel, Cantón NE Neuchatel, Pierre-à-Bot	JX466333	no	no
Pti249JX466334	*P. tiliacea*	F3	Pti11JF756989	Switzerland: Neuchatel, Cantón NE Neuchatel, Pierre-à-Bot	JX466334	no	no
Pti253JX466381	*P. tiliacea*	F3	Pti11JF756989	Italy: Friuli-Venezia Giulia, Camporosso	JX466381	no	no
Pti268JX466398	*P. tiliacea*	F3	Pti11JF756989	Austria: Salzburgo, Mittersill	JX466398	no	no
Pti270JX466400	*P. tiliacea*	F3	Pti11JF756989	Austria: Salzburgo, Mittersill	JX466400	no	no
Pti272JX466402	*P. tiliacea*	F3	Pti11JF756989	Austria: Salzburgo, Mittersill	JX466402	no	no
Pti282JX466414	*P. tiliacea*	F3	Pti11JF756989	Germany: Bavaria, Oberpfalz, Kreis Neustadt, a. d. waldnaab, Gmeinsricth	JX466414	no	no
Pti283JX466415	*P. tiliacea*	F3	Pti11JF756989	Germany: Bavaria, Oberbayern, Kreis Miesbach, N Oberisarngan	JX466415	no	no
Pti290JX466450	*P. tiliacea*	F3	Pti11JF756989	United Kingdom: Cumbria, road B6259, near Standford	JX466450	no	no
Pti291JX466451	*P. tiliacea*	F3	Pti11JF756989	United Kingdom: Cumbria, road B6259, near Standford	JX466451	no	no
Pti292JX466452	*P. tiliacea*	F3	Pti11JF756989	United Kingdom: Cumbria, road B6259, near Standford	JX466452	no	no
Pti293JX466453	*P. tiliacea*	F3	Pti11JF756989	United Kingdom: Cumbria, road B6259, near Standford	JX466453	no	no
Pti294JX466454	*P. tiliacea*	F3	Pti11JF756989	United Kingdom: Cumbria, road B6259, Warcop	JX466454	no	no
Pti297JX466465	*P. tiliacea*	F3	Pti11JF756989	Sweden: Uppland, Uppsala, Uppsala University Botanical Garden	JX466465	no	no
Pti298JX466466	*P. tiliacea*	F3	Pti11JF756989	Sweden: Uppland, Uppsala, Uppsala University Botanical Garden	JX466466	no	no
Pti299JX466467	*P. tiliacea*	F3	Pti11JF756989	Sweden: Uppland, Uppsala, Uppsala University Botanical Garden	JX466467	no	no
Pti300JX466468	*P. tiliacea*	F3	Pti11JF756989	Sweden: Uppland, Uppsala, Uppsala University Botanical Garden	JX466468	no	no
Pti301JX466469	*P. tiliacea*	F3	Pti11JF756989	Sweden: Uppland, Uppsala, Uppsala University Botanical Garden	JX466469	no	no
Pti302JX466470	*P. tiliacea*	F3	Pti11JF756989	Sweden: Uppland, Uppsala, Uppsala University Botanical Garden	JX466470	no	no
Pti303JX466471	*P. tiliacea*	F3	Pti11JF756989	Sweden: Uppland, Uppsala, Uppsala University Botanical Garden	JX466471	no	no
Pti304JX466472	*P. tiliacea*	F3	Pti11JF756989	Sweden: Uppland, Uppsala, Uppsala University Botanical Garden	JX466472	no	no
Pti310JX466424	*P. tiliacea*	F3	Pti11JF756989	Greece: Daphnes, Samothraki island	JX466424	no	no
Pti316JX466432	*P. tiliacea*	F3	Pti11JF756989	Turkey: Eskisehir, north of Asagi Caglan village	JX466432	no	no
Pti320JX466436	*P. tiliacea*	F3	Pti11JF756989	Turkey: Eskisehir, Sivirihisar Mountains	JX466436	no	no
Pti324JX466440	*P. tiliacea*	F3	Pti11JF756989	Turkey: Eskisehir, Sivirihisar Mountains	JX466440	no	no
Pti348KP659540	*P. tiliacea*	F3	Pti11JF756989	Slovakia: Trnava Region, Smolenice, road to the castle of Smolenicky Zámok, towards Smolenice, next to a small reservoir	KP659540	no	no

Sequence code	Species (this study)	Clade/subclade	Haplotype representative	Locality	GenBank	Newly generated	Haplotype in Figure 1
Pti350KP659542	*P. tiliacea*	F3	Pti11JF756989	Slovakia: Trnava Region, Smolenice, road to the castle of Smolenicky Zámok, towards Smolenice, next to a small reservoir	KP659542	no	no
Pti354KP659546	*P. tiliacea*	F3	Pti11JF756989	Slovakia: Trnava Region, Smolenice, road to the castle of Smolenicky Zámok, towards Smolenice, next to a small reservoir	KP659546	no	no
Pti355KP659547	*P. tiliacea*	F3	Pti11JF756989	Slovakia: Trnava Region, Smolenice, road to the castle of Smolenicky Zámok, towards Smolenice, next to a small reservoir	KP659547	no	no
Pti356KP659548	*P. tiliacea*	F3	Pti11JF756989	Slovakia: Trnava Region, Smolenice, road to the castle of Smolenicky Zámok, towards Smolenice, next to a small reservoir	KP659548	no	no
Pti357KP659549	*P. tiliacea*	F3	Pti11JF756989	Sweden: Skåne län, Skane, Sjôbo kommun, O. Kärrstorp church, by gravel road ca 100 m east of the church	KP659549	no	no
Pti358KP659550	*P. tiliacea*	F3	Pti11JF756989	Sweden: Skåne län, Skane, Sjôbo kommun, O. Kärrstorp church, by gravel road ca 100 m east of the church	KP659550	no	no
Pti359KP659551	*P. tiliacea*	F3	Pti11JF756989	Sweden: Skåne län, Skane, Sjôbo kommun, O. Kärrstorp church, on the church yard north side of the church	KP659551	no	no
Pti360KP659552	*P. tiliacea*	F3	Pti11JF756989	Sweden: Skåne län, Skane, Sjôbo kommun, O. Kärrstorp church, on the church yard north side of the church	KP659552	no	no
Pti363KP659555	*P. tiliacea*	F3	Pti11JF756989	Iran: Azarbaijan, East, Kaleybar county, on the way towards Aynaloo-Vaygan, ca. 5 km SE Vaygan, beside the main road	KP659555	no	no
Pti364KP659556	*P. tiliacea*	F3	Pti11JF756989	Iran: Azarbaijan, East, Kaleybar county, on the way towards Aynaloo-Vaygan, ca. 5 km SE Vaygan, beside the main road	KP659556	no	no
Pti365KP659557	*P. tiliacea*	F3	Pti11JF756989	Iran: Azarbaijan, East, Kaleybar county, on the way towards Aynaloo-Vaygan, ca. 5 km SE Vaygan, beside the main road	KP659557	no	no
Pti366KP659558	*P. tiliacea*	F3	Pti11JF756989	Iran: Azarbaijan, East, Kaleybar county, on the way towards Aynaloo-Vaygan, ca. 5 km SE Vaygan, beside the main road	KP659558	no	no
Pti367KP659559	*P. tiliacea*	F3	Pti11JF756989	Iran: Azarbaijan, East, Kaleybar county, on the way towards Aynaloo-Vaygan, ca. 5 km SE Vaygan, beside the main road	KP659559	no	no
Pti368KP659560	*P. tiliacea*	F3	Pti11JF756989	Iran: Azarbaijan, East, Kaleybar county, on the way towards Aynaloo-Vaygan, ca. 5 km SE Vaygan, beside the main road	KP659560	no	no
Pti7356	*P. tiliacea*	F3	Pti11JF756989	Switzerland: Bourg-Saint-Pierre, scenic viewpoint (bench)	PZ291023	yes	no
Pti7358	*P. tiliacea*	F3	Pti11JF756989	Switzerland: Bourg-Saint-Pierre, scenic viewpoint (bench)	PZ291024	yes	no
Pti7409	*P. tiliacea*	F3	Pti11JF756989	Switzerland: Collonges, Plex, Plex parking	PZ291025	yes	no
Pti7412	*P. tiliacea*	F3	Pti11JF756989	Switzerland: Bourg-Saint-Pierre, Forêt des Trones	PZ291026	yes	no
Pti7413	*P. tiliacea*	F3	Pti11JF756989	Switzerland: Bourg-Saint-Pierre, scenic viewpoint (bench)	PZ291027	yes	no
Pti7415	*P. tiliacea*	F3	Pti11JF756989	Switzerland: Bourg-Saint-Pierre, Parking	PZ291028	yes	no
Pti21JX466324	*P. tiliacea*	F3	Pti21JX466324	France: Cordes, Château de Cordes	JX466324	no	yes
Pti24JX466130	*P. tiliacea*	F3	Pti24JX466130	Spain: Tenerife, La Escalona, rocky outcrops of the ravine	JX466130	no	yes
Pti43JX466149	*P. tiliacea*	F3	Pti43JX466149	Spain: Tenerife, La Escalona, rocky outcrops of the ravine	JX466149	no	yes
Pti8JF756983	*P. tiliacea*	F3	Pti43JX466149	Spain: Gran Canaria, Galdar, between Saucillo and San José de Callideros	JF756983	no	no
Pti44JX466150	*P. tiliacea*	F3	Pti43JX466149	Spain: Tenerife, La Escalona, rocky outcrops of the ravine	JX466150	no	no
Pti45JX466151	*P. tiliacea*	F3	Pti43JX466149	Spain: Tenerife, La Escalona, rocky outcrops of the ravine	JX466151	no	no
Pti53JX466178	*P. tiliacea*	F3	Pti43JX466149	Spain: Gran Canaria, Vega de San Mateo to Tenteniguada	JX466178	no	no
Pti54JX466179	*P. tiliacea*	F3	Pti43JX466149	Spain: Gran Canaria, Vega de San Mateo to Tenteniguada	JX466179	no	no
Pti55JX466180	*P. tiliacea*	F3	Pti43JX466149	Spain: Gran Canaria, Vega de San Mateo to Tenteniguada	JX466180	no	no
Pti56JX466181	*P. tiliacea*	F3	Pti43JX466149	Spain: Gran Canaria, Vega de San Mateo to Tenteniguada	JX466181	no	no
Pti57JX466182	*P. tiliacea*	F3	Pti43JX466149	Spain: Gran Canaria, Vega de San Mateo to Tenteniguada	JX466182	no	no

Sequence code	Species (this study)	Clade/subclade	Haplotype representative	Locality	GenBank	Newly generated	Haplotype in Figure 1
Pti58JX466183	*P. tiliacea*	F3	Pti43JX466149	Spain: Gran Canaria, Vega de San Mateo to Tenteniguada	JX466183	no	no
Pti59JX466184	*P. tiliacea*	F3	Pti43JX466149	Spain: Gran Canaria, Vega de San Mateo to Tenteniguada	JX466184	no	no
Pti60JX466185	*P. tiliacea*	F3	Pti43JX466149	Spain: Gran Canaria, Vega de San Mateo to Tenteniguada	JX466185	no	no
Pti62JX466187	*P. tiliacea*	F3	Pti43JX466149	Spain: Gran Canaria, Galdar, between Saucillo and San José de Callideros	JX466187	no	no
Pti63JX466188	*P. tiliacea*	F3	Pti43JX466149	Spain: Gran Canaria, Galdar, between Saucillo and San José de Callideros	JX466188	no	no
Pti64JX466189	*P. tiliacea*	F3	Pti43JX466149	Spain: Gran Canaria, Galdar, between Saucillo and San José de Callideros	JX466189	no	no
Pti66JX466191	*P. tiliacea*	F3	Pti43JX466149	Spain: Gran Canaria, Galdar, between Saucillo and San José de Callideros	JX466191	no	no
Pti67JX466192	*P. tiliacea*	F3	Pti43JX466149	Spain: Gran Canaria, Galdar, between Saucillo and San José de Callideros	JX466192	no	no
Pti68JX466193	*P. tiliacea*	F3	Pti43JX466149	Spain: Gran Canaria, Galdar, between Saucillo and San José de Callideros	JX466193	no	no
Pti69JX466194	*P. tiliacea*	F3	Pti43JX466149	Spain: Gran Canaria, Galdar, between Saucillo and San José de Callideros	JX466194	no	no
Pti70JX466195	*P. tiliacea*	F3	Pti43JX466149	Spain: Gran Canaria, Galdar, between Saucillo and San José de Callideros	JX466195	no	no
Pti90JX466196	*P. tiliacea*	F3	Pti43JX466149	Morocco: Rabat, Maamora	JX466196	no	no
Pti94JX466199	*P. tiliacea*	F3	Pti94JX466199	Morocco: Ifrane, Forêt Sidi	JX466199	no	yes
Pti112JX466218	*P. tiliacea*	F3	Pti112JX466218	Spain: Cádiz, La Sauceda	JX466218	no	yes
Pti137JX466242	*P. tiliacea*	F3	Pti137JX466242	Spain: Cáceres, Cuacos de Yuste to Garganta la Olla Road	JX466242	no	yes
Pti4JF756984	*P. tiliacea*	F3	Pti137JX466242	Spain: Gran Canaria, above Valleseco, in cross of San Mateo and Artenara	JF56984	no	no
Pti52JX466177	*P. tiliacea*	F3	Pti137JX466242	Spain: Gran Canaria, Vega de San Mateo to Tenteniguada	JX466177	no	no
Pti61JX466186	*P. tiliacea*	F3	Pti137JX466242	Spain: Gran Canaria, Vega de San Mateo to Tenteniguada	JX466186	no	no
Pti77JX466164	*P. tiliacea*	F3	Pti137JX466242	Spain: Gran Canaria, Degollada de Becerra	JX466164	no	no
Pti84JX466171	*P. tiliacea*	F3	Pti137JX466242	Spain: Gran Canaria, above Valleseco, in cross of San Mateo and Antenara GC-21 / GC-231	JX466171	no	no
Pti156JX466258	*P. tiliacea*	F3	Pti156JX466258	Spain: Madrid, El Pardo	JX466258	no	yes
Pti170JX466269	*P. tiliacea*	F3	Pti170JX466269	Spain: Segovia, Enebral de Moral de Hornuez	JX466269	no	yes
Pti201JX466315	*P. tiliacea*	F3	Pti201JX466315	Italy: Sardegna, Bosa, Badde Orca	JX466315	no	yes
Pti243JX466328	*P. tiliacea*	F3	Pti243JX466328	Switzerland: Neuchatel, Cantón NE Neuchatel, Pierre-à-Bot	JX466328	no	yes
Pti250JX466335	*P. tiliacea*	F3	Pti243JX466328	Switzerland: Neuchatel, Cantón NE Neuchatel, Pierre-à-Bot	JX466335	no	no
Pti267JX466397	*P. tiliacea*	F3	Pti243JX466328	Austria: Salzburgo, Mittersill	JX466397	no	no
Pti278JX466408	*P. tiliacea*	F3	Pti243JX466328	Germany: Bad Reichenhall	JX466408	no	no
Pti279JX466409	*P. tiliacea*	F3	Pti243JX466328	Germany: Bad Reichenhall	JX466409	no	no
Pti281JX466411	*P. tiliacea*	F3	Pti243JX466328	Germany: Bad Reichenhall	JX466411	no	no
Pti295JX466463	*P. tiliacea*	F3	Pti243JX466328	Sweden: Uppland, Uppsala, Uppsala University Botanical Garden	JX466463	no	no
Pti296JX466464	*P. tiliacea*	F3	Pti243JX466328	Sweden: Uppland, Uppsala, Uppsala University Botanical Garden	JX466464	no	no
Pti376MK812259	*P. tiliacea*	F3	Pti243JX466328	Sweden: Sodermanland, Stora Malm church	MK812259	no	no
Pti379MK629945	*P. tiliacea*	F3	Pti243JX466328	China	MK629945	no	no
Pti269JX466399	*P. tiliacea*	F3	Pti269JX466399	Austria: Salzburgo, Mittersill	JX466399	no	yes
Pti271JX466401	*P. tiliacea*	F3	Pti269JX466399	Austria: Salzburgo, Mittersill	JX466401	no	no
Pti273JX466403	*P. tiliacea*	F3	Pti269JX466399	Austria: Salzburgo, Mittersill	JX466403	no	no
Pti276JX466406	*P. tiliacea*	F3	Pti269JX466399	Germany: Bad Reichenhall	JX466406	no	no
Pti284JX466416	*P. tiliacea*	F3	Pti269JX466399	Germany: Bavaria, Oberbayern, Kreis Freising, Aster Moos E Thurnsberg	JX466416	no	no
Pti372MK812562	*P. tiliacea*	F3	Pti269JX466399	Norway: Akershus, Frogn, Haoya, Haoybukta	MK812562	no	no
Pti374MK812472	*P. tiliacea*	F3	Pti269JX466399	Norway: Oslo, Malmoya	MK812472	no	no
Pti378MK811628	*P. tiliacea*	F3	Pti269JX466399	Norway: Oslo, Ostensjo farm	MK811628	no	no
Pti286JX466443	*P. tiliacea*	F3	Pti286JX466443	United Kingdom: Wales, road A5, between Rhydlydan and Glasfryn	JX466443	no	yes

Sequence code	Species (this study)	Clade/subclade	Haplotype representative	Locality	GenBank	Newly generated	Haplotype in Figure 1
Pti318JX466434	*P. tiliacea*	F3	Pti286JX466443	Turkey: Eskisehir, Sivirihisar Mountains	JX466434	no	no
Pti319JX466435	*P. tiliacea*	F3	Pti286JX466443	Turkey: Eskisehir, Sivirihisar Mountains	JX466435	no	no
Pti322JX466438	*P. tiliacea*	F3	Pti286JX466443	Turkey: Eskisehir, Sivirihisar Mountains	JX466438	no	no
Pti380KX132912	*P. tiliacea*	F3	Pti286JX466443	Switzerland: Valais	KX132912	no	no
Pti315JX466431	*P. tiliacea*	F3	Pti315JX466431	Turkey: Eskisehir, Nort of Asagi Caglan village	JX466431	no	yes
Pti349KP659541	*P. tiliacea*	F3	Pti315JX466431	Slovakia: Trnava Region, Smolenice, road from Smolenicky Zámok castle, towards Smolenice, next to a small reservoir	KP659541	no	no
Pti321JX466437	*P. tiliacea*	F3	Pti321JX466437	Turkey: Eskisehir, Sivirihisar Mountains	JX466437	no	yes
Pti362KP659554	*P. tiliacea*	F3	Pti362KP659554	Iran: Azarbaijan, East, Kaleybar county, on the way towards Aynaloo-Vaygan, ca. 5 km SE Vaygan, beside the main road	KP659554	no	yes
Pti7318	*P. tiliacea*	F3	Pti7318	Switzerland: Bourg-Saint-Pierre, scenic viewpoint (bench)	PZ291029	yes	yes
Pti7319	*P. tiliacea*	F3	Pti7318	Switzerland: Bourg-Saint-Pierre, Forêt des Trones	PZ291030	yes	no
Pti7327	*P. tiliacea*	F3	Pti7318	Switzerland: Martigny, Fully, route des Follateres, about 1926 Fully	PZ291031	yes	no
Pti7341	*P. tiliacea*	F3	Pti7318	Switzerland: Martigny, Fully, route des Follateres, about 1926 Fully	PZ291036	yes	no
Pti7349	*P. tiliacea*	F3	Pti7318	Switzerland: Bourg-Saint-Pierre, Forêt des Trones	PZ291037	yes	no
Pti7350	*P. tiliacea*	F3	Pti7318	Switzerland: Bourg-Saint-Pierre, scenic viewpoint (bench)	PZ291038	yes	no
Pti7351	*P. tiliacea*	F3	Pti7318	Switzerland: Bourg-Saint-Pierre, scenic viewpoint (bench)	PZ291039	yes	no
Pti7360	*P. tiliacea*	F3	Pti7318	Switzerland: Bourg-Saint-Pierre, scenic viewpoint (bench)	PZ291032	yes	no
Pti7361	*P. tiliacea*	F3	Pti7318	Switzerland: Bourg-Saint-Pierre, scenic viewpoint (bench)	PZ291033	yes	no
Pti7332	*P. tiliacea*	F3	Pti7332	Switzerland: Bourg-Saint-Pierre, scenic viewpoint (bench)	PZ291034	yes	yes
Pti7407	*P. tiliacea*	F3	Pti7332	Switzerland: Bourg-Saint-Pierre, Forêt des Trones	PZ291035	yes	no
Pti7398	*P. tiliacea*	F3	Pti7398	Switzerland: Martigny, Fully, route des Follateres, about 1926 Fully	PZ291040	yes	yes
Pya1GU979823	*P. yalungana*	A1	Pya1GU979823	USA: Alaska, Denali National Park, Quartz Lake State Recreation Area	GU979823	no	yes
Pya2GU979824	*P. yalungana*	A1	Pya1GU979823	USA: Alaska, Denali National Park, Quartz Lake State Recreation Area	GU979824	no	no
Pya3GU979821	*P. yalungana*	A1	Pya3GU979821	Russia: Buryatia Republic, Baikalskii Zapovednik Nature Reserve, along Pereemnaya River	GU979821	no	yes
MyirrKY128190				Japan: Yamanashi, Honshu, Prov. Of Kai, Nishizawa Valley, Mitomi-Kawaura, Yamanashi-city	KY128190	no	no
MymeAY611102				China: JianChuan County, Prov. of Yunnan	AY611102	no	no

Table S2

Table S2. Complete partitions recovered by ABGD and ASAP, both for the 749 ingroup specimens and for the 469 specimens of the *P. tiliacea* aggregate analyzed separately. A dash indicates that the specimen was not included in the analysis of the aggregate alone.

Sequence code	Species (current)	Clade/subclade	ASAP_ all_ rank1_ 8	ASAP_ all_ rank2_ 9	ABGD_ all_ 10	ASAP_ agg_ rank2_ 7	ABGD_ agg_ 42
Pat2JX466474	*P. atricha*	E1	1	1	1	-	-
Pat4EU520312	*P. atricha*	E1	1	1	1	-	-
Pat5EU520313	*P. atricha*	E1	1	1	1	-	-
Pat6EU520314	*P. atricha*	E1	1	1	1	-	-
Pat7330	*P. atricha*	E1	1	1	1	-	-
Pat7331	*P. atricha*	E1	1	1	1	-	-
Pat7336	*P. atricha*	E1	1	1	1	-	-
Pat7337	*P. atricha*	E1	1	1	1	-	-
Pat7338	*P. atricha*	E1	1	1	1	-	-
Pat7346	*P. atricha*	E1	1	1	1	-	-
Pat7347	*P. atricha*	E1	1	1	1	-	-
Pat7348	*P. atricha*	E1	1	1	1	-	-
Pat7359	*P. atricha*	E1	1	1	1	-	-
Pat7369	*P. atricha*	E1	1	1	1	-	-
Pat7370	*P. atricha*	E1	1	1	1	-	-
Pat7371	*P. atricha*	E1	1	1	1	-	-
Pat7372	*P. atricha*	E1	1	1	1	-	-
Pat7373	*P. atricha*	E1	1	1	1	-	-
Pat7374	*P. atricha*	E1	1	1	1	-	-
Pat7375	*P. atricha*	E1	1	1	1	-	-
Pat7376	*P. atricha*	E1	1	1	1	-	-
Pat7377	*P. atricha*	E1	1	1	1	-	-
Pat7384	*P. atricha*	E1	1	1	1	-	-
Pat7388	*P. atricha*	E1	1	1	1	-	-
Pat7389	*P. atricha*	E1	1	1	1	-	-
Pat7390	*P. atricha*	E1	1	1	1	-	-
Pat7393	*P. atricha*	E1	1	1	1	-	-
Pat7394	*P. atricha*	E1	1	1	1	-	-
Pat7395	*P. atricha*	E1	1	1	1	-	-
Pat7397	*P. atricha*	E1	1	1	1	-	-
Pat7400	*P. atricha*	E1	1	1	1	-	-
Pat7401	*P. atricha*	E1	1	1	1	-	-
Pat7402	*P. atricha*	E1	1	1	1	-	-
Pat7403	*P. atricha*	E1	1	1	1	-	-
Pat7406	*P. atricha*	E1	1	1	1	-	-

Sequence code	Species (current)	Clade/subclade	ASAP_ all_ rank1_ 8	ASAP_ all_ rank2_ 9	ABGD_ all_ 10	ASAP_ agg_ rank2_ 7	ABGD_ agg_ 42
Pat7EU520315	*P. atricha*	E1	1	1	1	-	-
Pat1JX466473	*P. atricha*	E2	2	2	2	-	-
Pat3JX466475	*P. atricha*	E2	2	2	2	-	-
Pca10JF756993	*P. carporrhizans*	E3	2	3	3	-	-
Pca11KX457684	*P. carporrhizans*	E3	2	3	3	-	-
Pca12KM357893	*P. carporrhizans*	E3	2	3	3	-	-
Pca13KM357892	*P. carporrhizans*	E3	2	3	3	-	-
Pca1DQ273849	*P. carporrhizans*	E3	2	3	3	-	-
Pca2DQ273850	*P. carporrhizans*	E3	2	3	3	-	-
Pca3350	*P. carporrhizans*	E3	2	3	3	-	-
Pca3364	*P. carporrhizans*	E3	2	3	3	-	-
Pca3365	*P. carporrhizans*	E3	2	3	3	-	-
Pca3366	*P. carporrhizans*	E3	2	3	3	-	-
Pca3367	*P. carporrhizans*	E3	2	3	3	-	-
Pca3418	*P. carporrhizans*	E3	2	3	3	-	-
Pca3419	*P. carporrhizans*	E3	2	3	3	-	-
Pca3421	*P. carporrhizans*	E3	2	3	3	-	-
Pca3423	*P. carporrhizans*	E3	2	3	3	-	-
Pca3424	*P. carporrhizans*	E3	2	3	3	-	-
Pca3425	*P. carporrhizans*	E3	2	3	3	-	-
Pca3426	*P. carporrhizans*	E3	2	3	3	-	-
Pca3427	*P. carporrhizans*	E3	2	3	3	-	-
Pca3428	*P. carporrhizans*	E3	2	3	3	-	-
Pca3429	*P. carporrhizans*	E3	2	3	3	-	-
Pca3430	*P. carporrhizans*	E3	2	3	3	-	-
Pca3431	*P. carporrhizans*	E3	2	3	3	-	-
Pca3432	*P. carporrhizans*	E3	2	3	3	-	-
Pca3433	*P. carporrhizans*	E3	2	3	3	-	-
Pca3434	*P. carporrhizans*	E3	2	3	3	-	-
Pca3435	*P. carporrhizans*	E3	2	3	3	-	-
Pca3436	*P. carporrhizans*	E3	2	3	3	-	-
Pca3437	*P. carporrhizans*	E3	2	3	3	-	-
Pca3618	*P. carporrhizans*	E3	2	3	3	-	-
Pca3619	*P. carporrhizans*	E3	2	3	3	-	-
Pca3620	*P. carporrhizans*	E3	2	3	3	-	-
Pca3621	*P. carporrhizans*	E3	2	3	3	-	-
Pca3622	*P. carporrhizans*	E3	2	3	3	-	-
Pca3623	*P. carporrhizans*	E3	2	3	3	-	-
Pca3624	*P. carporrhizans*	E3	2	3	3	-	-
Pca3625	*P. carporrhizans*	E3	2	3	3	-	-

Sequence code	Species (current)	Clade/subclade	ASAP_ all_ rank1_ 8	ASAP_ all_ rank2_ 9	ABGD_ all_ 10	ASAP_ agg_ rank2_ 7	ABGD_ agg_ 42
Pca3626	*P. carporrhizans*	E3	2	3	3	-	-
Pca3627	*P. carporrhizans*	E3	2	3	3	-	-
Pca3628	*P. carporrhizans*	E3	2	3	3	-	-
Pca3629	*P. carporrhizans*	E3	2	3	3	-	-
Pca3630	*P. carporrhizans*	E3	2	3	3	-	-
Pca3631	*P. carporrhizans*	E3	2	3	3	-	-
Pca3636	*P. carporrhizans*	E3	2	3	3	-	-
Pca3637	*P. carporrhizans*	E3	2	3	3	-	-
Pca3638	*P. carporrhizans*	E3	2	3	3	-	-
Pca3639	*P. carporrhizans*	E3	2	3	3	-	-
Pca3641	*P. carporrhizans*	E3	2	3	3	-	-
Pca3642	*P. carporrhizans*	E3	2	3	3	-	-
Pca3645	*P. carporrhizans*	E3	2	3	3	-	-
Pca3646	*P. carporrhizans*	E3	2	3	3	-	-
Pca3647	*P. carporrhizans*	E3	2	3	3	-	-
Pca3648	*P. carporrhizans*	E3	2	3	3	-	-
Pca3651	*P. carporrhizans*	E3	2	3	3	-	-
Pca3652	*P. carporrhizans*	E3	2	3	3	-	-
Pca3653	*P. carporrhizans*	E3	2	3	3	-	-
Pca3654	*P. carporrhizans*	E3	2	3	3	-	-
Pca3655	*P. carporrhizans*	E3	2	3	3	-	-
Pca3656	*P. carporrhizans*	E3	2	3	3	-	-
Pca3657	*P. carporrhizans*	E3	2	3	3	-	-
Pca3658	*P. carporrhizans*	E3	2	3	3	-	-
Pca3659	*P. carporrhizans*	E3	2	3	3	-	-
Pca3660	*P. carporrhizans*	E3	2	3	3	-	-
Pca3661	*P. carporrhizans*	E3	2	3	3	-	-
Pca3662	*P. carporrhizans*	E3	2	3	3	-	-
Pca3663	*P. carporrhizans*	E3	2	3	3	-	-
Pca3664	*P. carporrhizans*	E3	2	3	3	-	-
Pca3668	*P. carporrhizans*	E3	2	3	3	-	-
Pca3983	*P. carporrhizans*	E3	2	3	3	-	-
Pca3984	*P. carporrhizans*	E3	2	3	3	-	-
Pca3985	*P. carporrhizans*	E3	2	3	3	-	-
Pca3DQ273851	*P. carporrhizans*	E3	2	3	3	-	-
Pca4011	*P. carporrhizans*	E3	2	3	3	-	-
Pca4012	*P. carporrhizans*	E3	2	3	3	-	-
Pca4013	*P. carporrhizans*	E3	2	3	3	-	-
Pca4014	*P. carporrhizans*	E3	2	3	3	-	-
Pca4015	*P. carporrhizans*	E3	2	3	3	-	-

Sequence code	Species (current)	Clade/subclade	ASAP_ all_ rank1_ 8	ASAP_ all_ rank2_ 9	ABGD_ all_ 10	ASAP_ agg_ rank2_ 7	ABGD_ agg_ 42
Pca4016	*P. carporrhizans*	E3	2	3	3	-	-
Pca4053	*P. carporrhizans*	E3	2	3	3	-	-
Pca4054	*P. carporrhizans*	E3	2	3	3	-	-
Pca4055	*P. carporrhizans*	E3	2	3	3	-	-
Pca4056	*P. carporrhizans*	E3	2	3	3	-	-
Pca4057	*P. carporrhizans*	E3	2	3	3	-	-
Pca4058	*P. carporrhizans*	E3	2	3	3	-	-
Pca4059	*P. carporrhizans*	E3	2	3	3	-	-
Pca4060	*P. carporrhizans*	E3	2	3	3	-	-
Pca4061	*P. carporrhizans*	E3	2	3	3	-	-
Pca4062	*P. carporrhizans*	E3	2	3	3	-	-
Pca4063	*P. carporrhizans*	E3	2	3	3	-	-
Pca4064	*P. carporrhizans*	E3	2	3	3	-	-
Pca4100	*P. carporrhizans*	E3	2	3	3	-	-
Pca4101	*P. carporrhizans*	E3	2	3	3	-	-
Pca4102	*P. carporrhizans*	E3	2	3	3	-	-
Pca4103	*P. carporrhizans*	E3	2	3	3	-	-
Pca4104	*P. carporrhizans*	E3	2	3	3	-	-
Pca4105	*P. carporrhizans*	E3	2	3	3	-	-
Pca4106	*P. carporrhizans*	E3	2	3	3	-	-
Pca4107	*P. carporrhizans*	E3	2	3	3	-	-
Pca4108	*P. carporrhizans*	E3	2	3	3	-	-
Pca4110	*P. carporrhizans*	E3	2	3	3	-	-
Pca4111	*P. carporrhizans*	E3	2	3	3	-	-
Pca4112	*P. carporrhizans*	E3	2	3	3	-	-
Pca4114	*P. carporrhizans*	E3	2	3	3	-	-
Pca4118	*P. carporrhizans*	E3	2	3	3	-	-
Pca4119	*P. carporrhizans*	E3	2	3	3	-	-
Pca4120	*P. carporrhizans*	E3	2	3	3	-	-
Pca4122	*P. carporrhizans*	E3	2	3	3	-	-
Pca4123	*P. carporrhizans*	E3	2	3	3	-	-
Pca4384	*P. carporrhizans*	E3	2	3	3	-	-
Pca4385	*P. carporrhizans*	E3	2	3	3	-	-
Pca4387	*P. carporrhizans*	E3	2	3	3	-	-
Pca4388	*P. carporrhizans*	E3	2	3	3	-	-
Pca4389	*P. carporrhizans*	E3	2	3	3	-	-
Pca4390	*P. carporrhizans*	E3	2	3	3	-	-
Pca4392	*P. carporrhizans*	E3	2	3	3	-	-
Pca4399	*P. carporrhizans*	E3	2	3	3	-	-
Pca4400	*P. carporrhizans*	E3	2	3	3	-	-

Sequence code	Species (current)	Clade/subclade	ASAP_ all_ rank1_ 8	ASAP_ all_ rank2_ 9	ABGD_ all_ 10	ASAP_ agg_ rank2_ 7	ABGD_ agg_ 42
Pca4401	*P. carporrhizans*	E3	2	3	3	-	-
Pca4405	*P. carporrhizans*	E3	2	3	3	-	-
Pca4406	*P. carporrhizans*	E3	2	3	3	-	-
Pca4411	*P. carporrhizans*	E3	2	3	3	-	-
Pca4416	*P. carporrhizans*	E3	2	3	3	-	-
Pca4417	*P. carporrhizans*	E3	2	3	3	-	-
Pca4421	*P. carporrhizans*	E3	2	3	3	-	-
Pca4492	*P. carporrhizans*	E3	2	3	3	-	-
Pca4493	*P. carporrhizans*	E3	2	3	3	-	-
Pca4494	*P. carporrhizans*	E3	2	3	3	-	-
Pca4495	*P. carporrhizans*	E3	2	3	3	-	-
Pca4496	*P. carporrhizans*	E3	2	3	3	-	-
Pca4497	*P. carporrhizans*	E3	2	3	3	-	-
Pca4498	*P. carporrhizans*	E3	2	3	3	-	-
Pca4502	*P. carporrhizans*	E3	2	3	3	-	-
Pca4503	*P. carporrhizans*	E3	2	3	3	-	-
Pca4504	*P. carporrhizans*	E3	2	3	3	-	-
Pca4506	*P. carporrhizans*	E3	2	3	3	-	-
Pca4507	*P. carporrhizans*	E3	2	3	3	-	-
Pca4508	*P. carporrhizans*	E3	2	3	3	-	-
Pca4509	*P. carporrhizans*	E3	2	3	3	-	-
Pca4511	*P. carporrhizans*	E3	2	3	3	-	-
Pca4512	*P. carporrhizans*	E3	2	3	3	-	-
Pca4513	*P. carporrhizans*	E3	2	3	3	-	-
Pca4514	*P. carporrhizans*	E3	2	3	3	-	-
Pca4515	*P. carporrhizans*	E3	2	3	3	-	-
Pca4516	*P. carporrhizans*	E3	2	3	3	-	-
Pca4517	*P. carporrhizans*	E3	2	3	3	-	-
Pca4518	*P. carporrhizans*	E3	2	3	3	-	-
Pca4519	*P. carporrhizans*	E3	2	3	3	-	-
Pca4520	*P. carporrhizans*	E3	2	3	3	-	-
Pca4521	*P. carporrhizans*	E3	2	3	3	-	-
Pca4522	*P. carporrhizans*	E3	2	3	3	-	-
Pca4523	*P. carporrhizans*	E3	2	3	3	-	-
Pca4524	*P. carporrhizans*	E3	2	3	3	-	-
Pca4525	*P. carporrhizans*	E3	2	3	3	-	-
Pca4526	*P. carporrhizans*	E3	2	3	3	-	-
Pca4527	*P. carporrhizans*	E3	2	3	3	-	-
Pca4528	*P. carporrhizans*	E3	2	3	3	-	-
Pca4529	*P. carporrhizans*	E3	2	3	3	-	-

Sequence code	Species (current)	Clade/subclade	ASAP_ all_ rank1_ 8	ASAP_ all_ rank2_ 9	ABGD_ all_ 10	ASAP_ agg_ rank2_ 7	ABGD_ agg_ 42
Pca4530	*P. carporrhizans*	E3	2	3	3	-	-
Pca4531	*P. carporrhizans*	E3	2	3	3	-	-
Pca4532	*P. carporrhizans*	E3	2	3	3	-	-
Pca4533	*P. carporrhizans*	E3	2	3	3	-	-
Pca4534	*P. carporrhizans*	E3	2	3	3	-	-
Pca4535	*P. carporrhizans*	E3	2	3	3	-	-
Pca4536	*P. carporrhizans*	E3	2	3	3	-	-
Pca4537	*P. carporrhizans*	E3	2	3	3	-	-
Pca4538	*P. carporrhizans*	E3	2	3	3	-	-
Pca4539	*P. carporrhizans*	E3	2	3	3	-	-
Pca4540	*P. carporrhizans*	E3	2	3	3	-	-
Pca4541	*P. carporrhizans*	E3	2	3	3	-	-
Pca4542	*P. carporrhizans*	E3	2	3	3	-	-
Pca4543	*P. carporrhizans*	E3	2	3	3	-	-
Pca4544	*P. carporrhizans*	E3	2	3	3	-	-
Pca4545	*P. carporrhizans*	E3	2	3	3	-	-
Pca4546	*P. carporrhizans*	E3	2	3	3	-	-
Pca4547	*P. carporrhizans*	E3	2	3	3	-	-
Pca4548	*P. carporrhizans*	E3	2	3	3	-	-
Pca4549	*P. carporrhizans*	E3	2	3	3	-	-
Pca4550	*P. carporrhizans*	E3	2	3	3	-	-
Pca4551	*P. carporrhizans*	E3	2	3	3	-	-
Pca4552	*P. carporrhizans*	E3	2	3	3	-	-
Pca4554	*P. carporrhizans*	E3	2	3	3	-	-
Pca4555	*P. carporrhizans*	E3	2	3	3	-	-
Pca4556	*P. carporrhizans*	E3	2	3	3	-	-
Pca4557	*P. carporrhizans*	E3	2	3	3	-	-
Pca4558	*P. carporrhizans*	E3	2	3	3	-	-
Pca4559	*P. carporrhizans*	E3	2	3	3	-	-
Pca4DQ273852	*P. carporrhizans*	E3	2	3	3	-	-
Pca5DQ273853	*P. carporrhizans*	E3	2	3	3	-	-
Pca6DQ273854	*P. carporrhizans*	E3	2	3	3	-	-
Pca7JF756995	*P. carporrhizans*	E3	2	3	3	-	-
Pca8JF756996	*P. carporrhizans*	E3	2	3	3	-	-
Pca9JF756994	*P. carporrhizans*	E3	2	3	3	-	-
Pqu13AY611105	*P. carporrhizans*	E3	2	3	3	-	-
Pcl237JX466355	*P. clandestina*	F2	3	4	4	1	1
Pcl254JX466382	*P. clandestina*	F2	3	4	4	1	2
Pcl259JX466389	*P. clandestina*	F2	3	4	4	1	1
Pcl260JX466390	*P. clandestina*	F2	3	4	4	1	1

Sequence code	Species (current)	Clade/subclade	ASAP_ all_ rank1_ 8	ASAP_ all_ rank2_ 9	ABGD_ all_ 10	ASAP_ agg_ rank2_ 7	ABGD_ agg_ 42
Pti180JX466287	*P. clandestina*	F2	3	4	4	1	1
Pti209JX466360	*P. clandestina*	F2	3	4	4	1	1
Pti210JX466361	*P. clandestina*	F2	3	4	4	1	1
Pti252JX466380	*P. clandestina*	F2	3	4	4	1	1
Pti255JX466383	*P. clandestina*	F2	3	4	4	1	1
Pti256JX466384	*P. clandestina*	F2	3	4	4	1	1
Pti257JX466385	*P. clandestina*	F2	3	4	4	1	2
Pti261JX466391	*P. clandestina*	F2	3	4	4	1	1
Pti262JX466392	*P. clandestina*	F2	3	4	4	1	1
Pti263JX466393	*P. clandestina*	F2	3	4	4	1	1
Pti264JX466394	*P. clandestina*	F2	3	4	4	1	1
Pti265JX466395	*P. clandestina*	F2	3	4	4	1	1
Pti266JX466396	*P. clandestina*	F2	3	4	4	1	1
Pti274JX466404	*P. clandestina*	F2	3	4	4	1	1
Pti306JX466419	*P. clandestina*	F2	3	4	4	1	3
Pti313JX466428	*P. clandestina*	F2	3	4	4	1	2
Pti317JX466433	*P. clandestina*	F2	3	4	4	1	1
Pti326JX466442	*P. clandestina*	F2	3	4	4	1	2
Pti342KP659534	*P. clandestina*	F2	3	4	4	1	1
Pti346KP659538	*P. clandestina*	F2	3	4	4	1	1
Pti351KP659543	*P. clandestina*	F2	3	4	4	1	2
Pti352KP659544	*P. clandestina*	F2	3	4	4	1	2
Pti353KP659545	*P. clandestina*	F2	3	4	4	1	2
Pti361KP659553	*P. clandestina*	F2	3	4	4	1	1
Pti369KP659561	*P. clandestina*	F2	3	4	4	1	2
Pti370KP659562	*P. clandestina*	F2	3	4	4	1	2
Pti373MK812477	*P. clandestina*	F2	3	4	4	1	3
Pti377MK811862	*P. clandestina*	F2	3	4	4	1	1
Pti381EU266085	*P. clandestina*	F2	3	4	4	1	3
Pti7320	*P. clandestina*	F2	3	4	4	2	4
Pti7326	*P. clandestina*	F2	3	4	4	2	5
Pti7357	*P. clandestina*	F2	3	4	4	1	1
Pti7383	*P. clandestina*	F2	3	4	4	1	1
Pti7411	*P. clandestina*	F2	3	4	4	2	6
Pat7385	*P. clandestina*	F4	3	4	4	3	7
Pat7396	*P. clandestina*	F4	3	4	4	3	7
Pcl132JX466237	*P. clandestina*	F4	3	4	4	3	8
Pcl25JX466131	*P. clandestina*	F4	3	4	4	3	7
Pcl285JX466417	*P. clandestina*	F4	3	4	4	3	8
Pcl288JX466445	*P. clandestina*	F4	3	4	4	3	7

Sequence code	Species (current)	Clade/subclade	ASAP_ all_ rank1_ 8	ASAP_ all_ rank2_ 9	ABGD_ all_ 10	ASAP_ agg_ rank2_ 7	ABGD_ agg_ 42
Pcl289JX466446	*P. clandestina*	F4	3	4	4	3	7
Pcl323JX466439	*P. clandestina*	F4	3	4	4	3	7
Pcl325JX466441	*P. clandestina*	F4	3	4	4	3	7
Pcl93JX466198	*P. clandestina*	F4	3	4	4	3	8
Pcl98JX466203	*P. clandestina*	F4	3	4	4	3	8
Pcl99JX466204	*P. clandestina*	F4	3	4	4	3	8
Pti100JX466205	*P. clandestina*	F4	3	4	4	3	7
Pti101JX466207	*P. clandestina*	F4	3	4	4	3	9
Pti103JX466209	*P. clandestina*	F4	3	4	4	3	7
Pti107JX466213	*P. clandestina*	F4	3	4	4	3	8
Pti108JX466214	*P. clandestina*	F4	3	4	4	3	7
Pti113JX466219	*P. clandestina*	F4	3	4	4	3	7
Pti114JX466220	*P. clandestina*	F4	3	4	4	3	8
Pti115JX466221	*P. clandestina*	F4	3	4	4	3	7
Pti117JX466223	*P. clandestina*	F4	3	4	4	3	7
Pti118JX466224	*P. clandestina*	F4	3	4	4	3	7
Pti119JX466225	*P. clandestina*	F4	3	4	4	3	7
Pti120JX466226	*P. clandestina*	F4	3	4	4	3	7
Pti121JX466227	*P. clandestina*	F4	3	4	4	3	7
Pti122JX466228	*P. clandestina*	F4	3	4	4	3	7
Pti123JX466229	*P. clandestina*	F4	3	4	4	3	7
Pti124JX466230	*P. clandestina*	F4	3	4	4	3	7
Pti125JX466231	*P. clandestina*	F4	3	4	4	3	7
Pti126JX466232	*P. clandestina*	F4	3	4	4	3	8
Pti127JX466256	*P. clandestina*	F4	3	4	4	3	8
Pti128JX466233	*P. clandestina*	F4	3	4	4	3	8
Pti129JX466234	*P. clandestina*	F4	3	4	4	3	8
Pti130JX466235	*P. clandestina*	F4	3	4	4	3	10
Pti131JX466236	*P. clandestina*	F4	3	4	4	3	8
Pti134JX466239	*P. clandestina*	F4	3	4	4	3	11
Pti136JX466241	*P. clandestina*	F4	3	4	4	3	8
Pti138JX466243	*P. clandestina*	F4	3	4	4	3	7
Pti139JX466244	*P. clandestina*	F4	3	4	4	3	7
Pti13JF756992	*P. clandestina*	F4	3	4	4	3	7
Pti140JX466245	*P. clandestina*	F4	3	4	4	3	7
Pti141JX466246	*P. clandestina*	F4	3	4	4	3	7
Pti142JX466247	*P. clandestina*	F4	3	4	4	3	7
Pti143JX466248	*P. clandestina*	F4	3	4	4	3	7
Pti144JX466249	*P. clandestina*	F4	3	4	4	3	7
Pti145JX466250	*P. clandestina*	F4	3	4	4	3	7

Sequence code	Species (current)	Clade/subclade	ASAP_ all_ rank1_ 8	ASAP_ all_ rank2_ 9	ABGD_ all_ 10	ASAP_ agg_ rank2_ 7	ABGD_ agg_ 42
Pti146JX466251	*P. clandestina*	F4	3	4	4	3	7
Pti147JX466252	*P. clandestina*	F4	3	4	4	3	7
Pti148JX466253	*P. clandestina*	F4	3	4	4	3	8
Pti149JX466254	*P. clandestina*	F4	3	4	4	3	7
Pti14JF756981	*P. clandestina*	F4	3	4	4	3	8
Pti150JX466255	*P. clandestina*	F4	3	4	4	3	8
Pti152JX466260	*P. clandestina*	F4	3	4	4	3	7
Pti157JX466259	*P. clandestina*	F4	3	4	4	3	7
Pti158JX466264	*P. clandestina*	F4	3	4	4	3	8
Pti159JX466266	*P. clandestina*	F4	3	4	4	3	7
Pti15JF756980	*P. clandestina*	F4	3	4	4	3	7
Pti160JX466267	*P. clandestina*	F4	3	4	4	3	7
Pti161JX466268	*P. clandestina*	F4	3	4	4	3	7
Pti162JX466283	*P. clandestina*	F4	3	4	4	3	7
Pti163JX466284	*P. clandestina*	F4	3	4	4	3	7
Pti164JX466285	*P. clandestina*	F4	3	4	4	3	7
Pti165JX466286	*P. clandestina*	F4	3	4	4	3	7
Pti166JX466279	*P. clandestina*	F4	3	4	4	3	7
Pti167JX466280	*P. clandestina*	F4	3	4	4	3	7
Pti168JX466281	*P. clandestina*	F4	3	4	4	3	7
Pti169JX466282	*P. clandestina*	F4	3	4	4	3	8
Pti17JF756988	*P. clandestina*	F4	3	4	4	3	8
Pti19AY581084	*P. clandestina*	F4	3	4	4	3	7
Pti20DQ273861	*P. clandestina*	F4	3	4	4	3	7
Pti238JX466323	*P. clandestina*	F4	3	4	4	3	7
Pti23JX466265	*P. clandestina*	F4	3	4	4	3	7
Pti26JX466132	*P. clandestina*	F4	3	4	4	3	8
Pti277JX466407	*P. clandestina*	F4	3	4	4	3	8
Pti27JX466133	*P. clandestina*	F4	3	4	4	3	7
Pti280JX466410	*P. clandestina*	F4	3	4	4	3	7
Pti287JX466444	*P. clandestina*	F4	3	4	4	3	7
Pti28JX466134	*P. clandestina*	F4	3	4	4	3	7
Pti29JX466135	*P. clandestina*	F4	3	4	4	3	8
Pti305JX466418	*P. clandestina*	F4	3	4	4	3	8
Pti30JX466136	*P. clandestina*	F4	3	4	4	3	7
Pti311JX466425	*P. clandestina*	F4	3	4	4	3	7
Pti31JX466137	*P. clandestina*	F4	3	4	4	3	7
Pti32JX466138	*P. clandestina*	F4	3	4	4	3	7
Pti344KP659536	*P. clandestina*	F4	3	4	4	3	12
Pti371MK812693	*P. clandestina*	F4	3	4	4	3	7

Sequence code	Species (current)	Clade/subclade	ASAP_ all_ rank1_ 8	ASAP_ all_ rank2_ 9	ABGD_ all_ 10	ASAP_ agg_ rank2_ 7	ABGD_ agg_ 42
Pti375MK812323	*P. clandestina*	F4	3	4	4	3	7
Pti5JF756979	*P. clandestina*	F4	3	4	4	3	8
Pti6JF756986	*P. clandestina*	F4	3	4	4	3	13
Pti71JX466158	*P. clandestina*	F4	3	4	4	3	8
Pti72JX466159	*P. clandestina*	F4	3	4	4	3	7
Pti73JX466160	*P. clandestina*	F4	3	4	4	3	8
Pti7405	*P. clandestina*	F4	3	4	4	3	7
Pti7408	*P. clandestina*	F4	3	4	4	3	8
Pti7414	*P. clandestina*	F4	3	4	4	3	7
Pti74JX466161	*P. clandestina*	F4	3	4	4	3	7
Pti75JX466162	*P. clandestina*	F4	3	4	4	3	7
Pti76JX466163	*P. clandestina*	F4	3	4	4	3	8
Pti78JX466165	*P. clandestina*	F4	3	4	4	3	8
Pti79JX466166	*P. clandestina*	F4	3	4	4	3	8
Pti7JF756976	*P. clandestina*	F4	3	4	4	3	7
Pti80JX466167	*P. clandestina*	F4	3	4	4	3	8
Pti82JX466169	*P. clandestina*	F4	3	4	4	3	8
Pti83JX466170	*P. clandestina*	F4	3	4	4	3	8
Pti85JX466172	*P. clandestina*	F4	3	4	4	3	7
Pti86JX466173	*P. clandestina*	F4	3	4	4	3	8
Pti87JX466174	*P. clandestina*	F4	3	4	4	3	7
Pti88JX466175	*P. clandestina*	F4	3	4	4	3	8
Pti89JX466176	*P. clandestina*	F4	3	4	4	3	8
Pti92JX466197	*P. clandestina*	F4	3	4	4	3	7
Pti95JX466200	*P. clandestina*	F4	3	4	4	3	7
Pti96JX466201	*P. clandestina*	F4	3	4	4	3	8
Pti97JX466202	*P. clandestina*	F4	3	4	4	3	13
Pti9JF756978	*P. clandestina*	F4	3	4	4	3	8
PtiI1954	*P. clandestina*	F4	3	4	4	3	7
PtiI1992	*P. clandestina*	F4	3	4	4	3	7
Pco1023ITS	*P. coleae*	D	4	5	5	-	-
Pco1024ITS	*P. coleae*	D	4	5	5	-	-
Pco1026ITS	*P. coleae*	D	4	5	5	-	-
Pco1027ITS	*P. coleae*	D	4	5	5	-	-
Pco1028ITS	*P. coleae*	D	4	5	5	-	-
Pco1029ITS	*P. coleae*	D	4	5	5	-	-
Pco1030ITS	*P. coleae*	D	4	5	5	-	-
Pco1031ITS	*P. coleae*	D	4	5	5	-	-
Pco1032ITS	*P. coleae*	D	4	5	5	-	-
Pco1033ITS	*P. coleae*	D	4	5	5	-	-

Sequence code	Species (current)	Clade/subclade	ASAP_ all_ rank1_ 8	ASAP_ all_ rank2_ 9	ABGD_ all_ 10	ASAP_ agg_ rank2_ 7	ABGD_ agg_ 42
Pco1DQ273855	*P. coleae*	D	4	5	5	-	-
Pco2DQ273856	*P. coleae*	D	4	5	5	-	-
Pco3DQ273857	*P. coleae*	D	4	5	5	-	-
Pco4DQ273858	*P. coleae*	D	4	5	5	-	-
Pco5JF756966	*P. coleae*	D	4	5	5	-	-
Pco6JF756967	*P. coleae*	D	4	5	5	-	-
Pco7JF756968	*P. coleae*	D	4	5	5	-	-
Pco8GU979822	*P. coleae*	D	4	5	5	-	-
Pcr1JF756970	*P. cryptotiliacea*	B	5	6	6	-	-
Pcr2JF756975	*P. cryptotiliacea*	B	5	6	6	-	-
Pcr3JF756974	*P. cryptotiliacea*	B	5	6	6	-	-
Pcr4JF756972	*P. cryptotiliacea*	B	5	6	6	-	-
Pcr5JF756969	*P. cryptotiliacea*	B	5	6	6	-	-
Pcr6988	*P. cryptotiliacea*	B	5	6	7	-	-
Pcr6JF756973	*P. cryptotiliacea*	B	5	6	6	-	-
Pcr7JF756971	*P. cryptotiliacea*	B	5	6	6	-	-
Pcr8JX466476	*P. cryptotiliacea*	B	5	6	6	-	-
Pcr9JX466477	*P. cryptotiliacea*	B	5	6	6	-	-
Pme192JX466306	*P. mediterranea*	F5	3	4	4	3	14
Pme194JX466308	*P. mediterranea*	F5	3	4	4	3	15
Pme197JX466311	*P. mediterranea*	F5	3	4	4	3	16
Pme198JX466312	*P. mediterranea*	F5	3	4	4	3	16
Pme221JX466348	*P. mediterranea*	F5	3	4	4	3	14
Pme225JX466352	*P. mediterranea*	F5	3	4	4	3	14
Pme39JX466145	*P. mediterranea*	F5	3	4	4	3	16
Pme41JX466147	*P. mediterranea*	F5	3	4	4	3	16
Pti102JX466208	*P. mediterranea*	F5	3	4	4	3	16
Pti104JX466210	*P. mediterranea*	F5	3	4	4	3	16
Pti105JX466211	*P. mediterranea*	F5	3	4	4	3	16
Pti106JX466212	*P. mediterranea*	F5	3	4	4	3	17
Pti109JX466215	*P. mediterranea*	F5	3	4	4	3	16
Pti10JF756985	*P. mediterranea*	F5	3	4	4	3	16
Pti110JX466216	*P. mediterranea*	F5	3	4	4	3	16
Pti111JX466217	*P. mediterranea*	F5	3	4	4	3	16
Pti16JF756990	*P. mediterranea*	F5	3	4	4	3	16
Pti183JX466297	*P. mediterranea*	F5	3	4	4	3	16
Pti184JX466298	*P. mediterranea*	F5	3	4	4	3	16
Pti185JX466299	*P. mediterranea*	F5	3	4	4	3	16
Pti186JX466300	*P. mediterranea*	F5	3	4	4	3	16
Pti187JX466301	*P. mediterranea*	F5	3	4	4	3	16

Sequence code	Species (current)	Clade/subclade	ASAP_ all_ rank1_ 8	ASAP_ all_ rank2_ 9	ABGD_ all_ 10	ASAP_ agg_ rank2_ 7	ABGD_ agg_ 42
Pti188JX466302	*P. mediterranea*	F5	3	4	4	3	16
Pti189JX466303	*P. mediterranea*	F5	3	4	4	3	16
Pti190JX466304	*P. mediterranea*	F5	3	4	4	3	16
Pti191JX466305	*P. mediterranea*	F5	3	4	4	3	16
Pti193JX466307	*P. mediterranea*	F5	3	4	4	3	16
Pti195JX466309	*P. mediterranea*	F5	3	4	4	3	16
Pti196JX466310	*P. mediterranea*	F5	3	4	4	3	16
Pti199JX466313	*P. mediterranea*	F5	3	4	4	3	16
Pti1JF756977	*P. mediterranea*	F5	3	4	4	3	15
Pti200JX466314	*P. mediterranea*	F5	3	4	4	3	16
Pti202JX466316	*P. mediterranea*	F5	3	4	4	3	16
Pti203JX466317	*P. mediterranea*	F5	3	4	4	3	14
Pti204JX466318	*P. mediterranea*	F5	3	4	4	3	16
Pti205JX466319	*P. mediterranea*	F5	3	4	4	3	16
Pti206JX466321	*P. mediterranea*	F5	3	4	4	3	16
Pti208JX466322	*P. mediterranea*	F5	3	4	4	3	16
Pti211JX466362	*P. mediterranea*	F5	3	4	4	3	16
Pti212JX466363	*P. mediterranea*	F5	3	4	4	3	16
Pti213JX466364	*P. mediterranea*	F5	3	4	4	3	16
Pti214JX466365	*P. mediterranea*	F5	3	4	4	3	16
Pti215JX466366	*P. mediterranea*	F5	3	4	4	3	16
Pti216JX466367	*P. mediterranea*	F5	3	4	4	3	16
Pti217JX466368	*P. mediterranea*	F5	3	4	4	3	16
Pti219JX466346	*P. mediterranea*	F5	3	4	4	3	14
Pti220JX466347	*P. mediterranea*	F5	3	4	4	3	14
Pti222JX466349	*P. mediterranea*	F5	3	4	4	3	14
Pti223JX466350	*P. mediterranea*	F5	3	4	4	3	15
Pti226JX466353	*P. mediterranea*	F5	3	4	4	3	16
Pti227JX466354	*P. mediterranea*	F5	3	4	4	3	14
Pti228JX466336	*P. mediterranea*	F5	3	4	4	3	14
Pti229JX466337	*P. mediterranea*	F5	3	4	4	3	16
Pti22JX466320	*P. mediterranea*	F5	3	4	4	3	16
Pti230JX466338	*P. mediterranea*	F5	3	4	4	3	14
Pti231JX466339	*P. mediterranea*	F5	3	4	4	3	14
Pti232JX466340	*P. mediterranea*	F5	3	4	4	3	14
Pti234JX466342	*P. mediterranea*	F5	3	4	4	3	14
Pti236JX466344	*P. mediterranea*	F5	3	4	4	3	16
Pti2JF756982	*P. mediterranea*	F5	3	4	4	3	16
Pti307JX466420	*P. mediterranea*	F5	3	4	4	3	14
Pti308JX466421	*P. mediterranea*	F5	3	4	4	3	15

Sequence code	Species (current)	Clade/subclade	ASAP_ all_ rank1_ 8	ASAP_ all_ rank2_ 9	ABGD_ all_ 10	ASAP_ agg_ rank2_ 7	ABGD_ agg_ 42
Pti309JX466423	*P. mediterranea*	F5	3	4	4	3	14
Pti312JX466426	*P. mediterranea*	F5	3	4	4	3	14
Pti314JX466429	*P. mediterranea*	F5	3	4	4	3	14
Pti327KP659519	*P. mediterranea*	F5	3	4	4	3	16
Pti328KP659520	*P. mediterranea*	F5	3	4	4	3	14
Pti329KP659521	*P. mediterranea*	F5	3	4	4	3	14
Pti330KP659522	*P. mediterranea*	F5	3	4	4	3	14
Pti331KP659523	*P. mediterranea*	F5	3	4	4	3	14
Pti332KP659524	*P. mediterranea*	F5	3	4	4	3	16
Pti333KP659525	*P. mediterranea*	F5	3	4	4	3	14
Pti334KP659526	*P. mediterranea*	F5	3	4	4	3	14
Pti335KP659527	*P. mediterranea*	F5	3	4	4	3	14
Pti336KP659528	*P. mediterranea*	F5	3	4	4	3	14
Pti337KP659529	*P. mediterranea*	F5	3	4	4	3	16
Pti338KP659530	*P. mediterranea*	F5	3	4	4	3	16
Pti339KP659531	*P. mediterranea*	F5	3	4	4	3	14
Pti33JX466139	*P. mediterranea*	F5	3	4	4	3	16
Pti340KP659532	*P. mediterranea*	F5	3	4	4	3	14
Pti341KP659533	*P. mediterranea*	F5	3	4	4	3	14
Pti343KP659535	*P. mediterranea*	F5	3	4	4	3	14
Pti345KP659537	*P. mediterranea*	F5	3	4	4	3	14
Pti347KP659539	*P. mediterranea*	F5	3	4	4	3	14
Pti34JX466140	*P. mediterranea*	F5	3	4	4	3	16
Pti35JX466141	*P. mediterranea*	F5	3	4	4	3	16
Pti36JX466142	*P. mediterranea*	F5	3	4	4	3	16
Pti37JX466143	*P. mediterranea*	F5	3	4	4	3	16
Pti38JX466144	*P. mediterranea*	F5	3	4	4	3	16
Pti3JF756987	*P. mediterranea*	F5	3	4	4	3	14
Pti40JX466146	*P. mediterranea*	F5	3	4	4	3	16
Pti42JX466148	*P. mediterranea*	F5	3	4	4	3	16
Pti46JX466152	*P. mediterranea*	F5	3	4	4	3	16
Pti47JX466153	*P. mediterranea*	F5	3	4	4	3	16
Pti48JX466154	*P. mediterranea*	F5	3	4	4	3	16
Pti49JX466155	*P. mediterranea*	F5	3	4	4	3	16
Pti50JX466156	*P. mediterranea*	F5	3	4	4	3	16
Pti51JX466157	*P. mediterranea*	F5	3	4	4	3	14
Pti65JX466190	*P. mediterranea*	F5	3	4	4	3	18
Pti7190	*P. mediterranea*	F5	3	4	4	3	16
Pti7193	*P. mediterranea*	F5	3	4	4	3	16
Pti7194	*P. mediterranea*	F5	3	4	4	3	16

Sequence code	Species (current)	Clade/subclade	ASAP_ all_ rank1_ 8	ASAP_ all_ rank2_ 9	ABGD_ all_ 10	ASAP_ agg_ rank2_ 7	ABGD_ agg_ 42
Pti7201	*P. mediterranea*	F5	3	4	4	3	16
Pti7416	*P. mediterranea*	F5	3	4	4	3	14
Pti91JX466206	*P. mediterranea*	F5	3	4	4	3	16
Ppa10JX466290	*P. pastillifera*	F1	3	4	4	4	19
Ppa11JX466291	*P. pastillifera*	F1	3	4	4	5	20
Ppa12JX466292	*P. pastillifera*	F1	3	4	4	5	20
Ppa13JX466294	*P. pastillifera*	F1	3	4	4	5	20
Ppa14JX466295	*P. pastillifera*	F1	3	4	4	4	19
Ppa15JX466356	*P. pastillifera*	F1	3	4	4	4	19
Ppa16JX466357	*P. pastillifera*	F1	3	4	4	4	19
Ppa17JX466358	*P. pastillifera*	F1	3	4	4	5	20
Ppa18JX466359	*P. pastillifera*	F1	3	4	4	4	19
Ppa19JX466369	*P. pastillifera*	F1	3	4	4	4	19
Ppa1AY611104	*P. pastillifera*	F1	3	4	4	4	19
Ppa20JX466370	*P. pastillifera*	F1	3	4	4	4	19
Ppa21JX466371	*P. pastillifera*	F1	3	4	4	4	19
Ppa22JX466372	*P. pastillifera*	F1	3	4	4	4	19
Ppa23JX466373	*P. pastillifera*	F1	3	4	4	4	19
Ppa24JX466374	*P. pastillifera*	F1	3	4	4	4	19
Ppa25JX466375	*P. pastillifera*	F1	3	4	4	4	19
Ppa26JX466376	*P. pastillifera*	F1	3	4	4	4	19
Ppa27JX466377	*P. pastillifera*	F1	3	4	4	4	19
Ppa28JX466378	*P. pastillifera*	F1	3	4	4	4	19
Ppa29JX466387	*P. pastillifera*	F1	3	4	4	4	19
Ppa2DQ273860	*P. pastillifera*	F1	3	4	4	4	19
Ppa30JX466388	*P. pastillifera*	F1	3	4	4	4	19
Ppa31JX466413	*P. pastillifera*	F1	3	4	4	5	21
Ppa32JX466447	*P. pastillifera*	F1	3	4	4	5	20
Ppa33JX466448	*P. pastillifera*	F1	3	4	4	5	20
Ppa34JX466449	*P. pastillifera*	F1	3	4	4	5	20
Ppa35JX466455	*P. pastillifera*	F1	3	4	4	5	20
Ppa36JX466456	*P. pastillifera*	F1	3	4	4	5	20
Ppa37JX466458	*P. pastillifera*	F1	3	4	4	5	20
Ppa38JX466459	*P. pastillifera*	F1	3	4	4	5	20
Ppa39JX466460	*P. pastillifera*	F1	3	4	4	5	20
Ppa3JF756998	*P. pastillifera*	F1	3	4	4	4	19
Ppa40JX466461	*P. pastillifera*	F1	3	4	4	5	20
Ppa41JX466462	*P. pastillifera*	F1	3	4	4	5	20
Ppa42JX466422	*P. pastillifera*	F1	3	4	4	5	20
Ppa43JX466427	*P. pastillifera*	F1	3	4	4	5	20

Sequence code	Species (current)	Clade/subclade	ASAP_ all_ rank1_ 8	ASAP_ all_ rank2_ 9	ABGD_ all_ 10	ASAP_ agg_ rank2_ 7	ABGD_ agg_ 42
Ppa44JX466430	*P. pastillifera*	F1	3	4	4	4	22
Ppa45MK812633	*P. pastillifera*	F1	3	4	4	5	20
Ppa46MK812564	*P. pastillifera*	F1	3	4	4	5	20
Ppa47MK812554	*P. pastillifera*	F1	3	4	4	5	20
Ppa48MK812400	*P. pastillifera*	F1	3	4	4	5	20
Ppa49MK812365	*P. pastillifera*	F1	3	4	4	5	20
Ppa4JF756999	*P. pastillifera*	F1	3	4	4	4	19
Ppa50KX457685	*P. pastillifera*	F1	3	4	4	4	19
Ppa6JX466412	*P. pastillifera*	F1	3	4	4	4	19
Ppa7321	*P. pastillifera*	F1	3	4	4	5	20
Ppa7322	*P. pastillifera*	F1	3	4	4	4	19
Ppa7323	*P. pastillifera*	F1	3	4	4	4	19
Ppa7328	*P. pastillifera*	F1	3	4	4	4	19
Ppa7329	*P. pastillifera*	F1	3	4	4	4	19
Ppa7334	*P. pastillifera*	F1	3	4	4	4	19
Ppa7335	*P. pastillifera*	F1	3	4	4	4	19
Ppa7339	*P. pastillifera*	F1	3	4	4	4	19
Ppa7404	*P. pastillifera*	F1	3	4	4	4	19
Ppa7410	*P. pastillifera*	F1	3	4	4	5	20
Ppa7JX466457	*P. pastillifera*	F1	3	4	4	5	20
Ppa8JX466293	*P. pastillifera*	F1	3	4	4	5	23
Ppa9JX466289	*P. pastillifera*	F1	3	4	4	4	19
Pqu10HM017024	*P. quercina*	C	6	7	8	-	-
Pqu10JF757002	*P. quercina*	C	6	7	8	-	-
Pqu12HM017021	*P. quercina*	C	6	7	8	-	-
Pqu1DQ273842	*P. quercina*	C	6	7	8	-	-
Pqu2DQ273843	*P. quercina*	C	6	7	8	-	-
Pqu3DQ273844	*P. quercina*	C	6	7	8	-	-
Pqu4DQ273845	*P. quercina*	C	6	7	8	-	-
Pqu5DQ273846	*P. quercina*	C	6	7	8	-	-
Pqu6DQ273847	*P. quercina*	C	6	7	8	-	-
Pqu7DQ273848	*P. quercina*	C	6	7	8	-	-
Pqu8HM017022	*P. quercina*	C	6	7	8	-	-
Pqu8JF757000	*P. quercina*	C	6	7	8	-	-
Pqu9HM017023	*P. quercina*	C	6	7	8	-	-
Pqu9JF757001	*P. quercina*	C	6	7	8	-	-
Pque6987	*P. quercina*	C	6	7	8	-	-
Pque7038	*P. quercina*	C	6	7	8	-	-
Pque7039	*P. quercina*	C	6	7	8	-	-
Pat7386	*P. tiliacea*	F3	3	4	4	3	24

Sequence code	Species (current)	Clade/subclade	ASAP_ all_ rank1_ 8	ASAP_ all_ rank2_ 9	ABGD_ all_ 10	ASAP_ agg_ rank2_ 7	ABGD_ agg_ 42
Pti112JX466218	*P. tiliacea*	F3	3	4	4	3	25
Pti116JX466222	*P. tiliacea*	F3	3	4	4	3	26
Pti11JF756989	*P. tiliacea*	F3	3	4	4	3	26
Pti12JF756991	*P. tiliacea*	F3	3	4	4	3	26
Pti133JX466238	*P. tiliacea*	F3	3	4	4	3	26
Pti135JX466240	*P. tiliacea*	F3	3	4	4	3	26
Pti137JX466242	*P. tiliacea*	F3	3	4	4	3	27
Pti151JX466257	*P. tiliacea*	F3	3	4	4	3	26
Pti153JX466261	*P. tiliacea*	F3	3	4	4	3	26
Pti154JX466262	*P. tiliacea*	F3	3	4	4	3	26
Pti155JX466263	*P. tiliacea*	F3	3	4	4	3	26
Pti156JX466258	*P. tiliacea*	F3	3	4	4	3	28
Pti170JX466269	*P. tiliacea*	F3	3	4	4	3	29
Pti171JX466270	*P. tiliacea*	F3	3	4	4	3	26
Pti172JX466271	*P. tiliacea*	F3	3	4	4	3	26
Pti173JX466272	*P. tiliacea*	F3	3	4	4	3	26
Pti174JX466273	*P. tiliacea*	F3	3	4	4	3	26
Pti175JX466274	*P. tiliacea*	F3	3	4	4	3	26
Pti176JX466275	*P. tiliacea*	F3	3	4	4	3	26
Pti177JX466276	*P. tiliacea*	F3	3	4	4	3	26
Pti178JX466277	*P. tiliacea*	F3	3	4	4	3	26
Pti179JX466278	*P. tiliacea*	F3	3	4	4	3	26
Pti181JX466288	*P. tiliacea*	F3	3	4	4	3	26
Pti182JX466296	*P. tiliacea*	F3	3	4	4	3	26
Pti201JX466315	*P. tiliacea*	F3	3	4	4	3	30
Pti218JX466345	*P. tiliacea*	F3	3	4	4	3	26
Pti21JX466324	*P. tiliacea*	F3	3	4	4	3	31
Pti224JX466351	*P. tiliacea*	F3	3	4	4	3	26
Pti233JX466341	*P. tiliacea*	F3	3	4	4	3	26
Pti239JX466325	*P. tiliacea*	F3	3	4	4	3	26
Pti240JX466326	*P. tiliacea*	F3	3	4	4	3	26
Pti242JX466327	*P. tiliacea*	F3	3	4	4	3	26
Pti243JX466328	*P. tiliacea*	F3	3	4	4	3	32
Pti244JX466329	*P. tiliacea*	F3	3	4	4	3	26
Pti245JX466330	*P. tiliacea*	F3	3	4	4	3	26
Pti246JX466331	*P. tiliacea*	F3	3	4	4	3	26
Pti247JX466332	*P. tiliacea*	F3	3	4	4	3	26
Pti248JX466333	*P. tiliacea*	F3	3	4	4	3	26
Pti249JX466334	*P. tiliacea*	F3	3	4	4	3	26
Pti24JX466130	*P. tiliacea*	F3	3	4	4	3	33

Sequence code	Species (current)	Clade/subclade	ASAP_ all_ rank1_ 8	ASAP_ all_ rank2_ 9	ABGD_ all_ 10	ASAP_ agg_ rank2_ 7	ABGD_ agg_ 42
Pti250JX466335	*P. tiliacea*	F3	3	4	4	3	32
Pti253JX466381	*P. tiliacea*	F3	3	4	4	3	26
Pti267JX466397	*P. tiliacea*	F3	3	4	4	3	32
Pti268JX466398	*P. tiliacea*	F3	3	4	4	3	26
Pti269JX466399	*P. tiliacea*	F3	3	4	4	3	34
Pti270JX466400	*P. tiliacea*	F3	3	4	4	3	26
Pti271JX466401	*P. tiliacea*	F3	3	4	4	3	34
Pti272JX466402	*P. tiliacea*	F3	3	4	4	3	26
Pti273JX466403	*P. tiliacea*	F3	3	4	4	3	34
Pti276JX466406	*P. tiliacea*	F3	3	4	4	3	34
Pti278JX466408	*P. tiliacea*	F3	3	4	4	3	32
Pti279JX466409	*P. tiliacea*	F3	3	4	4	3	32
Pti281JX466411	*P. tiliacea*	F3	3	4	4	3	32
Pti282JX466414	*P. tiliacea*	F3	3	4	4	3	26
Pti283JX466415	*P. tiliacea*	F3	3	4	4	3	26
Pti284JX466416	*P. tiliacea*	F3	3	4	4	3	34
Pti286JX466443	*P. tiliacea*	F3	3	4	4	3	26
Pti290JX466450	*P. tiliacea*	F3	3	4	4	3	26
Pti291JX466451	*P. tiliacea*	F3	3	4	4	3	26
Pti292JX466452	*P. tiliacea*	F3	3	4	4	3	26
Pti293JX466453	*P. tiliacea*	F3	3	4	4	3	26
Pti294JX466454	*P. tiliacea*	F3	3	4	4	3	26
Pti295JX466463	*P. tiliacea*	F3	3	4	4	3	32
Pti296JX466464	*P. tiliacea*	F3	3	4	4	3	32
Pti297JX466465	*P. tiliacea*	F3	3	4	4	3	26
Pti298JX466466	*P. tiliacea*	F3	3	4	4	3	26
Pti299JX466467	*P. tiliacea*	F3	3	4	4	3	26
Pti300JX466468	*P. tiliacea*	F3	3	4	4	3	26
Pti301JX466469	*P. tiliacea*	F3	3	4	4	3	26
Pti302JX466470	*P. tiliacea*	F3	3	4	4	3	26
Pti303JX466471	*P. tiliacea*	F3	3	4	4	3	26
Pti304JX466472	*P. tiliacea*	F3	3	4	4	3	26
Pti310JX466424	*P. tiliacea*	F3	3	4	4	3	26
Pti315JX466431	*P. tiliacea*	F3	3	4	4	3	35
Pti316JX466432	*P. tiliacea*	F3	3	4	4	3	26
Pti318JX466434	*P. tiliacea*	F3	3	4	4	3	26
Pti319JX466435	*P. tiliacea*	F3	3	4	4	3	26
Pti320JX466436	*P. tiliacea*	F3	3	4	4	3	26
Pti321JX466437	*P. tiliacea*	F3	3	4	4	3	36
Pti322JX466438	*P. tiliacea*	F3	3	4	4	3	26

Sequence code	Species (current)	Clade/subclade	ASAP_ all_ rank1_ 8	ASAP_ all_ rank2_ 9	ABGD_ all_ 10	ASAP_ agg_ rank2_ 7	ABGD_ agg_ 42
Pti324JX466440	*P. tiliacea*	F3	3	4	4	3	26
Pti348KP659540	*P. tiliacea*	F3	3	4	4	3	26
Pti349KP659541	*P. tiliacea*	F3	3	4	4	3	35
Pti350KP659542	*P. tiliacea*	F3	3	4	4	3	26
Pti354KP659546	*P. tiliacea*	F3	3	4	4	3	26
Pti355KP659547	*P. tiliacea*	F3	3	4	4	3	26
Pti356KP659548	*P. tiliacea*	F3	3	4	4	3	26
Pti357KP659549	*P. tiliacea*	F3	3	4	4	3	26
Pti358KP659550	*P. tiliacea*	F3	3	4	4	3	26
Pti359KP659551	*P. tiliacea*	F3	3	4	4	3	26
Pti360KP659552	*P. tiliacea*	F3	3	4	4	3	26
Pti362KP659554	*P. tiliacea*	F3	3	4	4	3	37
Pti363KP659555	*P. tiliacea*	F3	3	4	4	3	26
Pti364KP659556	*P. tiliacea*	F3	3	4	4	3	26
Pti365KP659557	*P. tiliacea*	F3	3	4	4	3	26
Pti366KP659558	*P. tiliacea*	F3	3	4	4	3	26
Pti367KP659559	*P. tiliacea*	F3	3	4	4	3	26
Pti368KP659560	*P. tiliacea*	F3	3	4	4	3	26
Pti372MK812562	*P. tiliacea*	F3	3	4	4	3	34
Pti374MK812472	*P. tiliacea*	F3	3	4	4	3	34
Pti376MK812259	*P. tiliacea*	F3	3	4	4	3	32
Pti378MK811628	*P. tiliacea*	F3	3	4	4	3	34
Pti379MK629945	*P. tiliacea*	F3	3	4	4	3	32
Pti380KX132912	*P. tiliacea*	F3	3	4	4	3	26
Pti43JX466149	*P. tiliacea*	F3	3	4	4	6	38
Pti44JX466150	*P. tiliacea*	F3	3	4	4	6	38
Pti45JX466151	*P. tiliacea*	F3	3	4	4	6	38
Pti4JF756984	*P. tiliacea*	F3	3	4	4	3	27
Pti52JX466177	*P. tiliacea*	F3	3	4	4	3	27
Pti53JX466178	*P. tiliacea*	F3	3	4	4	6	38
Pti54JX466179	*P. tiliacea*	F3	3	4	4	6	38
Pti55JX466180	*P. tiliacea*	F3	3	4	4	6	38
Pti56JX466181	*P. tiliacea*	F3	3	4	4	6	38
Pti57JX466182	*P. tiliacea*	F3	3	4	4	6	38
Pti58JX466183	*P. tiliacea*	F3	3	4	4	6	38
Pti59JX466184	*P. tiliacea*	F3	3	4	4	6	38
Pti60JX466185	*P. tiliacea*	F3	3	4	4	6	38
Pti61JX466186	*P. tiliacea*	F3	3	4	4	3	27
Pti62JX466187	*P. tiliacea*	F3	3	4	4	6	38
Pti63JX466188	*P. tiliacea*	F3	3	4	4	6	38

Sequence code	Species (current)	Clade/subclade	ASAP_ all_ rank1_ 8	ASAP_ all_ rank2_ 9	ABGD_ all_ 10	ASAP_ agg_ rank2_ 7	ABGD_ agg_ 42
Pti64JX466189	*P. tiliacea*	F3	3	4	4	6	38
Pti66JX466191	*P. tiliacea*	F3	3	4	4	6	38
Pti67JX466192	*P. tiliacea*	F3	3	4	4	6	38
Pti68JX466193	*P. tiliacea*	F3	3	4	4	6	38
Pti69JX466194	*P. tiliacea*	F3	3	4	4	6	38
Pti70JX466195	*P. tiliacea*	F3	3	4	4	6	38
Pti7318	*P. tiliacea*	F3	3	4	4	7	39
Pti7319	*P. tiliacea*	F3	3	4	4	7	39
Pti7327	*P. tiliacea*	F3	3	4	4	7	39
Pti7332	*P. tiliacea*	F3	3	4	4	3	40
Pti7341	*P. tiliacea*	F3	3	4	4	7	39
Pti7349	*P. tiliacea*	F3	3	4	4	7	39
Pti7350	*P. tiliacea*	F3	3	4	4	7	39
Pti7351	*P. tiliacea*	F3	3	4	4	7	39
Pti7356	*P. tiliacea*	F3	3	4	4	3	26
Pti7358	*P. tiliacea*	F3	3	4	4	3	26
Pti7360	*P. tiliacea*	F3	3	4	4	7	39
Pti7361	*P. tiliacea*	F3	3	4	4	7	39
Pti7398	*P. tiliacea*	F3	3	4	4	7	41
Pti7407	*P. tiliacea*	F3	3	4	4	3	40
Pti7409	*P. tiliacea*	F3	3	4	4	3	26
Pti7412	*P. tiliacea*	F3	3	4	4	3	26
Pti7413	*P. tiliacea*	F3	3	4	4	3	26
Pti7415	*P. tiliacea*	F3	3	4	4	3	26
Pti77JX466164	*P. tiliacea*	F3	3	4	4	3	27
Pti81JX466168	*P. tiliacea*	F3	3	4	4	3	26
Pti84JX466171	*P. tiliacea*	F3	3	4	4	3	27
Pti8JF756983	*P. tiliacea*	F3	3	4	4	6	38
Pti90JX466196	*P. tiliacea*	F3	3	4	4	6	38
Pti94JX466199	*P. tiliacea*	F3	3	4	4	3	42
Pya1GU979823	*P. yalungana*	A1	7	8	9	-	-
Pya2GU979824	*P. yalungana*	A1	7	8	9	-	-
Pya3GU979821	*P. yalungana*	A1	7	8	9	-	-
PtiCH060016	*Parmelina sp.*	A2	8	9	10	-	-

Table S3

Table S3. Ultrafast bootstrap (UFBoot) and Bayesian posterior probability values for every node of the tree shown in Figure 1. A dash indicates a node that did not reach the reporting threshold.

Node (Figure 1)	UFBoot (%)	Posterior probability
97	-	-
98	100	0.647
99	72	0.998
100	90	0.765
101	79	1
102	99	1
103	99	1
104	99	-
105	99	1
106	30	-
107	21	-
108	25	-
109	21	-
110	99	0.82
111	95	-
112	23	-
113	18	-
114	19	-
115	18	-
116	21	-
117	98	0.921
118	22	-
119	24	-
120	28	-
121	21	-
122	98	0.935
123	48	-
124	49	-
125	95	0.911
126	97	0.999
127	63	-
128	66	-
129	99	1
130	87	0.715
131	65	-
132	99	1
133	98	0.771
134	28	-

Node (Figure 1)	UFBoot (%)	Posterior probability
135	32	-
136	17	-
137	27	-
138	23	-
139	25	-
140	16	-
141	19	-
142	11	-
143	18	-
144	11	-
145	22	-
146	99	0.954
147	99	0.971
148	28	-
149	27	-
150	30	-
151	37	-
152	98	0.932
153	23	-
154	50	-
155	59	-
156	50	0.909
157	78	0.982
158	41	-
159	31	-
160	34	-
161	84	1
162	66	0.763
163	59	-
164	97	0.829
165	97	0.999
166	31	-
167	31	-
168	89	1
169	60	-
170	45	-
171	95	0.899
172	89	0.704
173	89	0.511
174	46	-
175	100	1
176	63	-

Node (Figure 1)	UFBoot (%)	Posterior probability
177	98	0.979
178	56	-
179	52	-
180	100	1
181	60	-
182	100	1
183	83	-
184	99	0.972
185	82	-
186	98	0.966
187	82	-
188	83	-
189	84	0.927
190	100	1
191	-	1
